# Supplementary material for: Antibiotics confound breath-based respiratory disease detection in calves
Source: PLoS One. 2026 Jun 24;21(6):e0351838. doi: 10.1371/journal.pone.0351838 (PMC13293443; doi:10.1371/journal.pone.0351838)
Supplement: S1 File — (DOCX) [file pone.0351838.s001.docx]

**Supplementary Information for:**

**Antibiotics Confound Breath-Based Respiratory Disease Detection in Calves**

Ben Langford^1^, Johanna Brans^2^, Claire Broadbent^2^, Tim Gibson^3^, Mark Hanlon^1^_,_ Marie Haskell^2^, Laura Nicoll^2^, Neil J. Mullinger^1^ and Carol-Anne Duthie^2^

**Table S1. Summary of cattle included in the study, including sex, ID, average respiration rate, start and end body weights. Also shown are the number of breath samples collected during ‘true’ healthy periods (defined as no clinical disease ± 4 days) and during diseased periods.**

| **Cattle ID** | **Sex** | **Breed** | **Start Weight [kg]** | **End Weight [kg]** | **Healthy Samples [#]** | **Diseased Period [#]** | **RR [bpm]** |
| --- | --- | --- | --- | --- | --- | --- | --- |
| UK581493110709 | F | Hereford X | 54.0 | 92 | 3 | 0 | 35.9 |
| UK581493110716 | F | Hereford X | 54.2 | 91.4 | 2 | 0 | 37.5 |
| UK581493310711 | F | Hereford X | 51.6 | 91.8 | 3 | 0 | 39.4 |
| UK581493410712 | F | Hereford X | 51.2 | 95.8 | 3 | 0 | 30.5 |
| UK581493510713 | F | Hereford X | 48.6 | 87.8 | 2 | 0 | 34.9 |
| UK581493610714 | F | Hereford X | 40.2 | 78.6 | 3 | 0 | 27.6 |
| UK581493710708 | F | Hereford X | 60.6 | 105.8 | 3 | 0 | 33.7 |
| UK102505602603 | F | Hereford X | 51.6 | 88.6 | 4 | 0 | 37.8 |
| UK102505202606 | M | Hereford | 48.4 | 84.6 | 3 | 5 | 39.1 |
| UK102505102598 | M | Hereford | 49.8 | 82.6 | 3 | 0 | 35.3 |
| UK102505602596 | F | Hereford X | 42.2 | 72.6 | 1 | 0 | 39.3 |
| UK101232612755 | F | Aberdeen Angus X | 58.8 | 99.0 | 3 | 0 | 38.7 |
| UK101232112729 | F | Aberdeen Angus X | 66.0 | 113.6 | 4 | 0 | 34.7 |
| UK101319504941 | F | Hereford X | 59.4 | 102.8 | 3 | 5 | 34.3 |
| UK101319204945 | F | Hereford X | 60.6 | 104.8 | 3 | 0 | 35.4 |
| UK101319304946 | F | Hereford X | 59.4 | 110.4 | 2 | 0 | 37.9 |
| UK582658309499 | M | Aberdeen Angus X | 48.8 | 91.2 | 2 | 5 | 30.1 |
| UK582658709489 | F | Aberdeen Angus X | 57.0 | 102.0 | 4 | 0 | 36.9 |
| UK582658609495 | M | Aberdeen Angus X | 52.2 | 96.2 | 4 | 0 | 38.4 |
| UK582658709503 | M | Aberdeen Angus X | 44.6 | 83.0 | 3 | 0 | 35.7 |
| UK582658109504 | F | Hereford X | 45.6 | 94.2 | 4 | 0 | 31.2 |
| UK582658409486 | M | Aberdeen Angus X | 42.0 | 96.2 | 4 | 0 | 32.3 |
| UK582658609502 | M | Aberdeen Angus X | 48.6 | 99.4 | 4 | 0 | 34.7 |
| UK582658109490 | F | Hereford X | 62.8 | 104.8 | 0 | 4 | 35.4 |
| UK582658209498 | F | Hereford X | 46.8 | 87.4 | 1 | 3 | 34.1 |
| UK582658709496 | M | Aberdeen Angus X | 53 | 93.4 | 4 | 0 | 34.8 |
| UK582997508744 | M | Aberdeen Angus X | 54.6 | 97 | 3 | 4 | 34.3 |
| UK582997408743 | M | Aberdeen Angus X | 47.2 | 83.4 | 3 | 0 | 32.7 |
| UK582997308742 | M | Aberdeen Angus X | 42.8 | 76.4 | 3 | 0 | 30.8 |
| UK582997208741 | M | Aberdeen Angus X | 44.6 | 71.2 | 4 | 0 | 36.0 |
| UK582997608752 | F | Hereford X | 50.6 | 89.2 | 4 | 0 | 33.5 |
| UK582997708753 | M | Hereford X | 49.6 | 78.2 | 6 | 0 | 36.2 |
| UK582997108754 | M | Aberdeen Angus X | 45.8 | 88.4 | 5 | 0 | 33.2 |
| UK582997208755 | M | Aberdeen Angus X | 53.2 | 95.2 | 2 | 4 | 30.1 |
| UK582997308756 | M | Aberdeen Angus X | 43.6 | 80.2 | 3 | 0 | 30.7 |
| UK561505405393 | M | Aberdeen Angus X | 51.8 | 88.8 | 4 | 0 | 34.8 |
| UK561505405400 | F | British Blue X | 50.4 | 88.4 | 4 | 0 | 29.7 |
| UK561505605402 | F | Aberdeen Angus X | 48.8 | 87.2 | 5 | 0 | 32.4 |
| UK561505605409 | F | British Blue X | 45.2 | 78.2 | 5 | 0 | 39.6 |
| UK561505205405 | F | British Blue X | 49.4 | 69.6 | 5 | 0 | 34.1 |
| UK581493310921 | M | Aberdeen Angus X | 48.8 | 84.8 | 5 | 5 | 41.0 |
| UK581493610917 | M | Aberdeen Angus X | 49.6 | 89.8 | 4 | 5 | 37.3 |
| UK581493610924 | F | Aberdeen Angus X | 45 | 79 | 5 | 0 | 37.7 |
| UK581493510923 | M | British Blue X | 55 | 81.2 | 1 | 5 | 36.1 |
| UK581493710911 | F | British Blue X | 50.4 | 82.6 | 3 | 0 | 33.5 |
| UK561505205398 | M | British Blue X | 65 | 95.6 | 4 | 0 | 34.1 |
| UK561505405407 | M | British Blue X | 55.2 | 74.8 | 4 | 0 | 32.8 |
| UK581493610910 | M | British Blue X | 68.4 | 106.8 | 6 | 0 | 36.5 |
| UK581493110912 | M | Aberdeen Angus X | 60.8 | 101.6 | 3 | 4 | 39.7 |
| UK581493310914 | M | British Blue X | 60.6 | 94.8 | 2 | 5 | 37.0 |
| UK581493510909 | M | British Blue X | 57.2 | 84.2 | 3 | 0 | 31.5 |
| UK582658309898 | M | Aberdeen Angus X | 48.4 | 98.6 | 5 | 5 | 33.1 |
| UK581723104715 | F | British Blue X | 50.2 | 91.6 | 5 | 0 | 38.5 |
| UK582658509900 | M | Aberdeen Angus X | 44.8 | 93.8 | 5 | 0 | 33.3 |
| UK582658409899 | M | Aberdeen Angus X | 40.8 | 87.2 | 5 | 0 | 36.0 |
| UK582658609901 | M | Aberdeen Angus X | 42.6 | 92 | 4 | 0 | 33.1 |
| UK101232313319 | F | British Blue X | 58.6 | 61.8 | 1 | 5 | 35.5 |
| UK101232713316 | F | British Blue X | 51.2 | 73.2 | 0 | 5 | 30.4 |
| UK101232713323 | M | British Blue X | 43.4 | 66.8 | 1 | 4 | 33.9 |
| UK101232113317 | M | Aberdeen Angus X | 54 | 83.4 | 2 | 5 | 33.5 |
| UK101232513321 | M | Aberdeen Angus X | 41.8 | 64.8 | 0 | 10 | 33.8 |
| UK101232413313 | M | British Blue X | 54.8 | 73.2 | 3 | 0 | 33.9 |
| UK101232613322 | F | Aberdeen Angus X | 47.6 | 62.8 | 0 | 5 | 34.0 |
|  |  |  |  |  |  |  |  |
| UK101232313312 | M | British Blue X | 61.6 | 76.4 | 0 | 5 | 37.2 |
| UK101232613315 | F | British Blue X | 39.4 | 57.6 | 3 | 5 | 29.6 |

**Table S2. List of 86 ions detected in the breath of cattle (both healthy and diseased), presented with their monoisotopic mass-to-charge ratios (m/z), proposed protonated ion formulas, and linear regression parameters describing the relationship between volatile organic compound (VOC) emission rate and age in the healthy cohort. Emission rates (*y*) are expressed in units of mg animal⁻¹ h⁻¹, and age (days) is given in days. For each ion, the slope (*b*), intercept (*a*), 95% confidence interval for the slope, and coefficient of determination (r^2^) are reported. The linear model takes the form *y*=*a*+bx.**

| **Formula** | ***m/z*** |  | **Linear** |  |  |  |
| --- | --- | --- | --- | --- | --- | --- |
|  |  | ***a*** | **±** | ***b*** | **±** | **R^2^** |
| (CH_2_O )H^+^ | 31.0180 | 0.010136 | 0.006557 | 0.000519 | 0.000149 | 0.20 |
| (CH_4_O)H^+^ | 33.0330 | 0.00769 | 0.003792 | 0.00023 | 8.62E-05 | 0.13 |
| (H_3_O^+^(H_2_O)+ | 37.0280 | 0.01032 | 0.011972 | 0.000502 | 0.000272 | 0.07 |
| (C_3_H_2_)H^+^ | 39.0230 | 0.001662 | 0.001408 | 0.000108 | 3.2E-05 | 0.19 |
| 39.036053 | 39.0361 | 7.99E-05 | 3.03E-05 | 1.57E-06 | 6.89E-07 | 0.10 |
| 40.026287 | 40.0263 | 5.51E-05 | 4.25E-05 | 3.27E-06 | 9.67E-07 | 0.19 |
| 41.02903 | 41.0290 | 0.000107 | 0.000134 | 1.09E-05 | 3.05E-06 | 0.21 |
| (C_3_H_4_)H^+^ | 41.0386 | 0.002752 | 0.001991 | 0.000166 | 4.53E-05 | 0.22 |
| (C_2_H_3_N)H^+^ | 42.0338 | 0.002138 | 0.000541 | 1.69E-05 | 1.23E-05 | 0.04 |
| (C_2_H_2_O)H^+^ | 43.0178 | 0.001555 | 0.000913 | 1.88E-05 | 2.08E-05 | 0.02 |
| 43.042877 | 43.0429 | 3.04E-05 | 1.69E-05 | 1.44E-06 | 3.85E-07 | 0.23 |
| (C_3_H_6_)H^+^ | 43.0540 | -4.6E-05 | 0.000241 | 1.82E-05 | 5.49E-06 | 0.19 |
| 43.989445 | 43.9894 | 0.01043 | 0.002316 | 0.000201 | 5.26E-05 | 0.23 |
| (C_2_H_4_O)+ | 44.0240 | 0.000108 | 5.03E-05 | 2.3E-06 | 1.14E-06 | 0.08 |
| (CO_2_)H^+^ | 44.9971 | 40835.16 | 8015.248 | 667.672 | 184.6548 | 0.22 |
| 45.014637 | 45.0146 | 0.025408 | 0.005601 | 0.000382 | 0.000127 | 0.16 |
| 45.023479 | 45.0235 | 0.013694 | 0.003925 | 0.000158 | 8.92E-05 | 0.06 |
| (C_2_H_4_O)H^+^ | 45.0335 | 0.001455 | 0.00101 | -6.2E-06 | 2.3E-05 | 0.00 |
| 45.991646 | 45.9916 | 0.000658 | 0.000229 | 2.14E-05 | 5.2E-06 | 0.26 |
| 46.001053 | 46.0011 | 0.008662 | 0.001802 | 0.000164 | 4.1E-05 | 0.25 |
| 47.01754 | 47.0175 | -0.00018 | 0.00017 | 1.91E-05 | 3.86E-06 | 0.34 |
| (C_2_H_6_O)H^+^ | 47.0490 | 0.000211 | 0.000324 | -1.4E-06 | 7.36E-06 | 0.00 |
| (CH_4_S)H^+^ | 48.0034 | 8.95E-05 | 0.000418 | 2.52E-05 | 9.49E-06 | 0.13 |
| 48.004822 | 48.0048 | 0.000111 | 5.39E-05 | 4.77E-06 | 1.22E-06 | 0.24 |
| (O_3_)H^+^ | 48.9920 | 4.5E-06 | 3.37E-05 | 2.16E-06 | 7.66E-07 | 0.14 |
| (CH_4_S)H^+^ | 49.0106 | 4.84E-05 | 3.49E-05 | 7.68E-07 | 7.93E-07 | 0.02 |
| (CH_3_O-H_2_O)H^+^ | 51.0450 | 1.94E-05 | 9.42E-06 | 6.3E-07 | 2.14E-07 | 0.15 |
| 53.002232 | 53.0022 | 4.59E-05 | 3.62E-05 | 2.46E-06 | 8.24E-07 | 0.16 |
| 55.023628 | 55.0236 | 2.26E-05 | 1.06E-05 | 8.77E-07 | 2.42E-07 | 0.22 |
| 55.041302 | 55.0413 | 4.76E-06 | 9.61E-05 | 7.12E-06 | 2.18E-06 | 0.18 |
| (C_4_H_6_)H^+^ | 55.0540 | -0.00053 | 0.001641 | 0.000118 | 3.73E-05 | 0.17 |
| 56.044266 | 56.0443 | 1.12E-05 | 4.29E-06 | 4.31E-07 | 9.74E-08 | 0.29 |
| 56.056999 | 56.0570 | -1.2E-05 | 7.22E-05 | 5.15E-06 | 1.64E-06 | 0.17 |
| (C_3_H_4_O)H^+^ | 57.0330 | 0.000109 | 6.65E-05 | 1.32E-06 | 1.51E-06 | 0.02 |
| (C_4_H_8_)H^+^ | 57.0699 | 0.000134 | 7.08E-05 | 4.09E-06 | 1.61E-06 | 0.12 |
| (C_3_H_6_O)^+^ | 58.0410 | 4.19E-05 | 3.77E-05 | 3.25E-06 | 8.56E-07 | 0.23 |
| (C_3_H_6_O)H^+^ | 59.0490 | 0.049089 | 0.044648 | 0.003955 | 0.001015 | 0.24 |
| 60.038895 | 60.0389 | 0.000103 | 9.28E-05 | 7.47E-06 | 2.11E-06 | 0.21 |
| 60.052483 | 60.0525 | 0.001642 | 0.001416 | 0.000128 | 3.22E-05 | 0.25 |
| 60.995735 | 60.9957 | 9.26E-06 | 4.5E-05 | 2.75E-06 | 1.02E-06 | 0.13 |
| (C_2_H_4_S)H^+^ | 61.0106 | 4.75E-05 | 0.000732 | 4.47E-05 | 1.66E-05 | 0.13 |
| 61.053482 | 61.0535 | 0.000127 | 0.000102 | 8.86E-06 | 2.31E-06 | 0.24 |
| (CH_3_NO_2_)H^+^ | 62.0237 | 0.000442 | 0.000142 | 6.02E-06 | 3.24E-06 | 0.07 |
| (C_2_H_6_S)H^+^ | 63.0260 | -0.00028 | 0.007465 | 0.000488 | 0.00017 | 0.15 |
| 64.011726 | 64.0117 | 0.000255 | 7.83E-05 | -1.1E-06 | 1.78E-06 | 0.01 |
| 64.028793 | 64.0288 | -1.9E-05 | 0.00022 | 1.45E-05 | 5.01E-06 | 0.15 |
| 65.005394 | 65.0054 | 8.68E-05 | 3.79E-05 | 9.49E-07 | 8.62E-07 | 0.02 |
| (CH_4_O_3_)H^+^ | 65.0230 | 2.54E-05 | 0.000327 | 2.11E-05 | 7.43E-06 | 0.14 |
| (CH_2_N_2_Na)H^+^ | 66.0188 | 7.31E-06 | 4.47E-06 | 3.83E-07 | 1.02E-07 | 0.23 |
| (C_5_H_6_)H^+^ | 67.0540 | 8.13E-05 | 2.79E-05 | 1.55E-06 | 6.35E-07 | 0.11 |
| 68.05304 | 68.0530 | 1.36E-05 | 2.94E-06 | 3.02E-07 | 6.68E-08 | 0.30 |
| (C_5_H_8_)H^+^ | 69.0699 | 0.000531 | 0.000166 | 1.77E-06 | 3.77E-06 | 0.00 |
| (C_5_H_10_)H^+^)H^+^ | 71.0855 | 2.38E-05 | 1.12E-05 | 9.49E-07 | 2.56E-07 | 0.22 |
| (C_3_H_4_O_2_)H^+^ | 73.0280 | 2.27E-05 | 1.05E-05 | 6.78E-07 | 2.39E-07 | 0.14 |
| ((H_2_O)_4_)H^+^ | 73.0500 | -6.6E-05 | 0.000156 | 1.06E-05 | 3.55E-06 | 0.16 |
| (C_4_H_8_O)H^+^ | 73.0648 | -0.00149 | 0.002395 | 0.000169 | 5.44E-05 | 0.17 |
| 74.064522 | 74.0645 | -1.4E-05 | 9.76E-05 | 5.75E-06 | 2.22E-06 | 0.12 |
| (C_3_H_8_O_2_)H^+^ | 77.0597 | 0.00035 | 0.000587 | 4.6E-05 | 1.34E-05 | 0.20 |
| 78.062981 | 78.0630 | 1.37E-05 | 1.98E-05 | 1.56E-06 | 4.5E-07 | 0.20 |
| 80.052383 | 80.0524 | 1.35E-05 | 4.12E-06 | 4.52E-07 | 9.36E-08 | 0.33 |
| (C_4_H_4_N_2_)H^+^ | 81.0447 | -9.8E-05 | 3.8E-05 | 5.08E-06 | 8.64E-07 | 0.42 |
| (C_6_H_10_)H^+^ | 83.0855 | 0.00026 | 9.07E-05 | 1.6E-06 | 2.06E-06 | 0.01 |
| 84.087402 | 84.0874 | 2.67E-05 | 5.64E-06 | 4.44E-07 | 1.28E-07 | 0.20 |
| (C_6_H_12_)H^+^ | 85.1010 | 2.63E-05 | 4.49E-06 | 2.27E-07 | 1.02E-07 | 0.09 |
| (C_5_H_10_O)H^+^ | 87.0800 | 3.29E-05 | 9.93E-06 | 3.79E-07 | 2.26E-07 | 0.06 |
| (C_4_H_10_O_2_)H^+^ | 91.0750 | -4.5E-05 | 8.65E-05 | 5.12E-06 | 1.97E-06 | 0.12 |
| (C_7_H_12_)H^+^ | 97.1010 | 7.24E-05 | 1.68E-05 | 5.96E-07 | 3.81E-07 | 0.05 |
| (C_7_H_5_N)H^+^ | 104.0490 | 0.0001 | 1.79E-05 | -6.2E-07 | 4.06E-07 | 0.05 |
| (C_4_H_10_ClN)H^+^ | 108.0575 | 3.53E-05 | 7.47E-06 | 1.19E-07 | 1.7E-07 | 0.01 |
| 109.100975 | 109.1010 | 0.000122 | 3.56E-05 | 4.31E-07 | 8.09E-07 | 0.01 |
| 116.905487 | 116.9055 | 3.53E-05 | 1.12E-05 | 9.28E-07 | 2.55E-07 | 0.22 |
| (C_6_H_12_O_2_)H^+^ | 117.0910 | 1.62E-05 | 3.98E-06 | 3.9E-07 | 9.04E-08 | 0.28 |
| (C_5_H_8_FNO)H^+^ | 118.0663 | 3.19E-05 | 5.78E-05 | 5.83E-07 | 1.31E-06 | 0.00 |
| (C_9_H_10_)H^+^ | 119.0855 | 4.7E-05 | 1.08E-05 | -2.3E-09 | 2.45E-07 | 0.00 |
| (C_4_H_8_FNO_2_)H^+^ | 122.0612 | 2.55E-05 | 6.83E-06 | 3.15E-07 | 1.55E-07 | 0.08 |
| (C_4_H_10_O_2_S)H^+^ | 123.0470 | 3.51E-05 | 6.92E-06 | 1.04E-08 | 1.57E-07 | 0.00 |
| 123.116577 | 123.1166 | 7.89E-05 | 2.04E-05 | 9.41E-07 | 4.64E-07 | 0.08 |
| (C_9_H_16_)H^+^ | 125.1320 | 3.4E-05 | 5.42E-06 | 2.55E-07 | 1.23E-07 | 0.08 |
| (C_8_H_14_O)H^+^ | 127.1117 | 2.39E-05 | 5.1E-06 | 3.16E-07 | 1.16E-07 | 0.14 |
| (C_5_H_12_O_2_Si)H^+^ | 133.0679 | 0.00027 | 6.13E-05 | -2E-06 | 1.39E-06 | 0.04 |
| (C_10_H_16_)H^+^ | 137.1320 | 0.00011 | 4.76E-05 | -1.3E-07 | 1.08E-06 | 0.00 |
| (C_8_H_10_O_2_)H^+^ | 139.0750 | 1.11E-05 | 1.31E-05 | 1.05E-06 | 2.98E-07 | 0.21 |
| (C_9_H_18_O)H^+^ | 143.1430 | 4.43E-05 | 8.88E-06 | 1.18E-07 | 2.02E-07 | 0.01 |
| (C_11_H_16_)H^+^ | 149.1320 | 5.89E-05 | 1.19E-05 | -2E-07 | 2.71E-07 | 0.01 |
| (C_10_H_16_O)H^+^ | 153.1270 | 4.7E-05 | 1.1E-05 | -1.5E-07 | 2.51E-07 | 0.01 |
| (C_12_H_25_FO)H^+^ | 205.1962 | -1.2E+28 | 3.68E+28 | 8.03E+25 | 8.26E+26 | 0.00 |


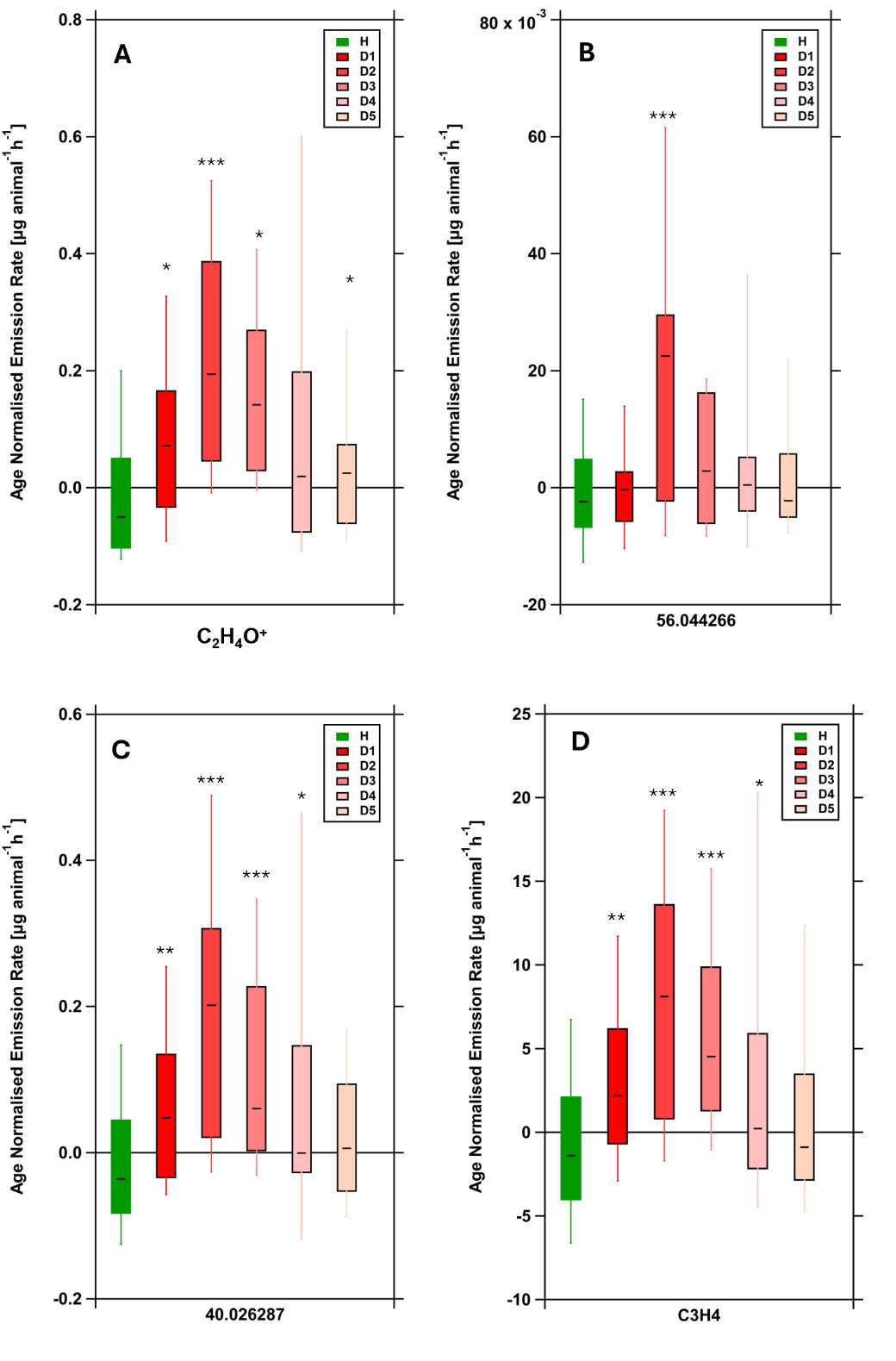


**Figure S1. Box-and-whisker plots of age-normalised breath emission rates of C_2_H_4_O^+^ (A), m/z 50.044 (B), m/z 40.0262 (C) and (C_3_H_4_)H^+^(D) during healthy and diseased periods (progressing from D1 to D5). Asterisks indicate statistical significance: ****p* < 0.001, ***p* < 0.01, **p* < 0.05.**


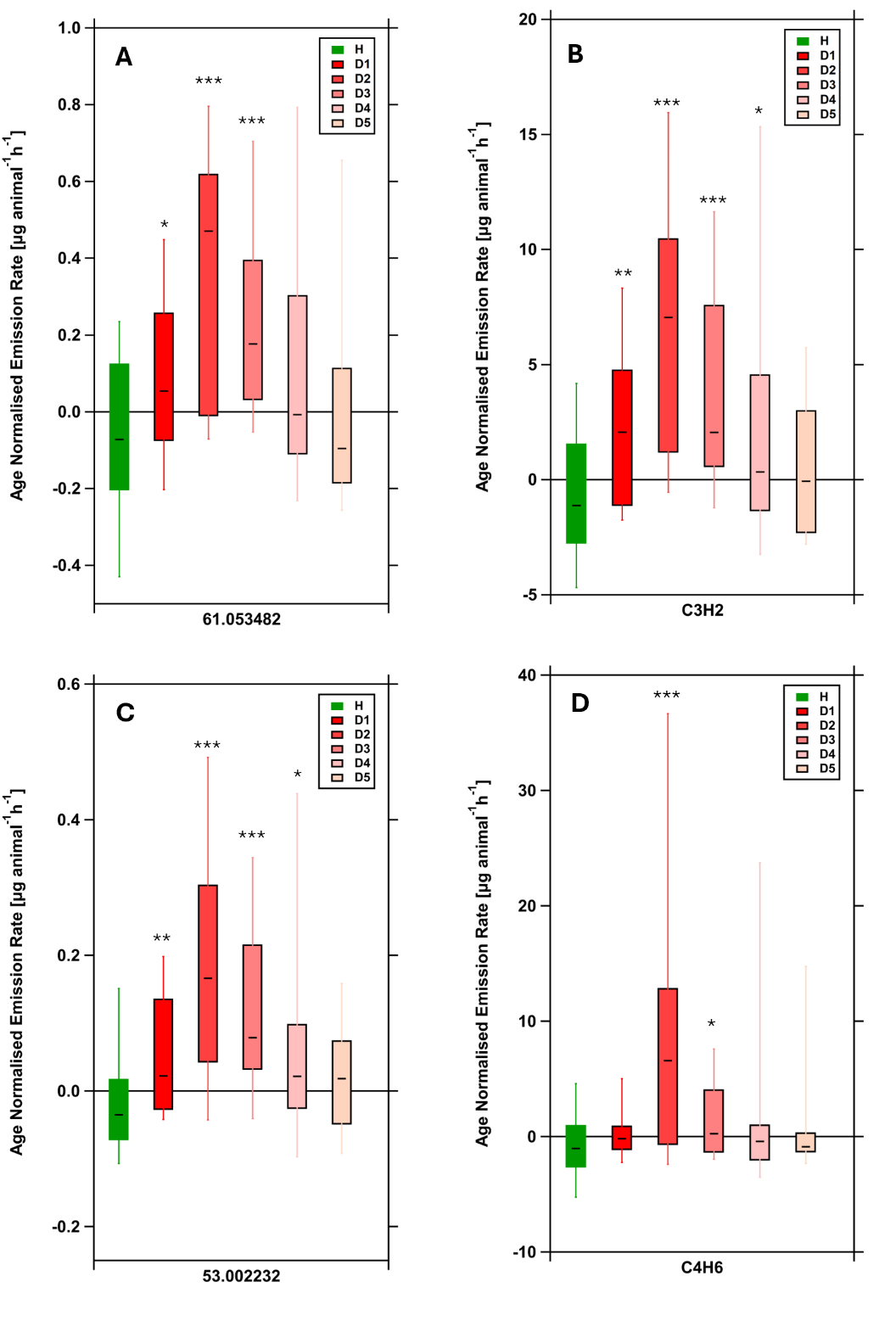


**Figure S2. Box-and-whisker plots of age-normalised breath emission rates of m/z 61.053 (A), (C_3_H_2_)H^+^ (B), m/z 53.002 (C) and (C_4_H_6_)H^+^ (D) during healthy and diseased periods (progressing from D1 to D5). Asterisks indicate statistical significance: ****p* < 0.001, ***p* < 0.01, **p* < 0.05.**


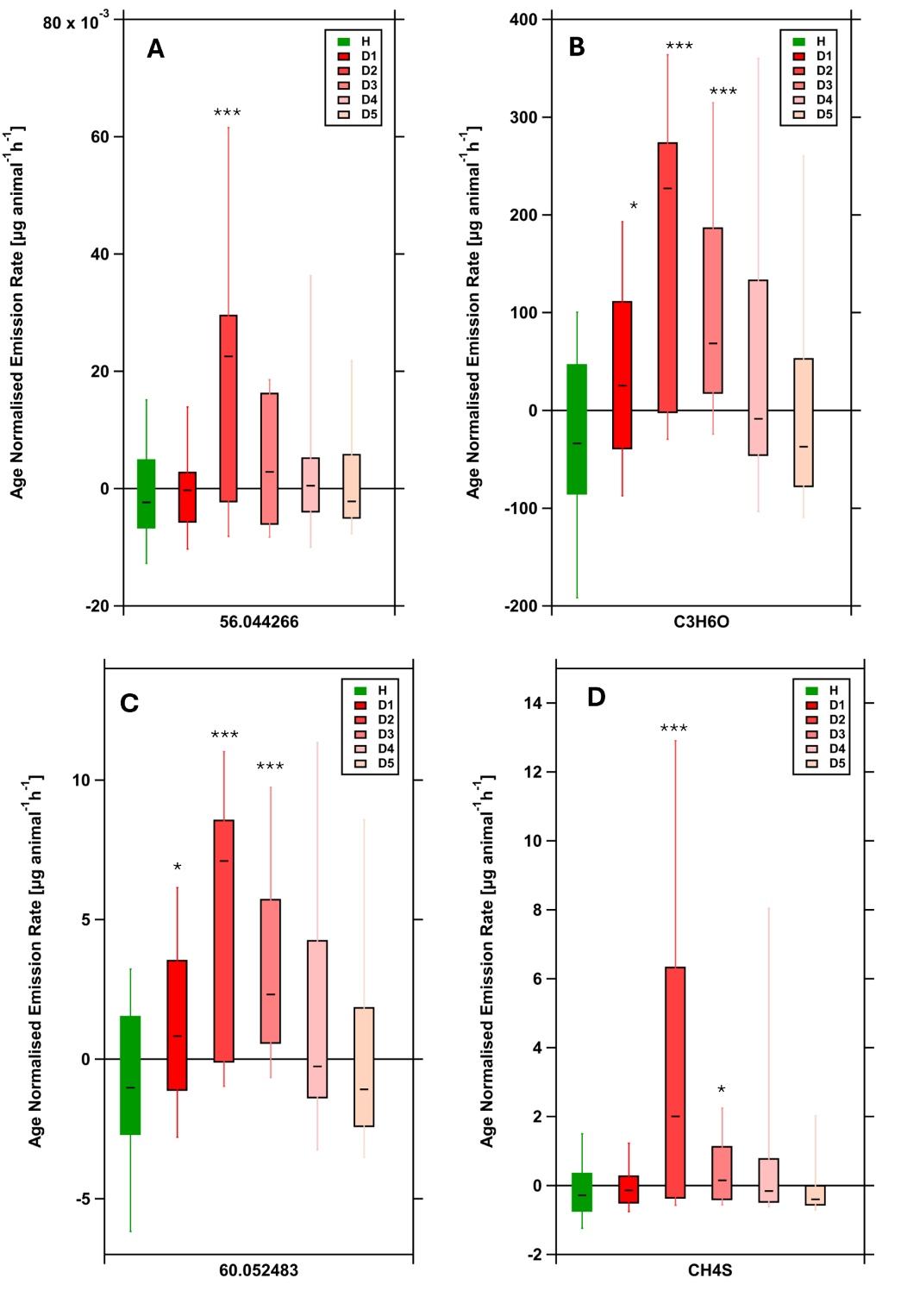


**Figure S3. Box-and-whisker plots of age-normalised breath emission rates of m/z 56.044 (A), (C_3_H_6_O)H^+^ (B), m/z 60.052 (C) and CH_4_S^+^ (D) during healthy and diseased periods (progressing from D1 to D5). Asterisks indicate statistical significance: ****p* < 0.001, ***p* < 0.01, **p* < 0.05.**


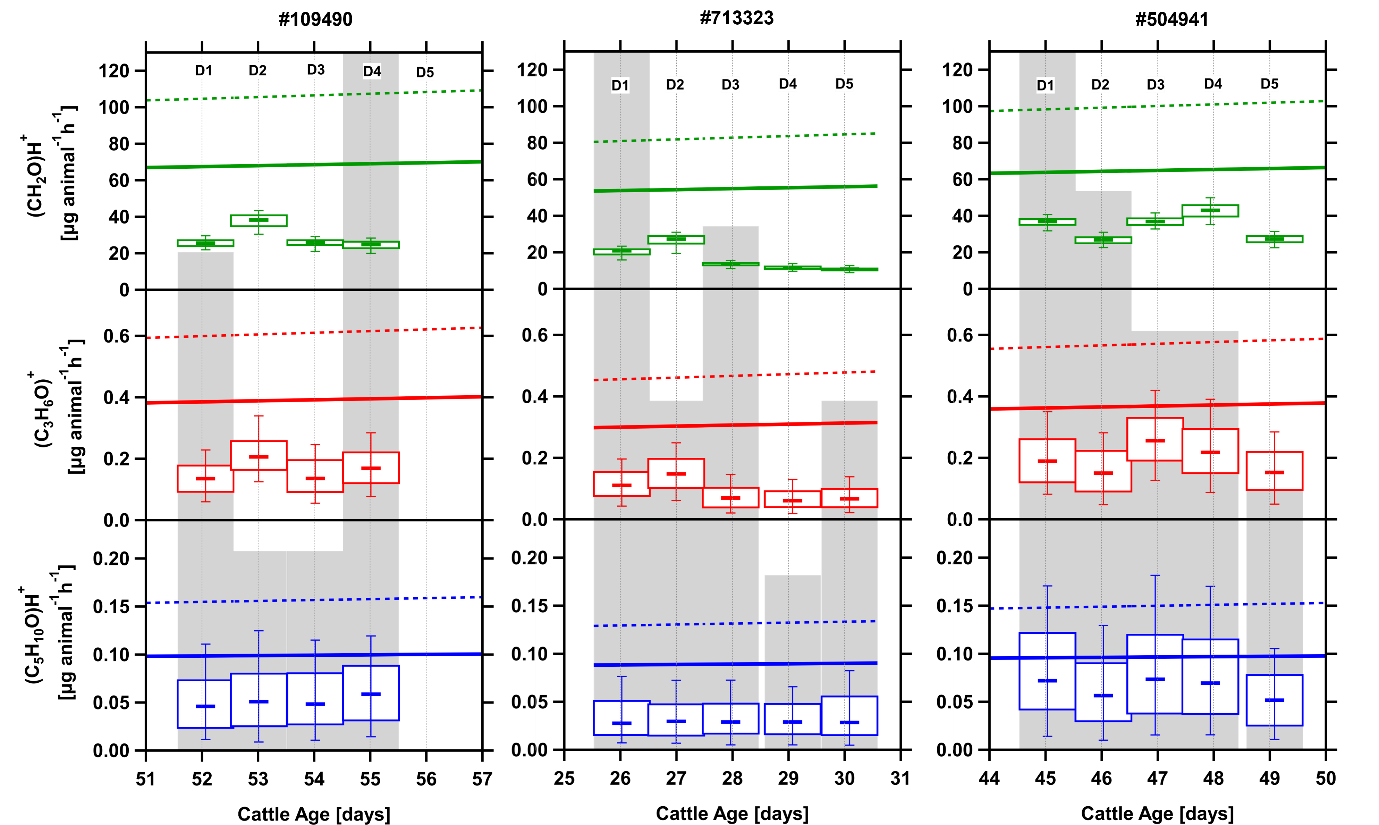


**Figure S4. Time series of emission rates for formaldehyde ((CH₂O)H⁺), acetone/propanal (C₃H₆O⁺), and ((C₅H₁₀O⁺)H^+^) ions from cattle over five days following the onset of clinically identifiable disease. Although scored as diseased, these animals did not receive antibiotic treatment. Solid lines represent growth response curves from the healthy cohort; dashed lines indicate the 95^th^ percentile. Shaded areas denote relative health scores.**

**
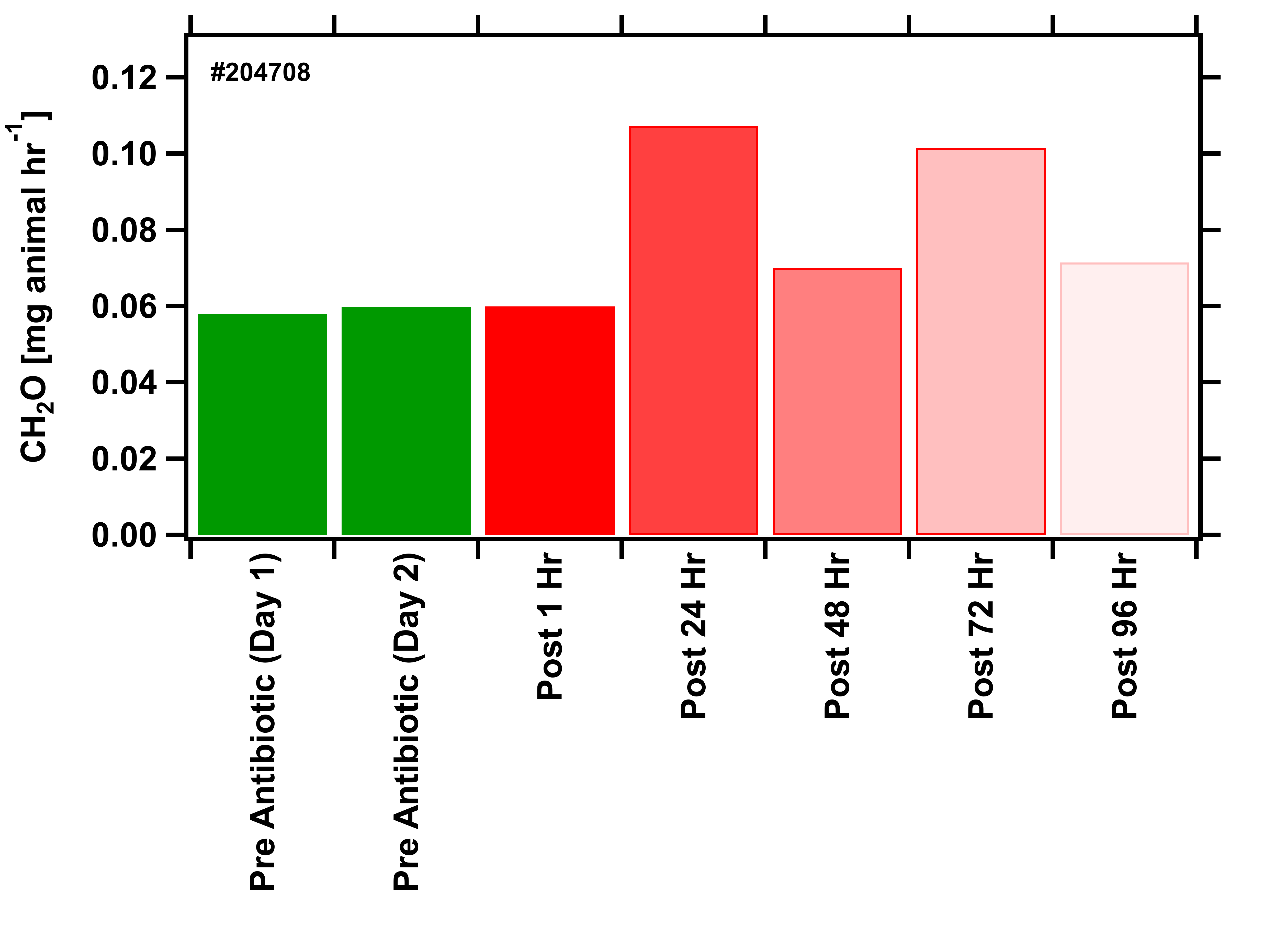
**

**Figure S5. Emission rates of formaldehyde (CH₂O) measured from healthy calf #204708 two days prior to antibiotic treatment (green), and at 1, 24, 48, 72, and 96 hours following injection with Alamycin LA 300.**

**
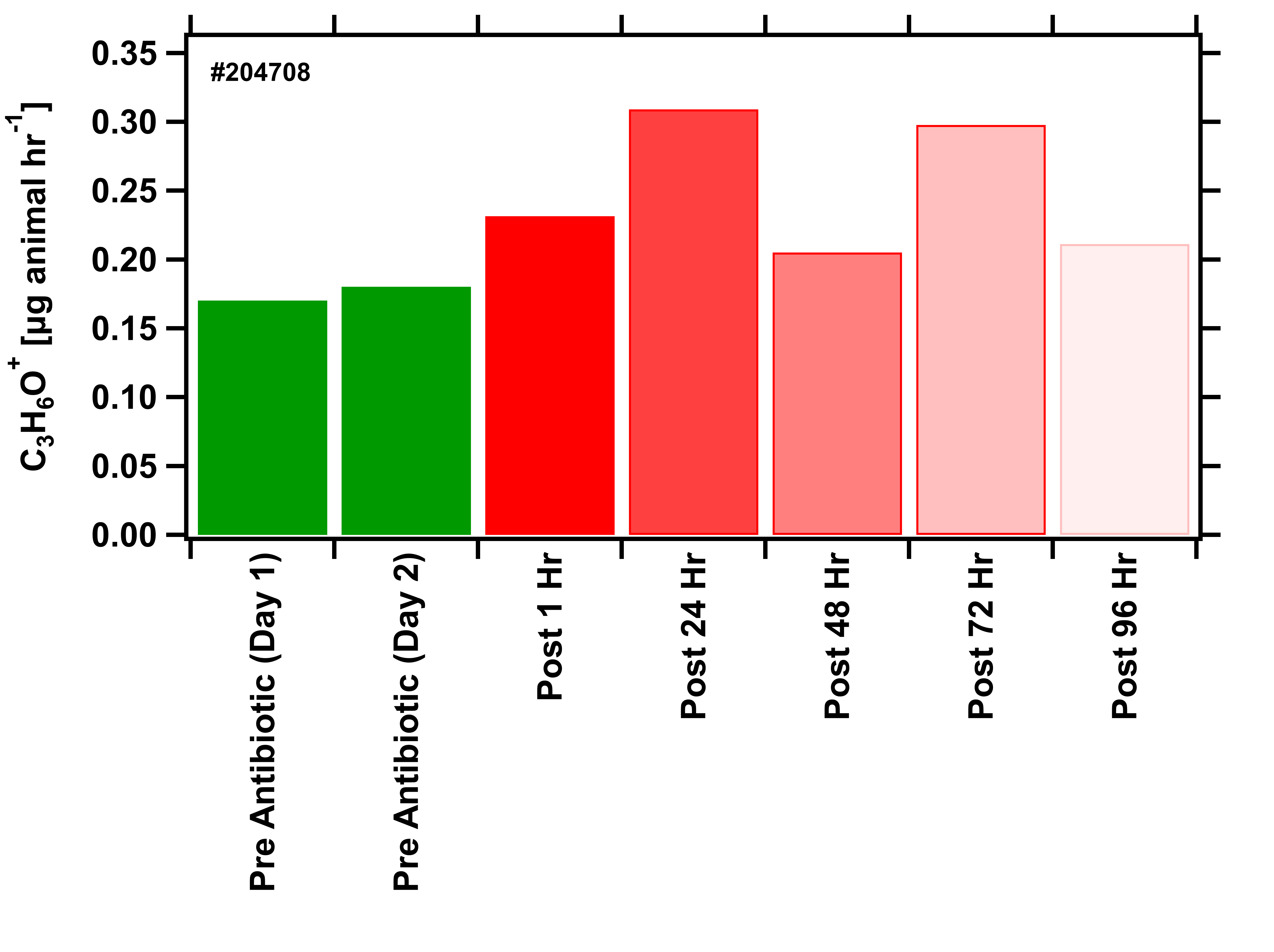
**

**Figure S6. Emission rates of acetone/propanal (C_3_H_6_O^+^) measured from healthy calf #204708 two days prior to antibiotic treatment (green), and at 1, 24, 48, 72, and 96 hours following injection with Alamycin LA 300.**

**
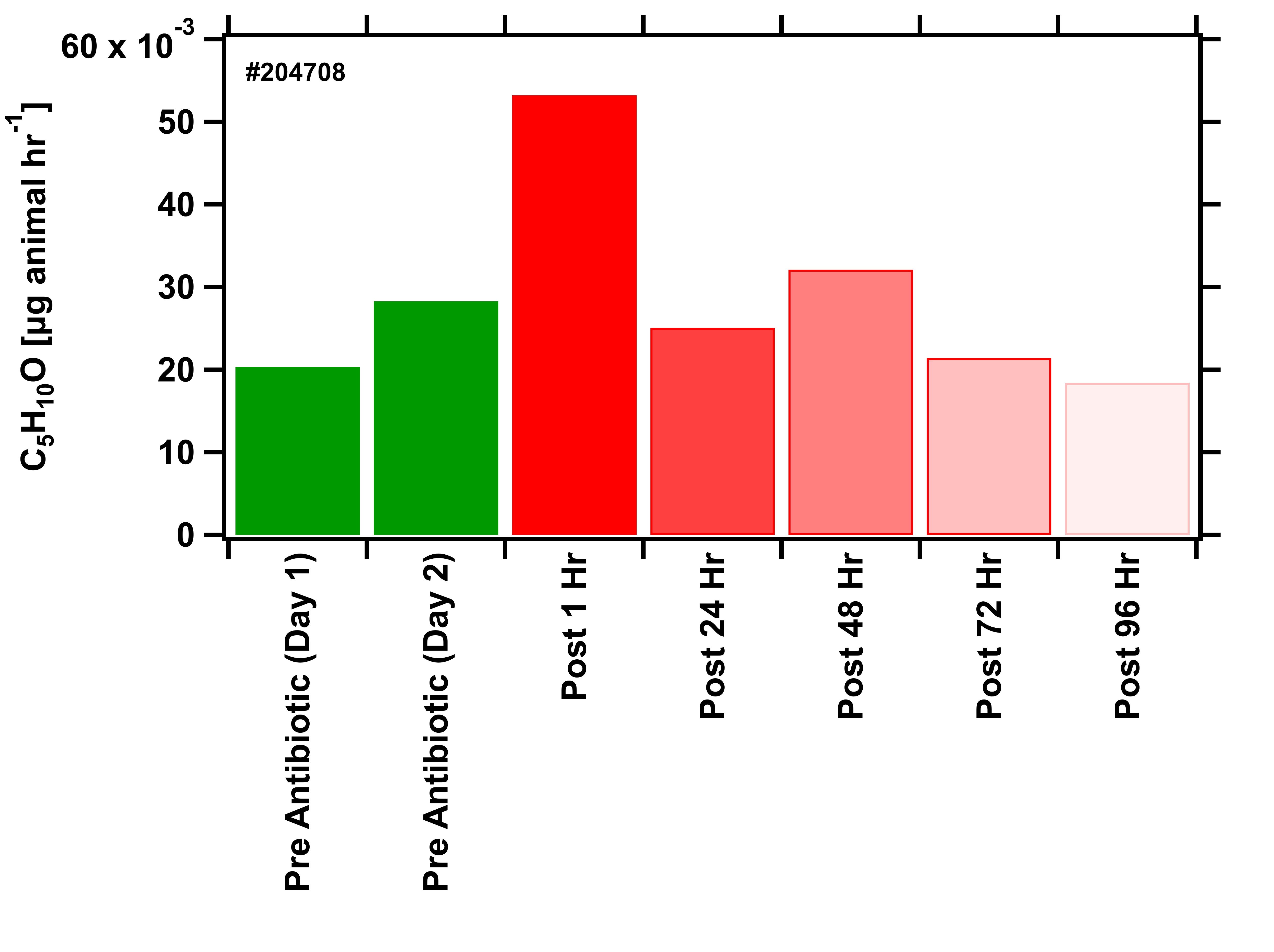
**

**Figure S7. Emission rates of C_5_H_10_O measured from healthy calf #204708 two days prior to antibiotic treatment (green), and at 1, 24, 48, 72, and 96 hours following injection with Alamycin LA 300.**

**
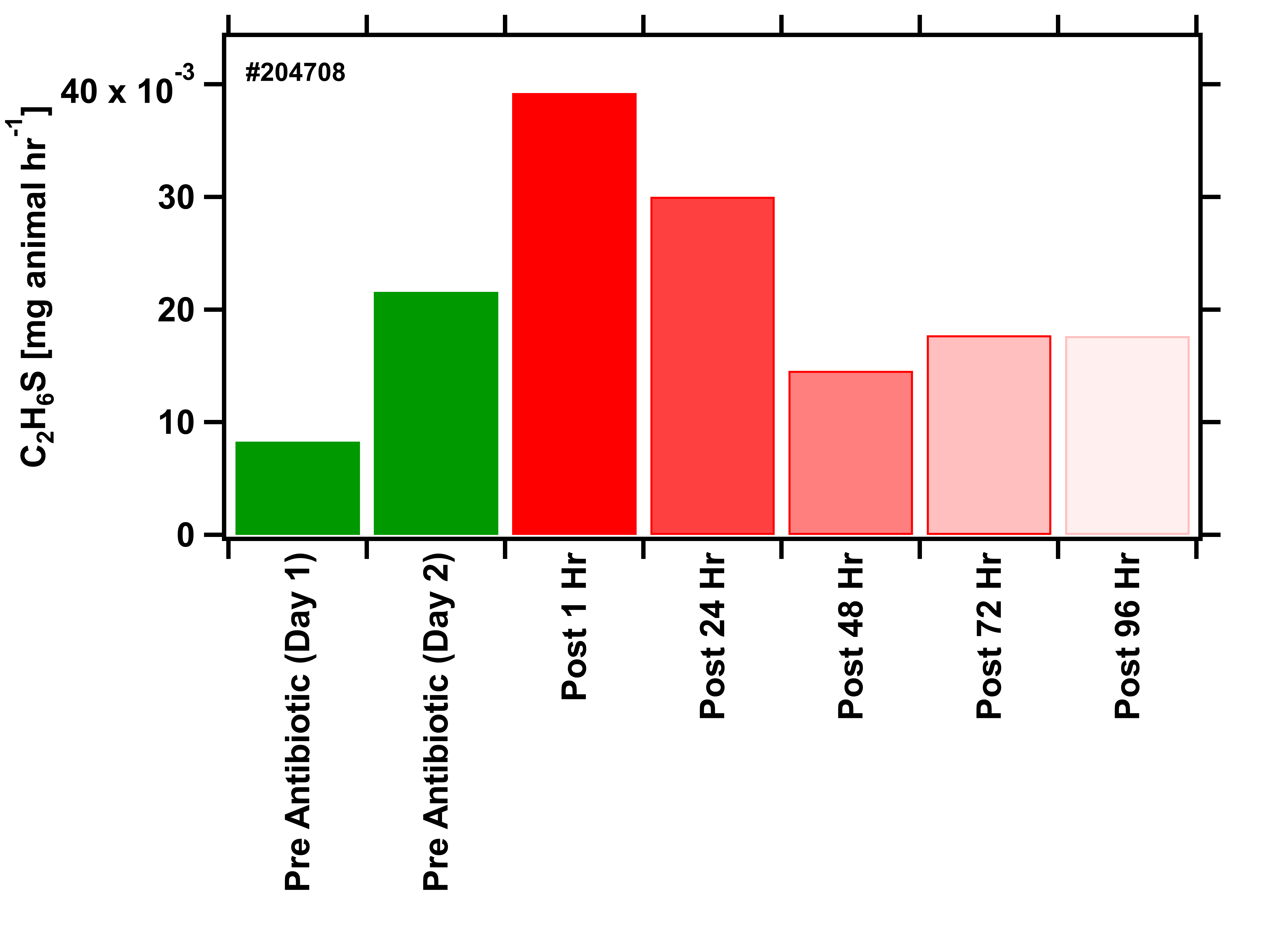
**

**Figure S8. Emission rates of dimethyl sulphide (C_2_H_6_S) measured from healthy calf #204708 two days prior to antibiotic treatment (green), and at 1, 24, 48, 72, and 96 hours following injection with Alamycin LA 300.**

**
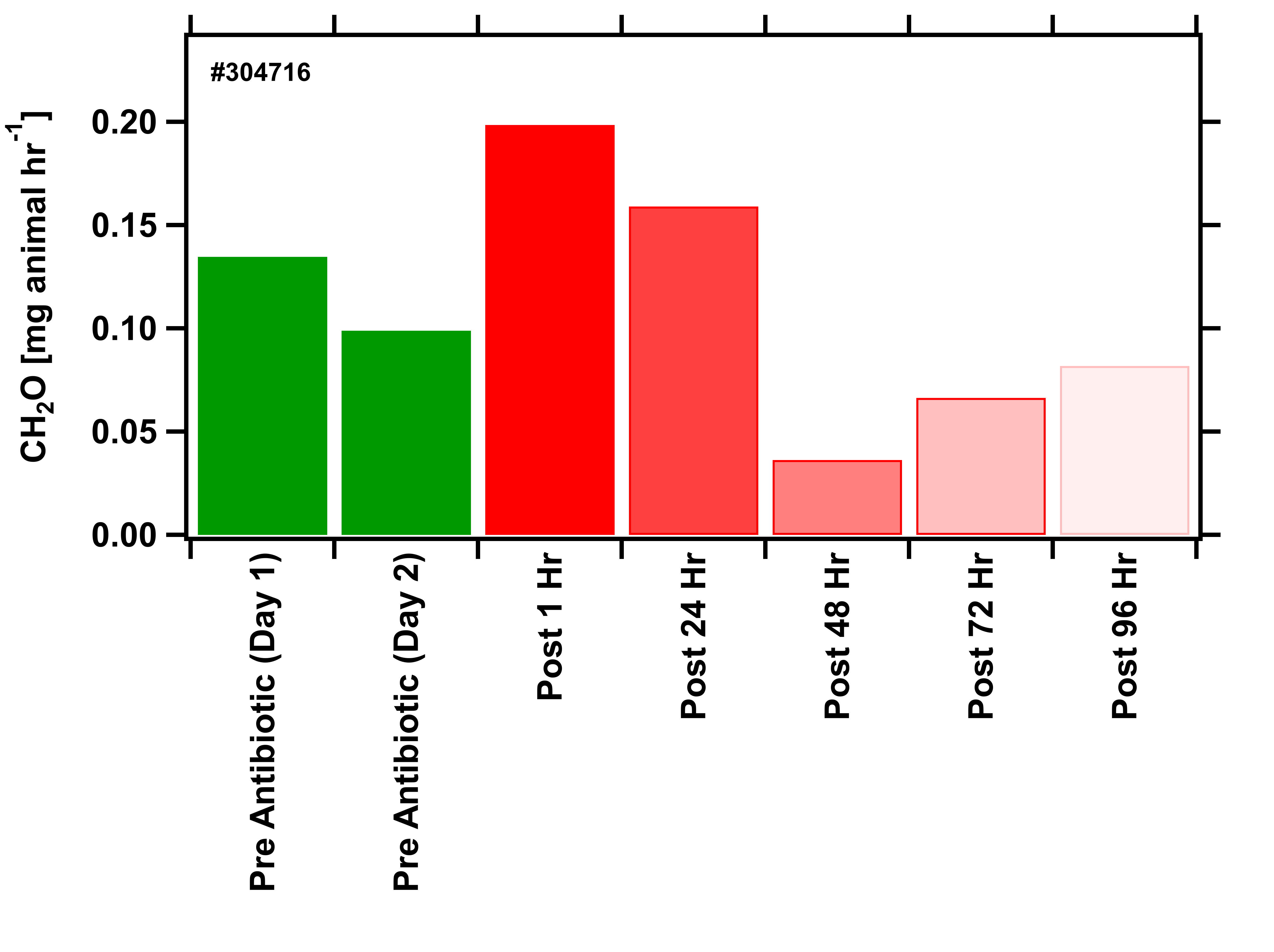
**

**Figure S9. Emission rates of formaldehyde (CH₂O) measured from healthy calf #304716 two days prior to antibiotic treatment (green), and at 1, 24, 48, 72, and 96 hours following injection with Alamycin LA 300.**

**
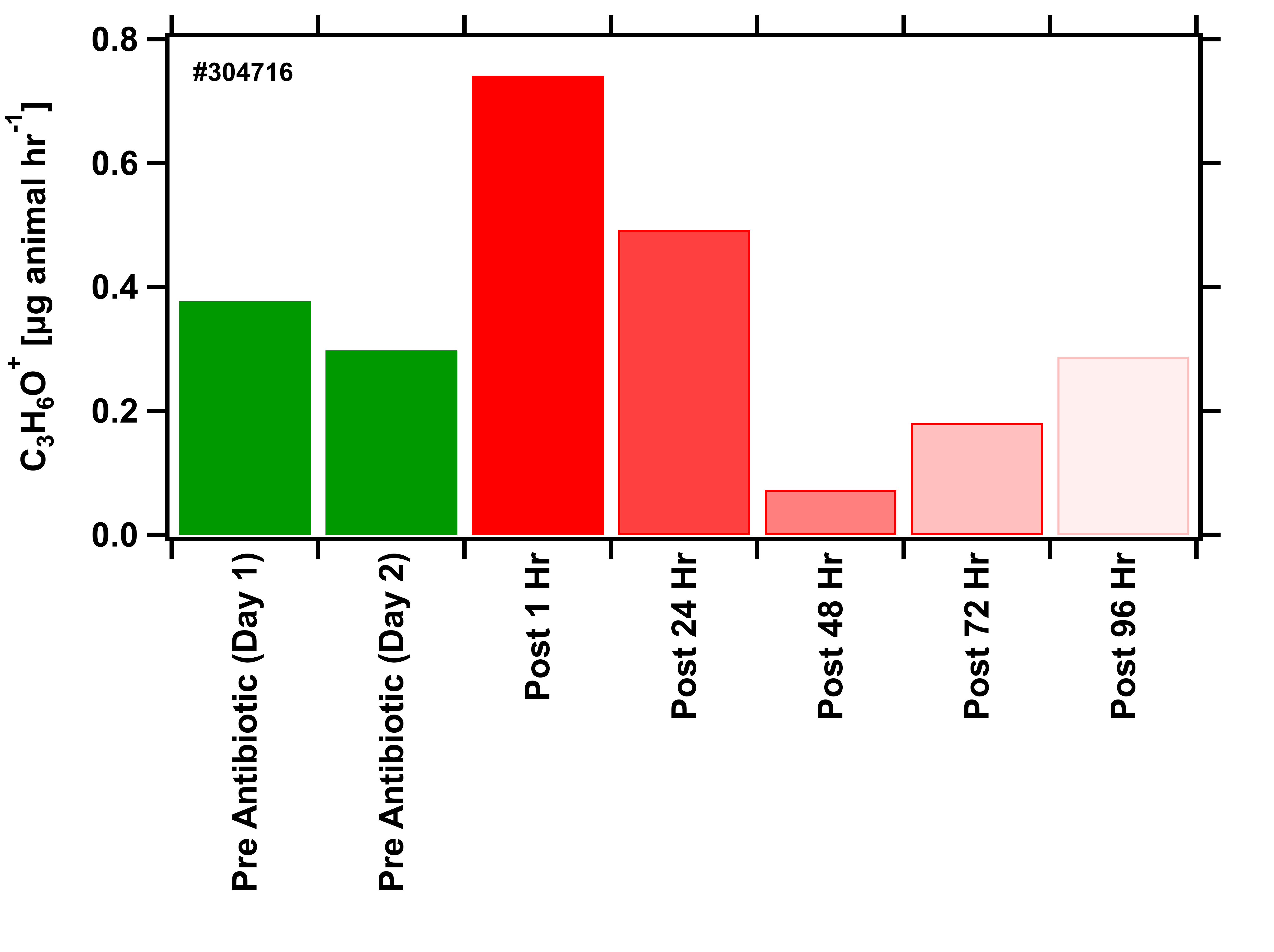
**

**Figure S10. Emission rates of acetone/propanal (C_3_H_6_O^+^) measured from healthy calf #304716 two days prior to antibiotic treatment (green), and at 1, 24, 48, 72, and 96 hours following injection with Alamycin LA 300.**

**
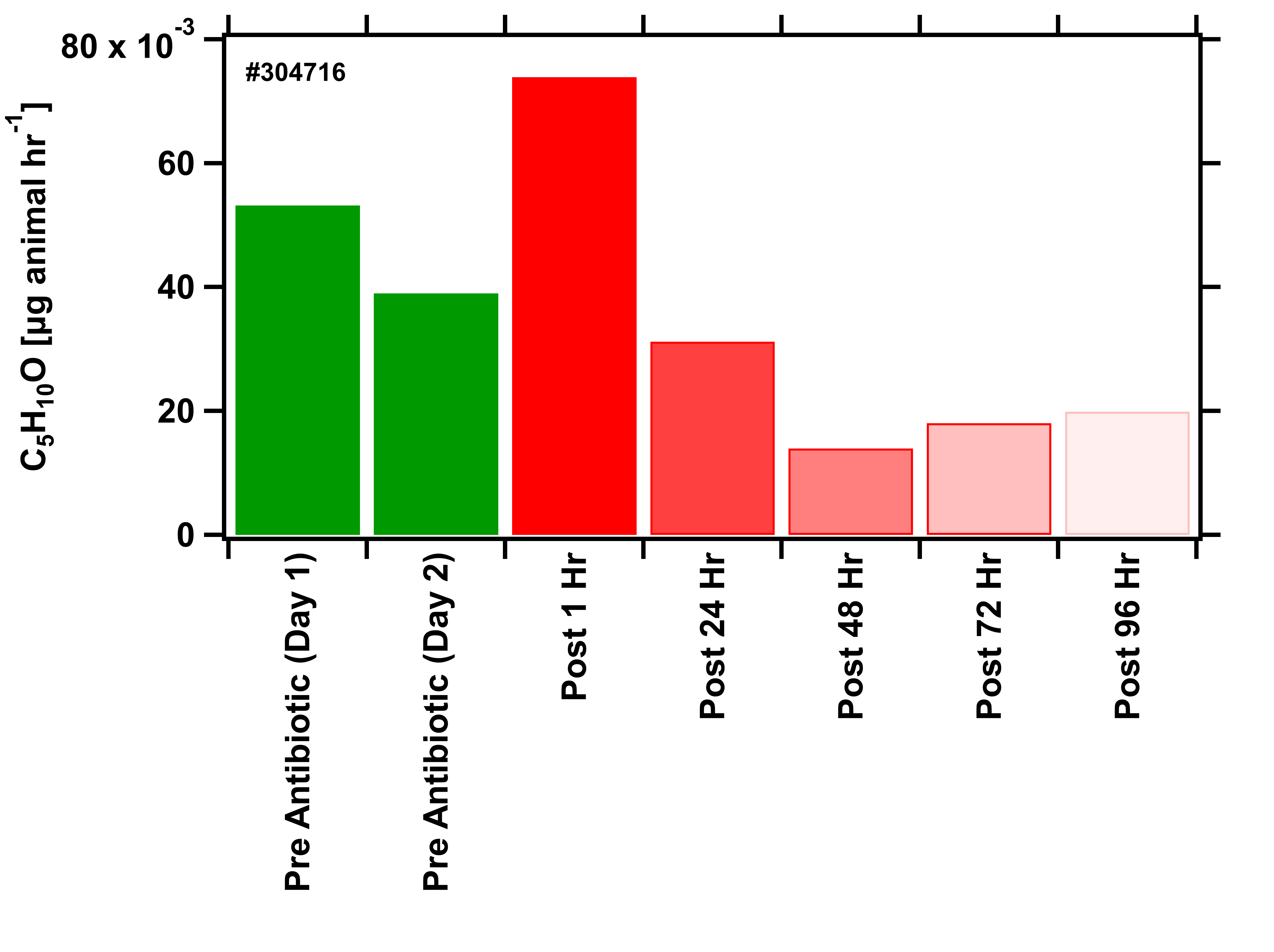
**

**Figure S11. Emission rates of C_5_H_10_O measured from healthy calf #304716 two days prior to antibiotic treatment (green), and at 1, 24, 48, 72, and 96 hours following injection with Alamycin LA 300.**

**
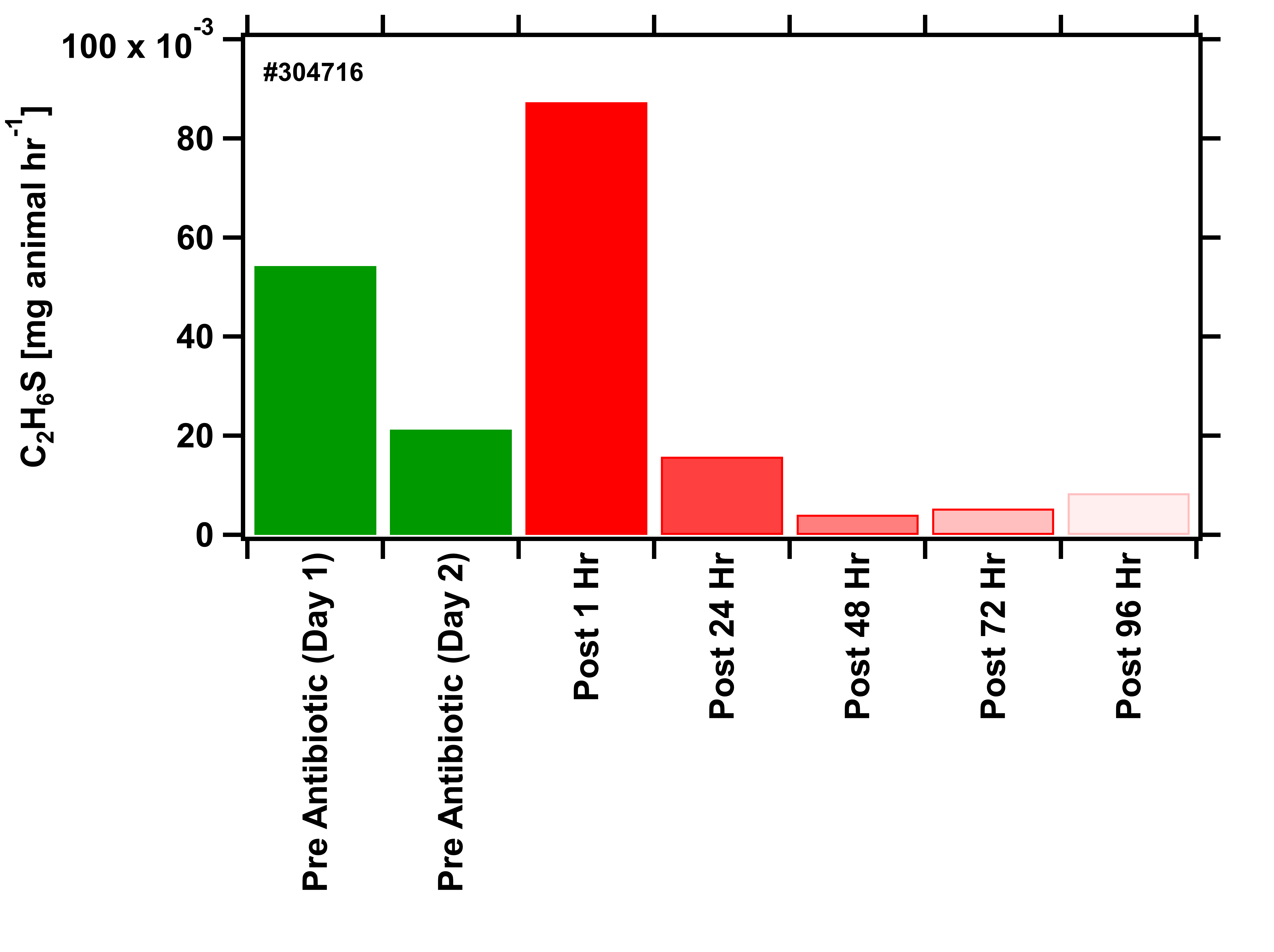
**

**Figure S12. Emission rates of dimethyl sulphide (C_2_H_6_S) measured from healthy calf #304716 two days prior to antibiotic treatment (green), and at 1, 24, 48, 72, and 96 hours following injection with Alamycin LA 300.**

**
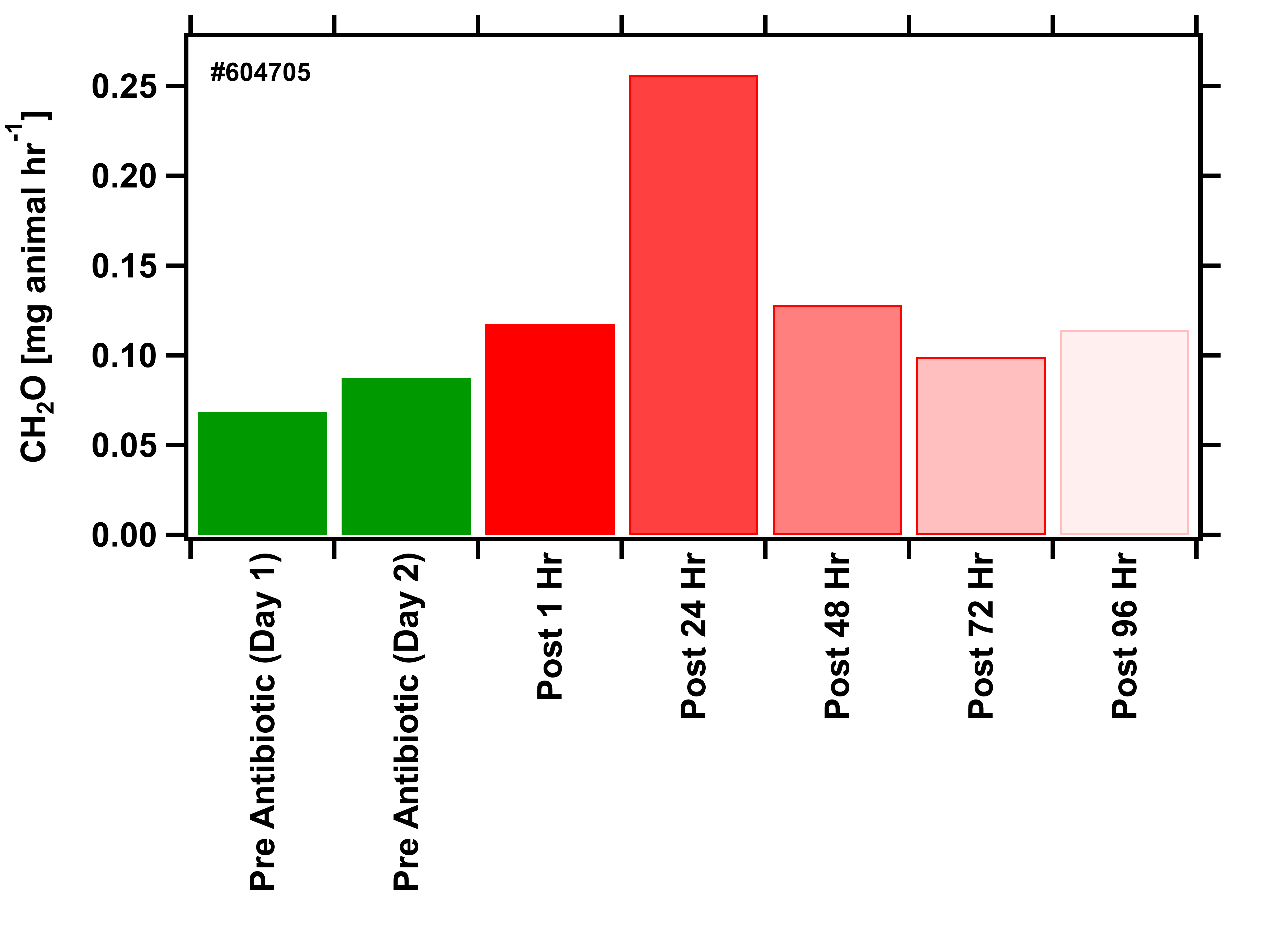
**

**Figure S13. Emission rates of formaldehyde (CH₂O) measured from healthy calf #604705 two days prior to antibiotic treatment (green), and at 1, 24, 48, 72, and 96 hours following injection with Alamycin LA 300.**

**
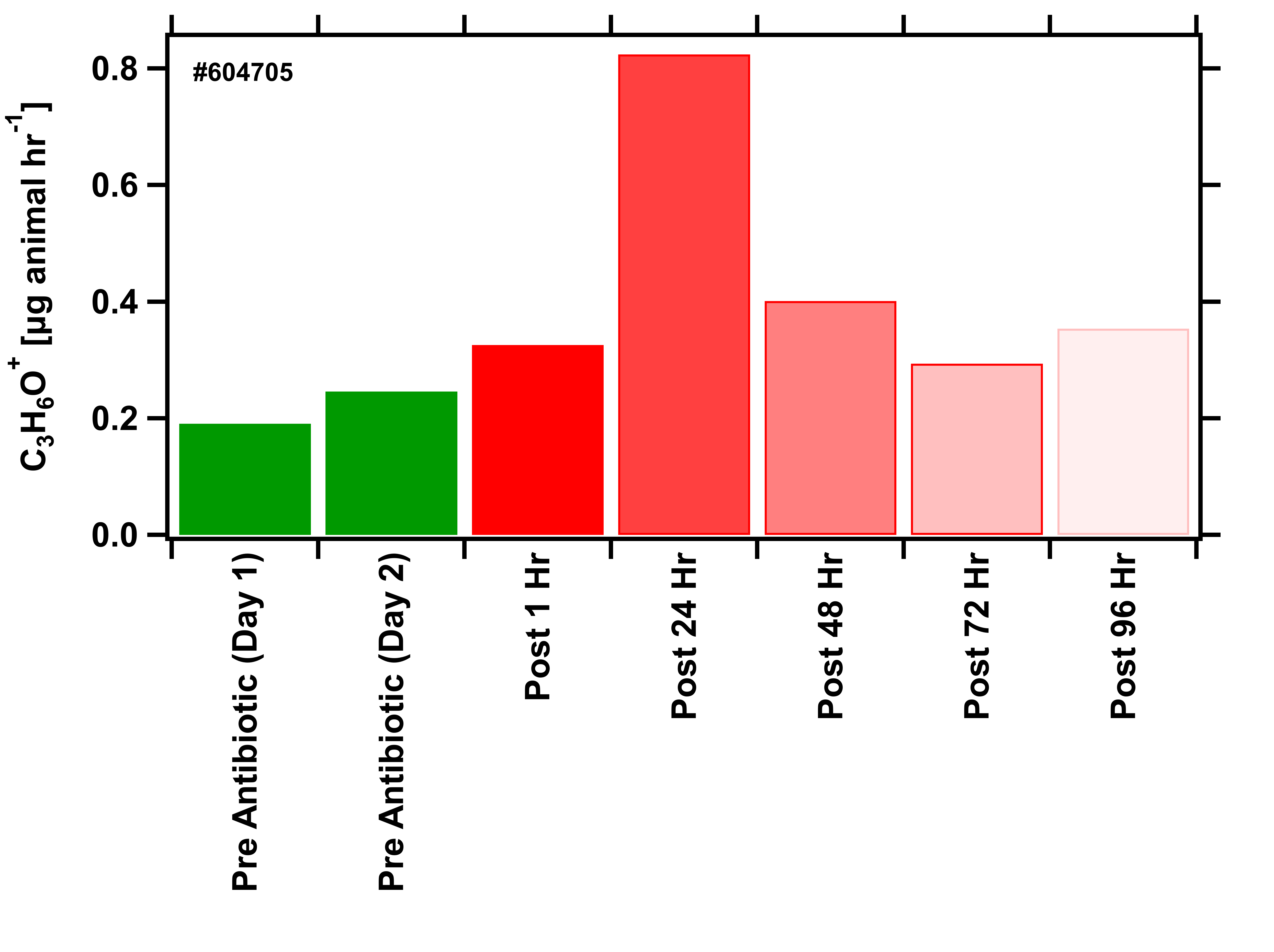
**

**Figure S14. Emission rates of acetone/propanal (C_3_H_6_O^+^) measured from healthy calf #604705 two days prior to antibiotic treatment (green), and at 1, 24, 48, 72, and 96 hours following injection with Alamycin LA 300.**

**
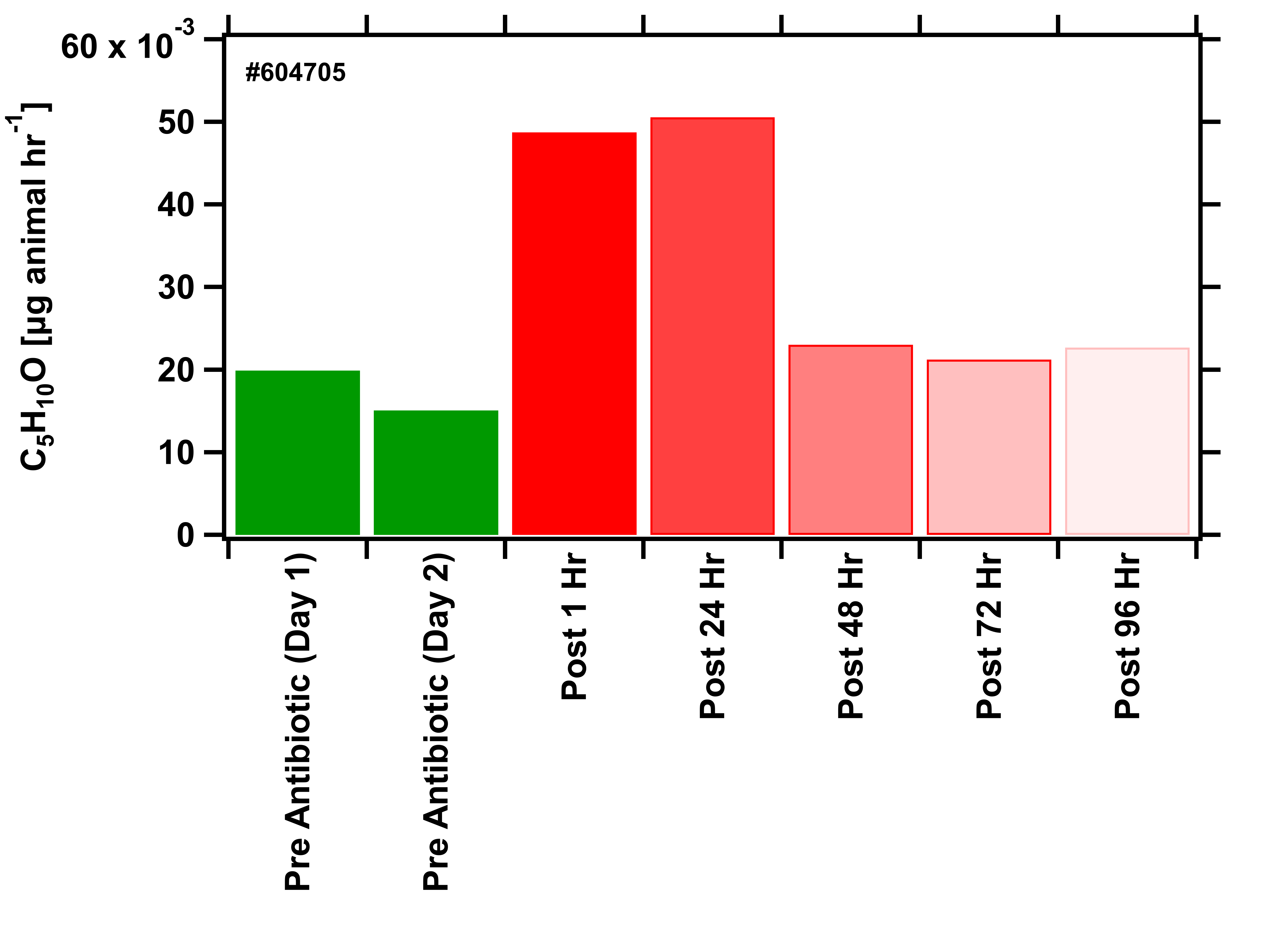
**

**Figure S15. Emission rates of C_5_H_10_O measured from healthy calf #604705 two days prior to antibiotic treatment (green), and at 1, 24, 48, 72, and 96 hours following injection with Alamycin LA 300.**

**
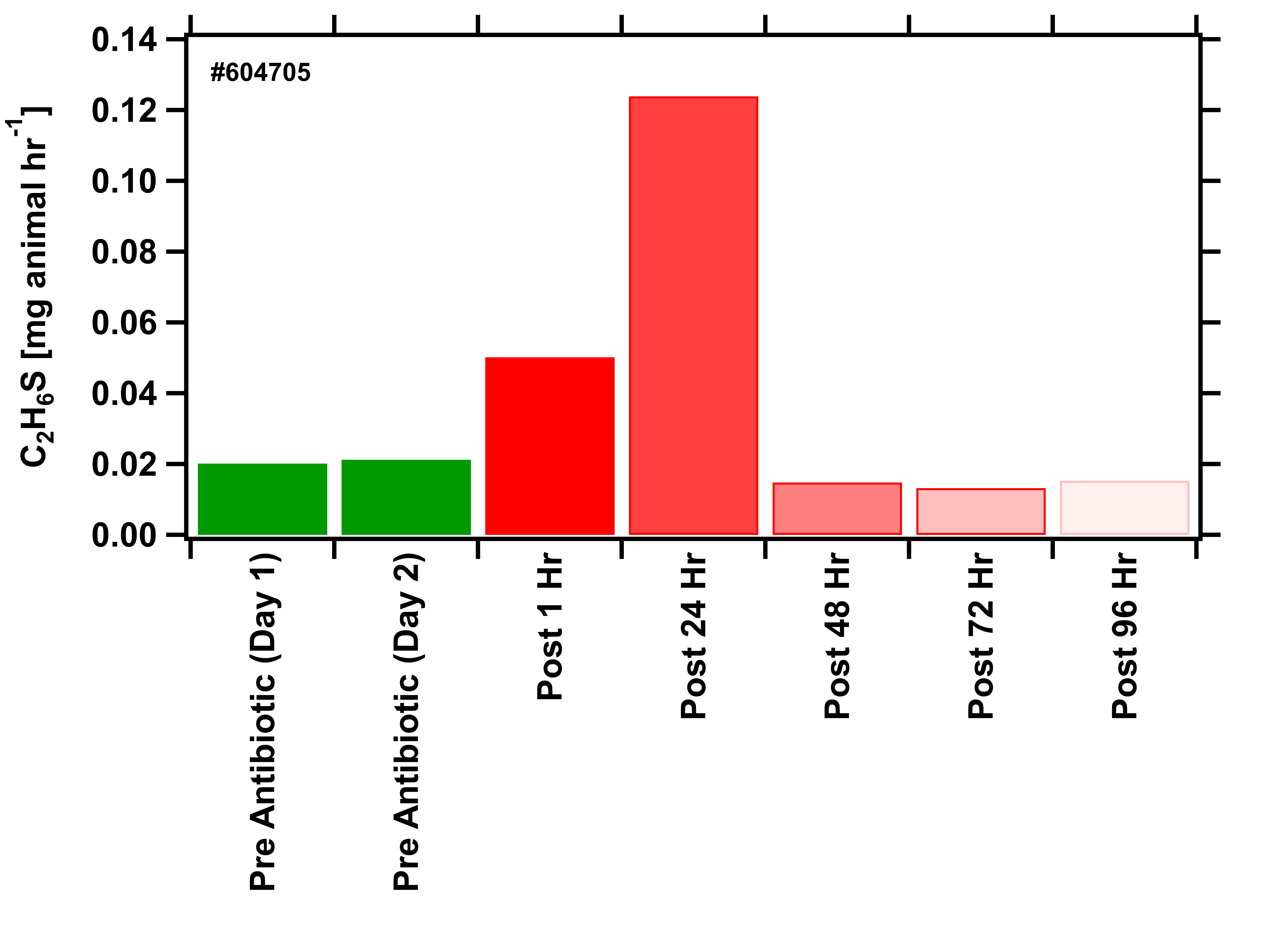
**

**Figure S16. Emission rates of dimethyl sulphide (C_2_H_6_S) measured from healthy calf #604705 two days prior to antibiotic treatment (green), and at 1, 24, 48, 72, and 96 hours following injection with Alamycin LA 300.**

**
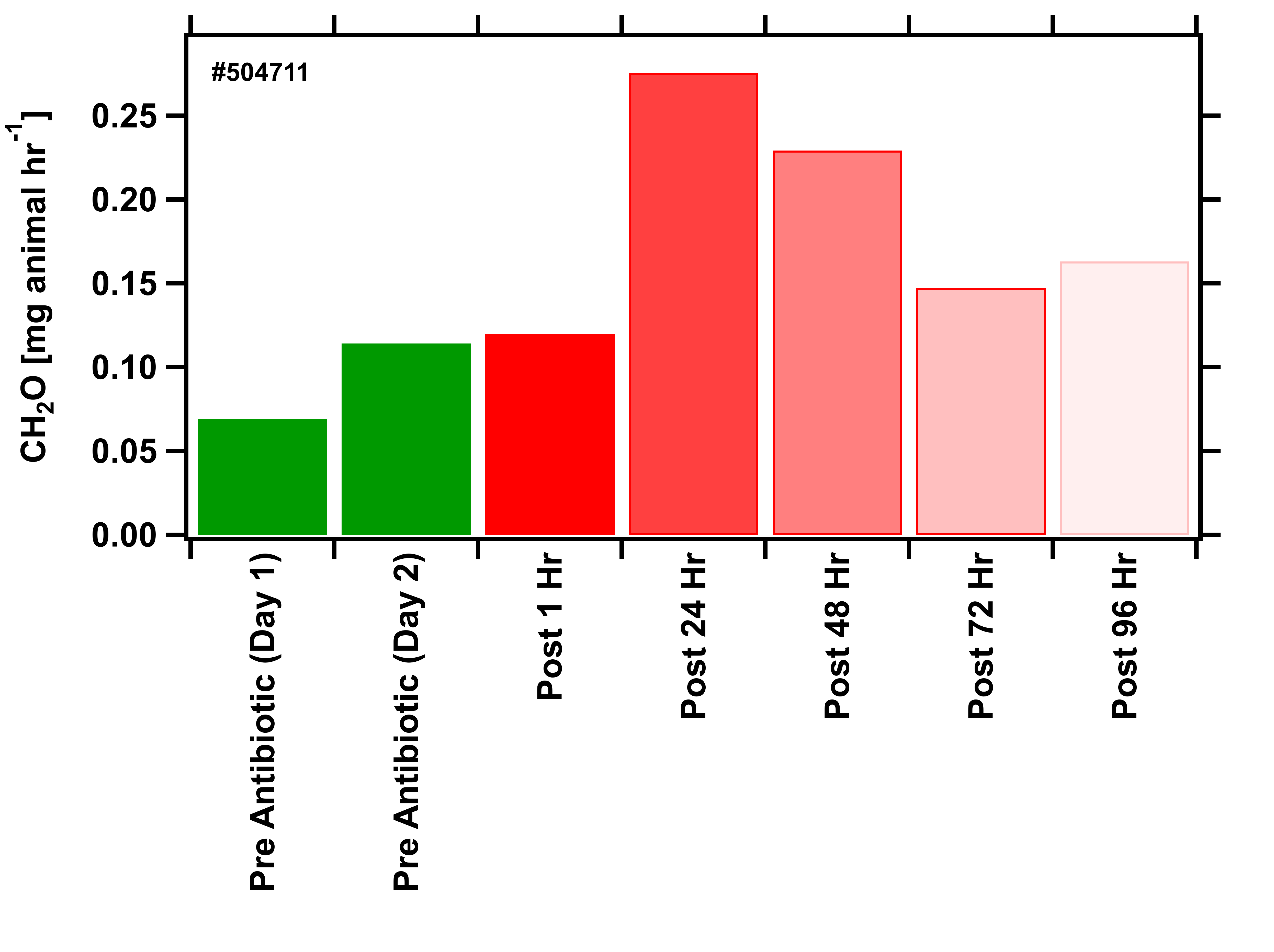
**

**Figure S17. Emission rates of formaldehyde (CH₂O) measured from healthy calf #504711 two days prior to antibiotic treatment (green), and at 1, 24, 48, 72, and 96 hours following injection with Alamycin LA 300.**

**
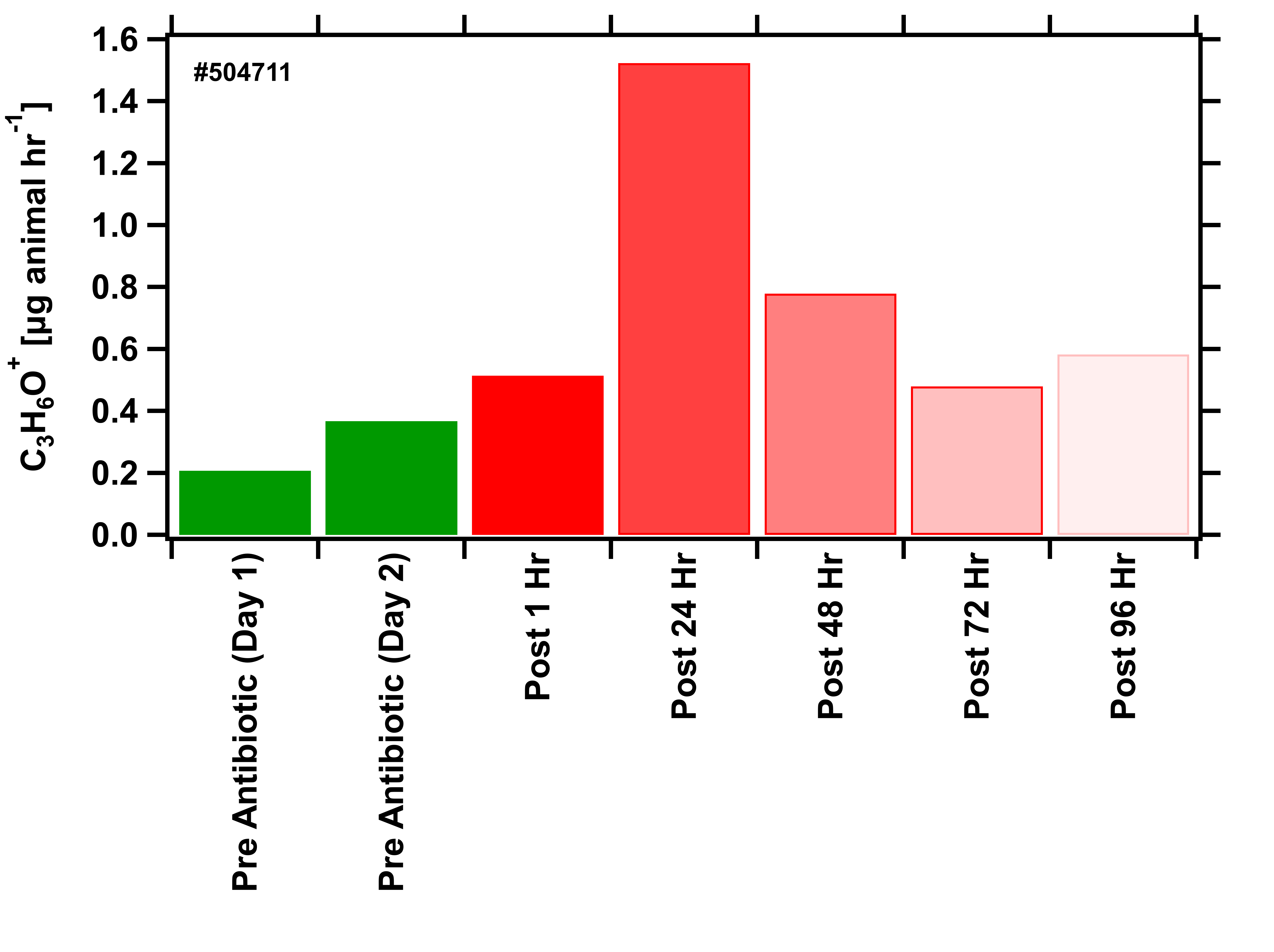
**

**Figure S18. Emission rates of acetone/propanal (C_3_H_6_O^+^) measured from healthy calf #504711 two days prior to antibiotic treatment (green), and at 1, 24, 48, 72, and 96 hours following injection with Alamycin LA 300.**

**
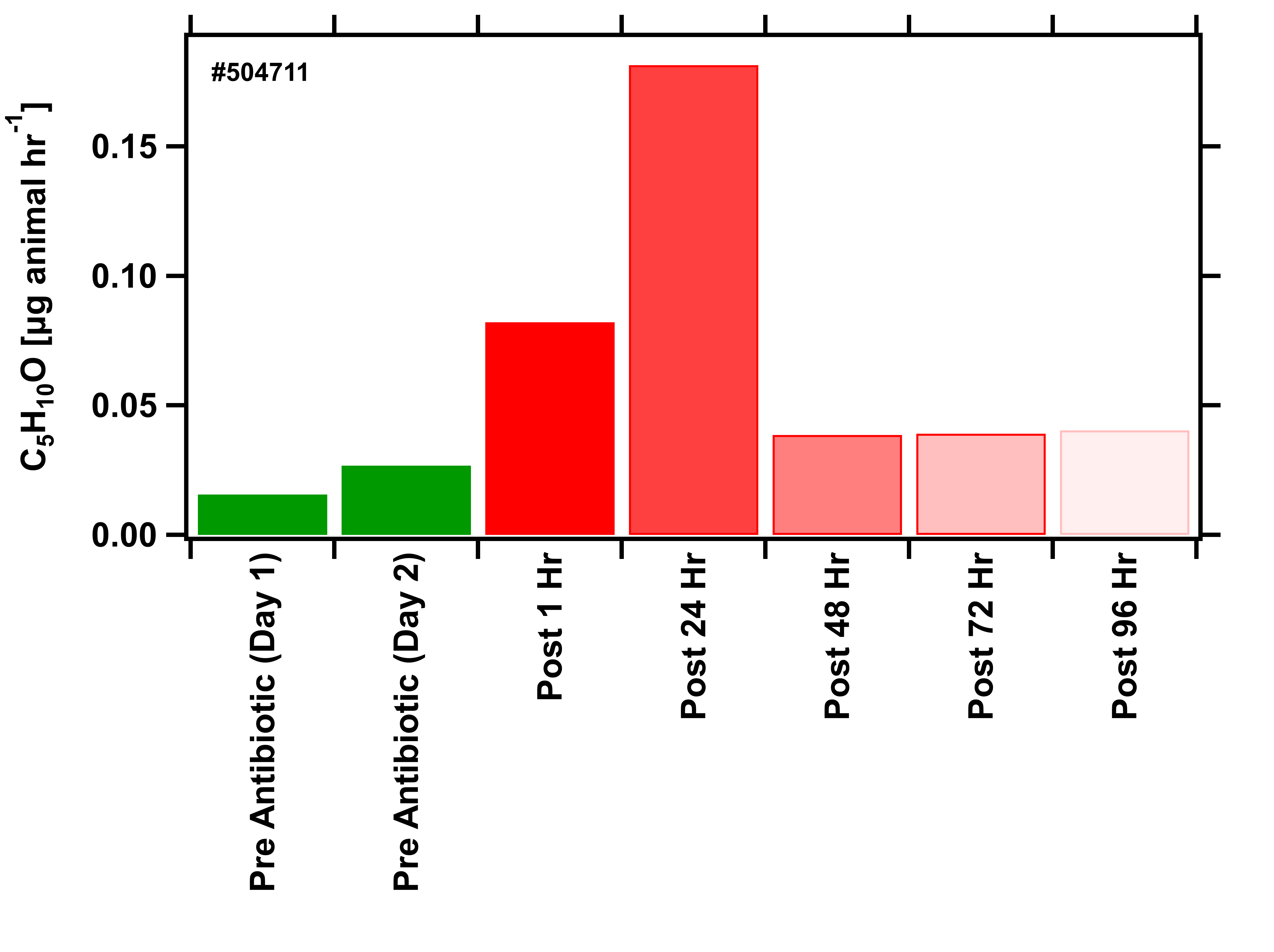
**

**Figure S19. Emission rates of C_5_H_10_O measured from healthy calf #504711 two days prior to antibiotic treatment (green), and at 1, 24, 48, 72, and 96 hours following injection with Alamycin LA 300.**

**
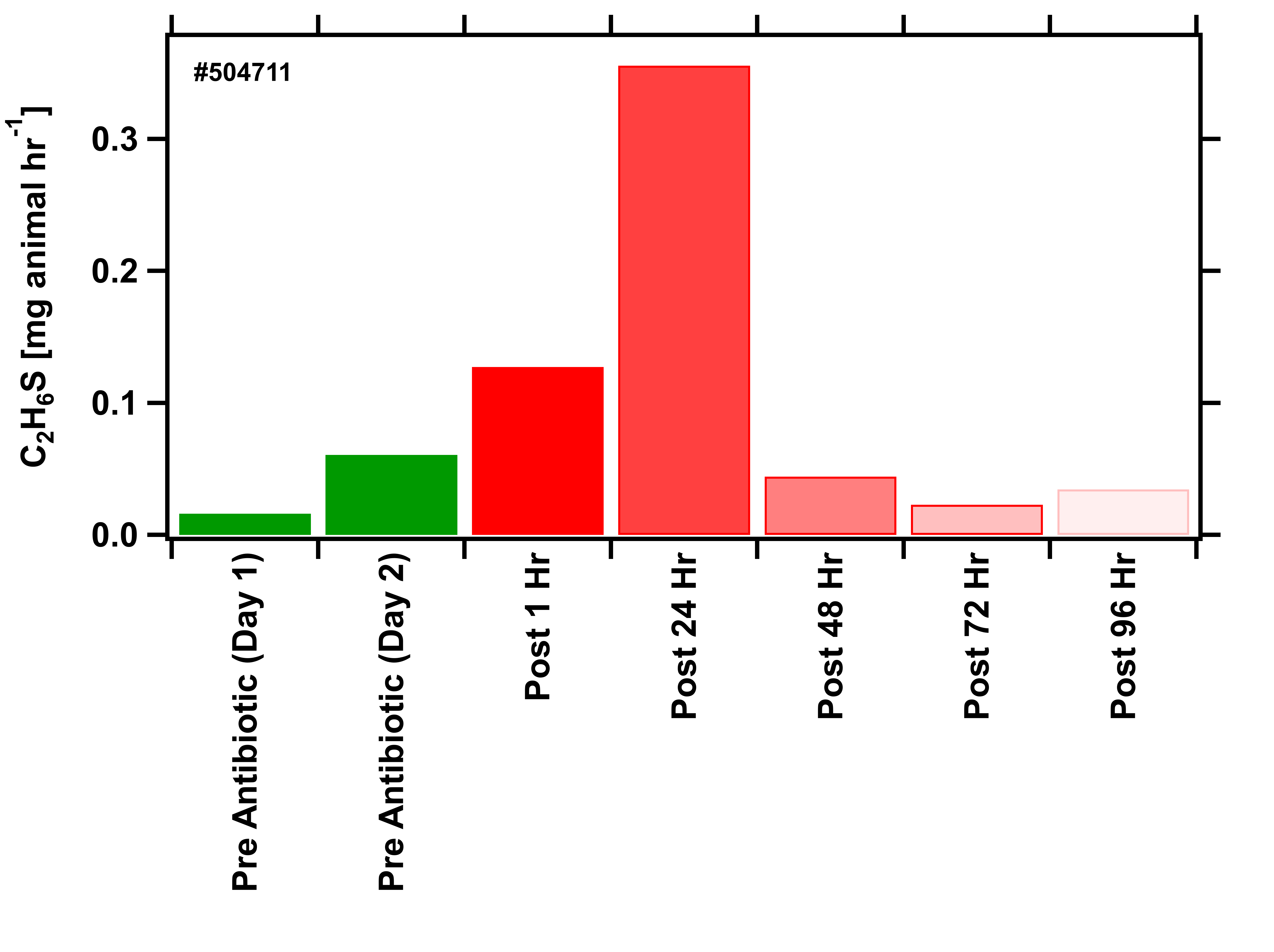
**

**Figure S20. Emission rates of dimethyl sulphide (C_2_H_6_S) measured from healthy calf #504711 two days prior to antibiotic treatment (green), and at 1, 24, 48, 72, and 96 hours following injection with Alamycin LA 300.**

**
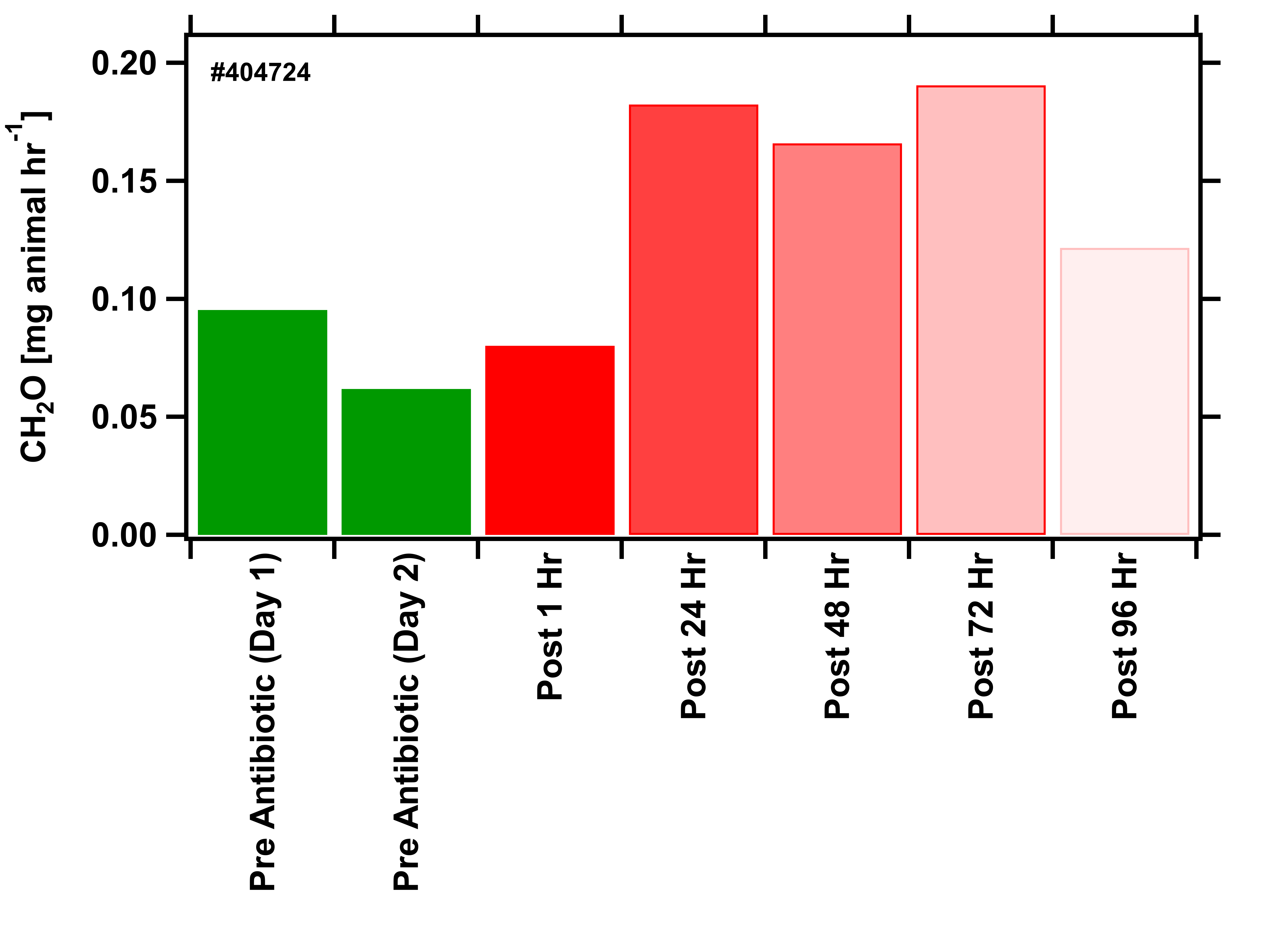
**

**Figure S21. Emission rates of formaldehyde (CH₂O) measured from healthy calf #404724 two days prior to antibiotic treatment (green), and at 1, 24, 48, 72, and 96 hours following injection with Alamycin LA 300.**

**
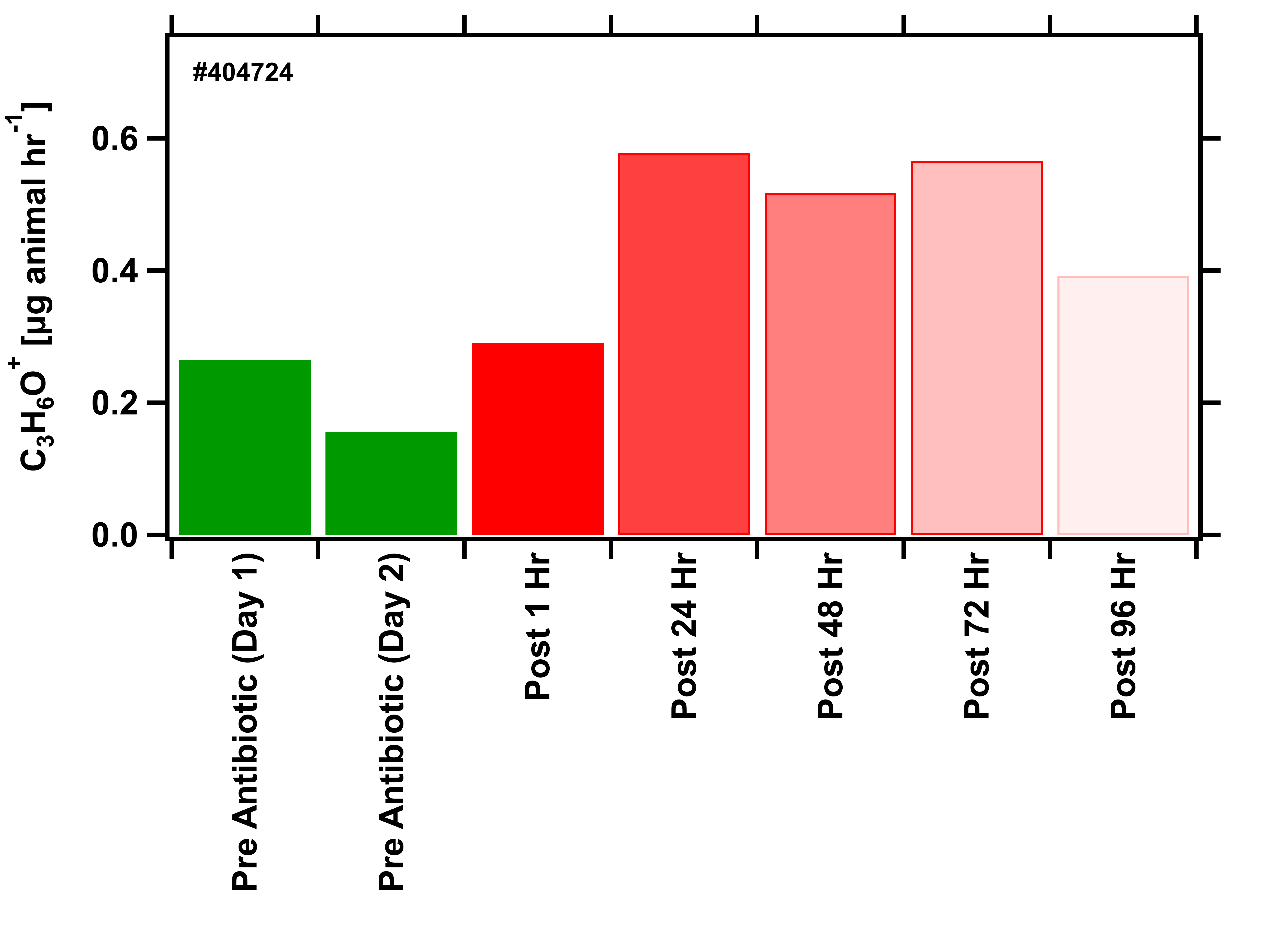
**

**Figure S22. Emission rates of acetone/propanal (C_3_H_6_O^+^) measured from healthy calf #404724 two days prior to antibiotic treatment (green), and at 1, 24, 48, 72, and 96 hours following injection with Alamycin LA 300.**

**
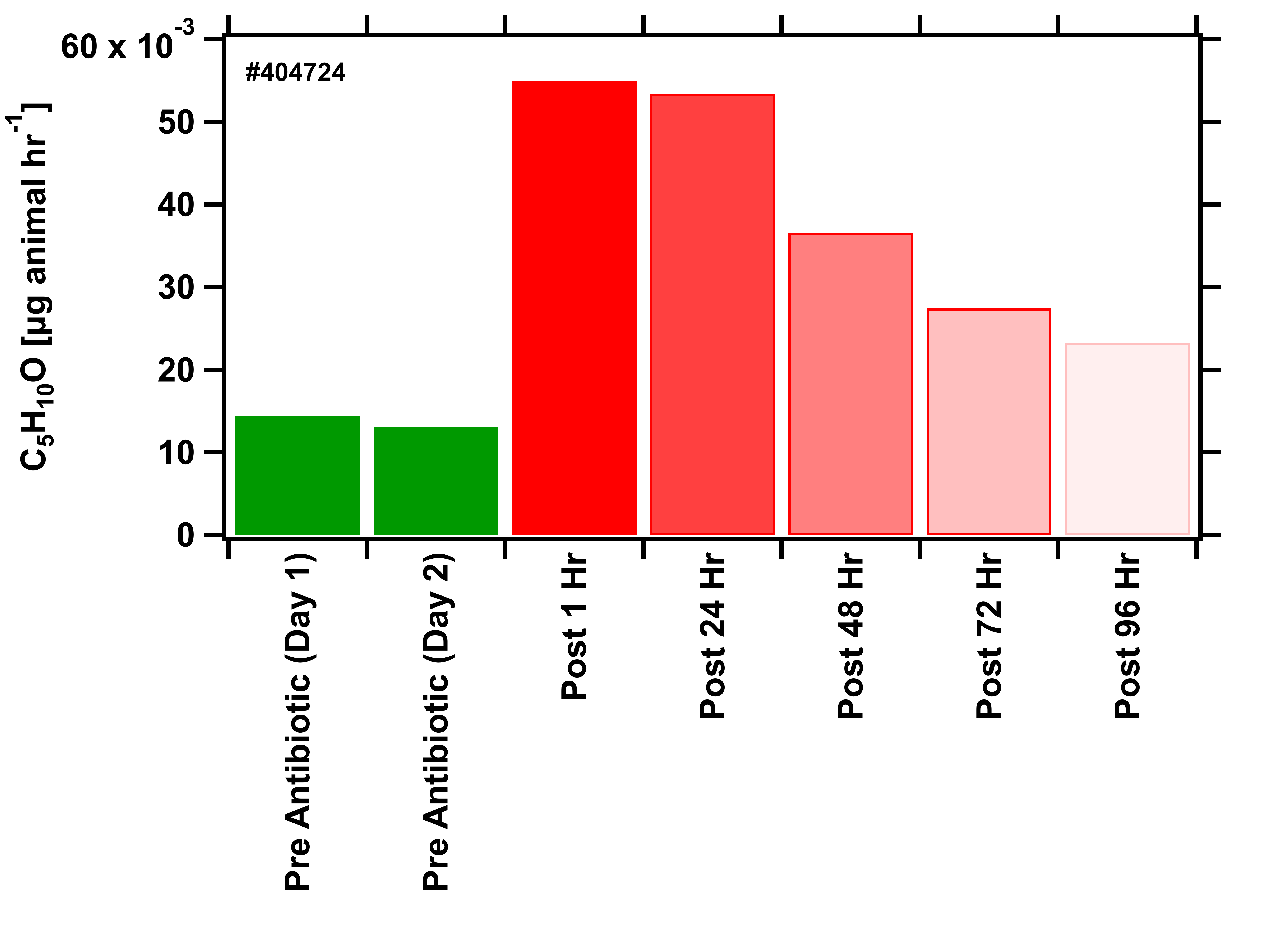
**

**Figure S23. Emission rates of C_5_H_10_O measured from healthy calf #404724 two days prior to antibiotic treatment (green), and at 1, 24, 48, 72, and 96 hours following injection with Alamycin LA 300.**

**
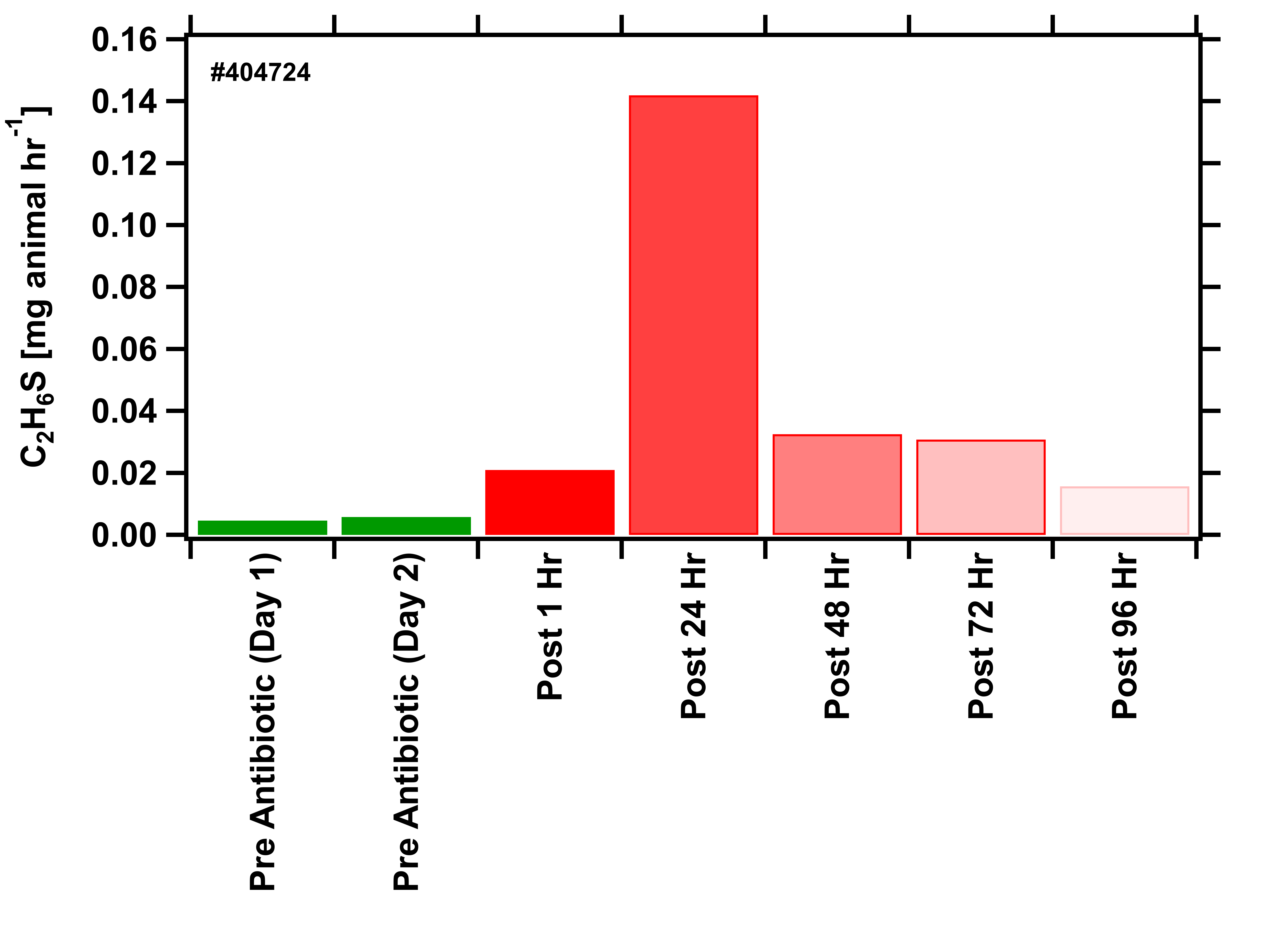
**

**Figure S24. Emission rates of dimethyl sulphide (C_2_H_6_S) measured from healthy calf #404724 two days prior to antibiotic treatment (green), and at 1, 24, 48, 72, and 96 hours following injection with Alamycin LA 300.**

**
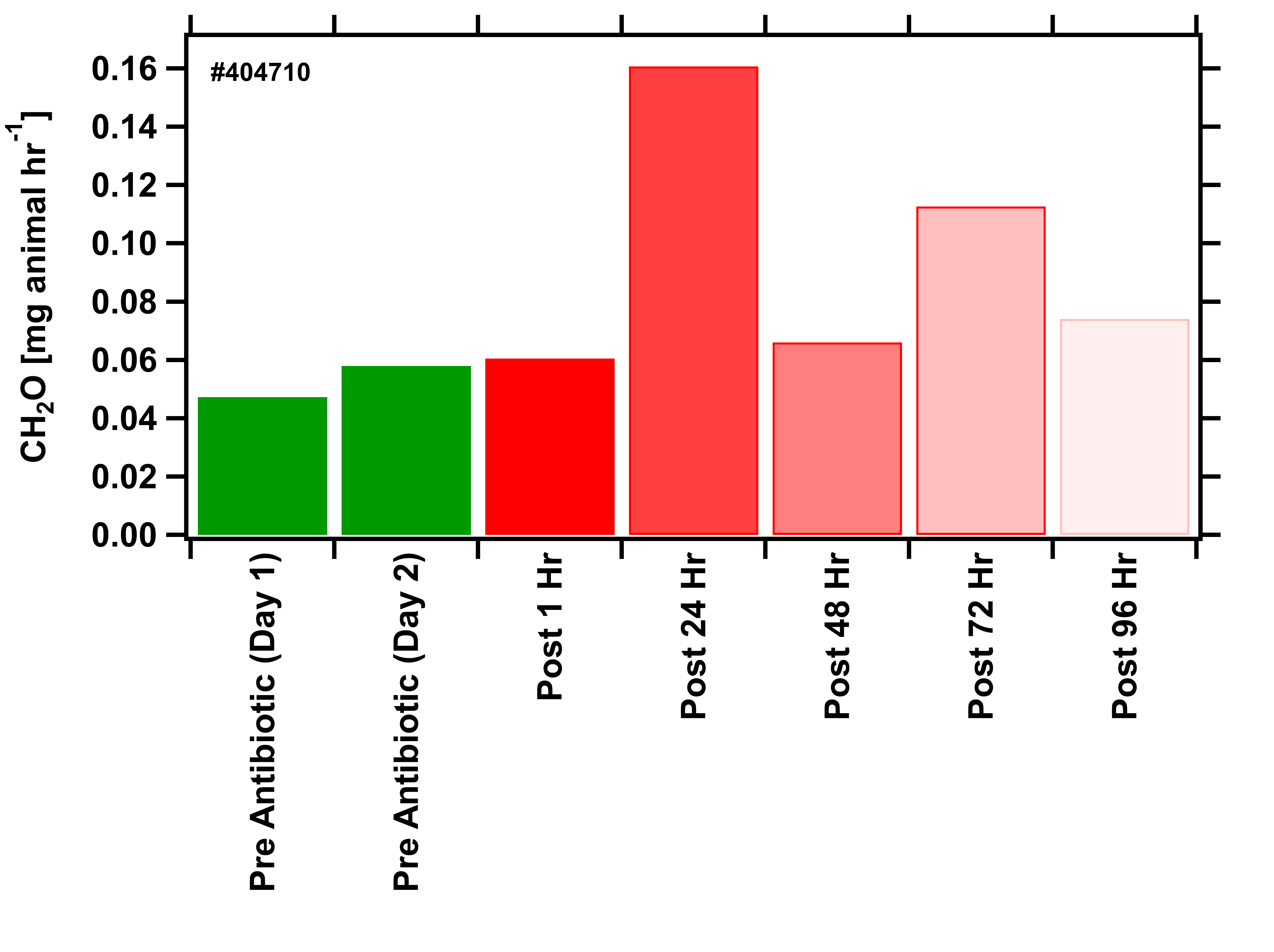
**

**Figure S25. Emission rates of formaldehyde (CH₂O) measured from healthy calf #404710 two days prior to antibiotic treatment (green), and at 1, 24, 48, 72, and 96 hours following injection with Alamycin LA 300.**

**
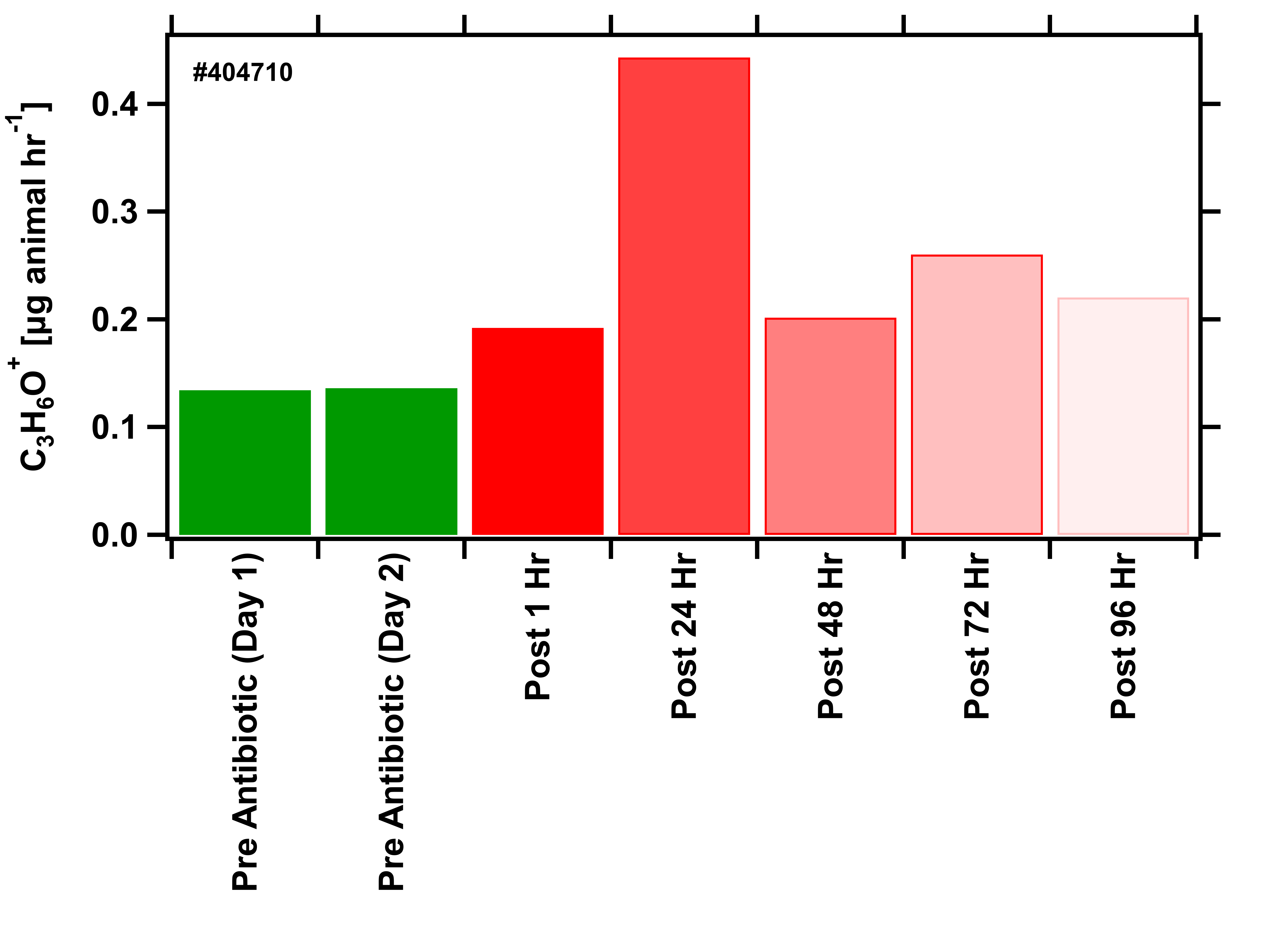
**

**Figure S26. Emission rates of acetone/propanal (C_3_H_6_O^+^) measured from healthy calf #404710 two days prior to antibiotic treatment (green), and at 1, 24, 48, 72, and 96 hours following injection with Alamycin LA 300.**

**
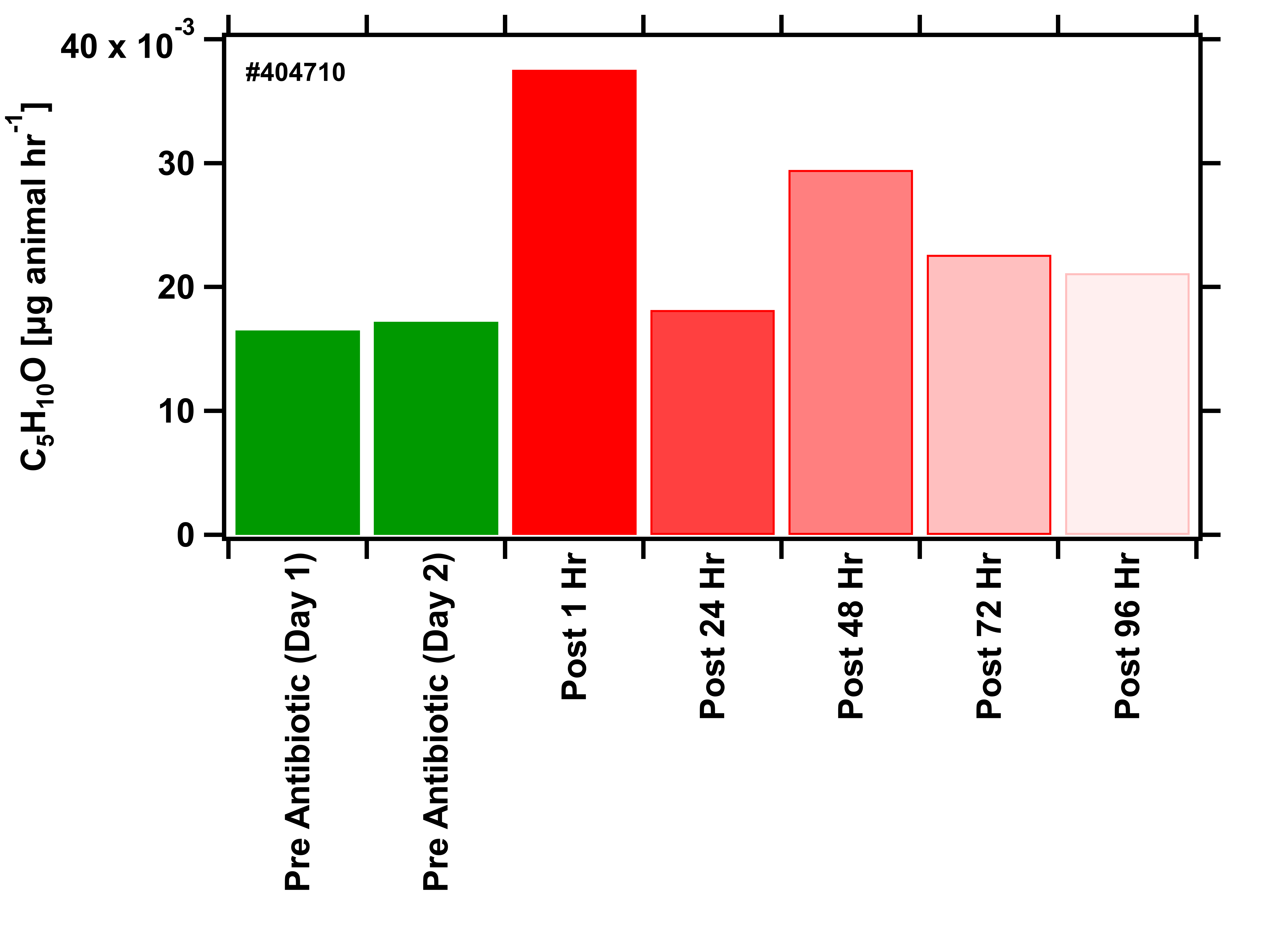
**

**Figure S27. Emission rates of C_5_H_10_O measured from healthy calf #404710 two days prior to antibiotic treatment (green), and at 1, 24, 48, 72, and 96 hours following injection with Alamycin LA 300.**

**
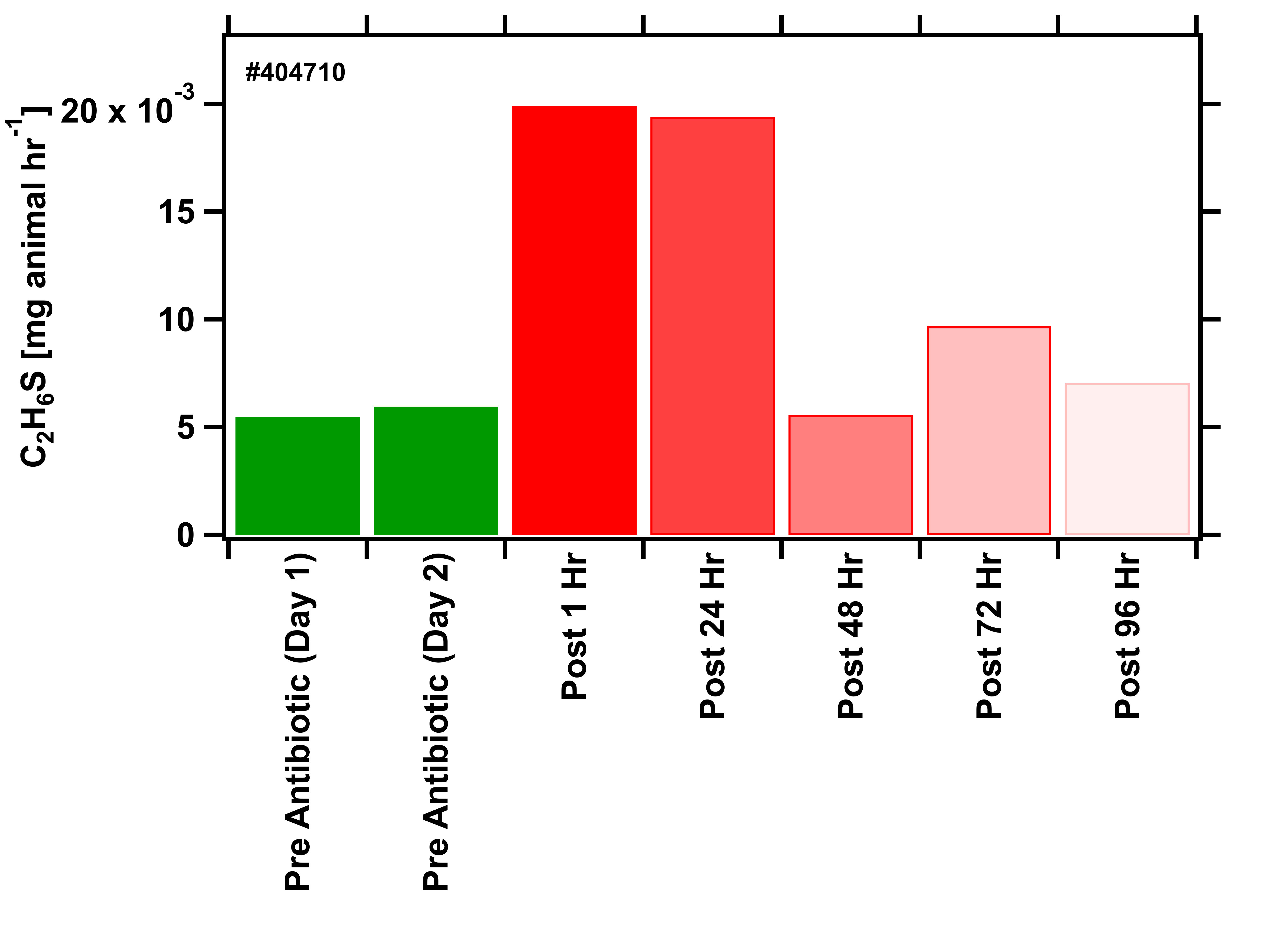
**

**Figure S28. Emission rates of dimethyl sulphide (C_2_H_6_S) measured from healthy calf #404710 two days prior to antibiotic treatment (green), and at 1, 24, 48, 72, and 96 hours following injection with Alamycin LA 300.**

**
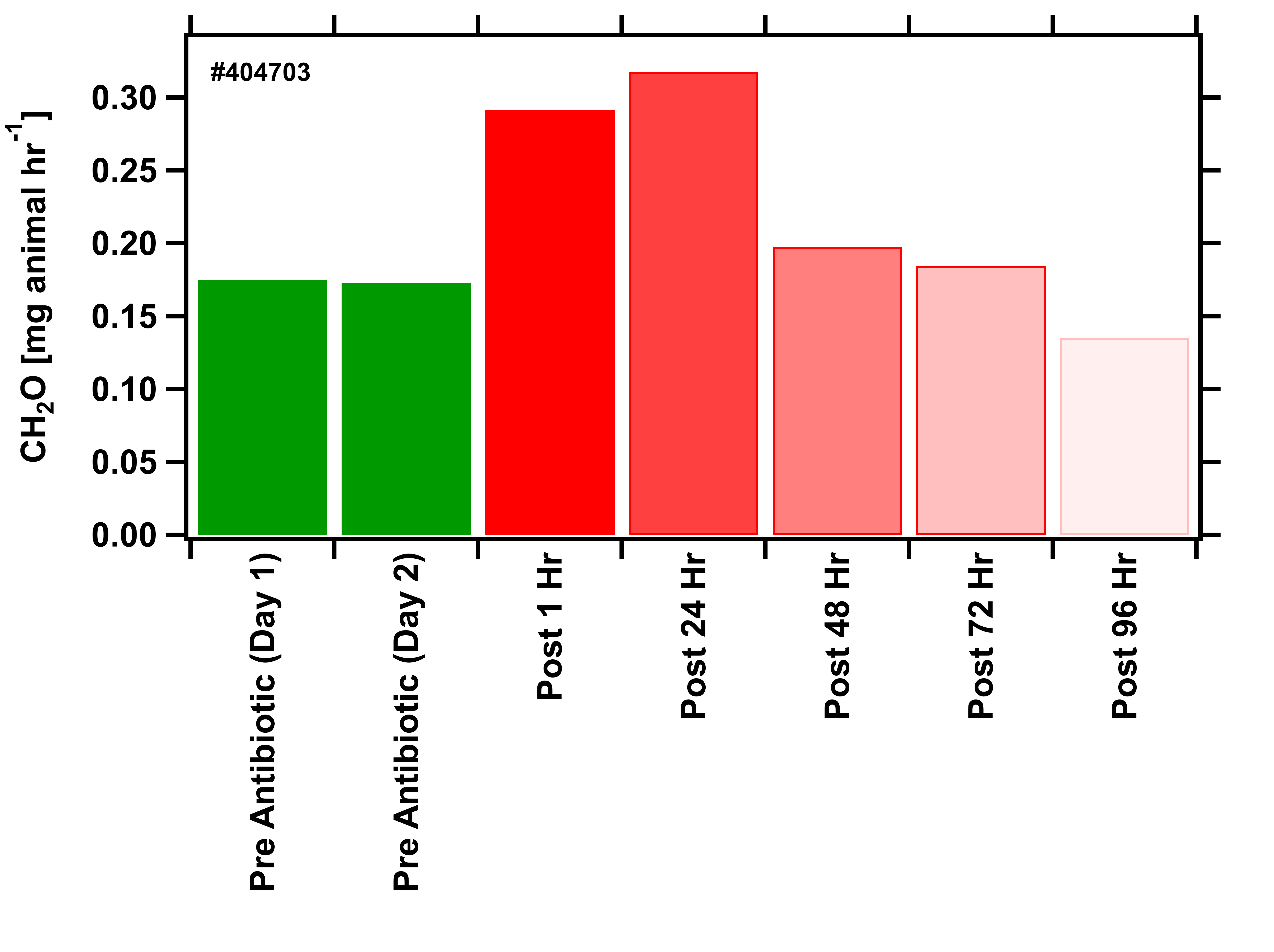
**

**Figure S29. Emission rates of formaldehyde (CH₂O) measured from healthy calf #404703 two days prior to antibiotic treatment (green), and at 1, 24, 48, 72, and 96 hours following injection with Alamycin LA 300.**

**
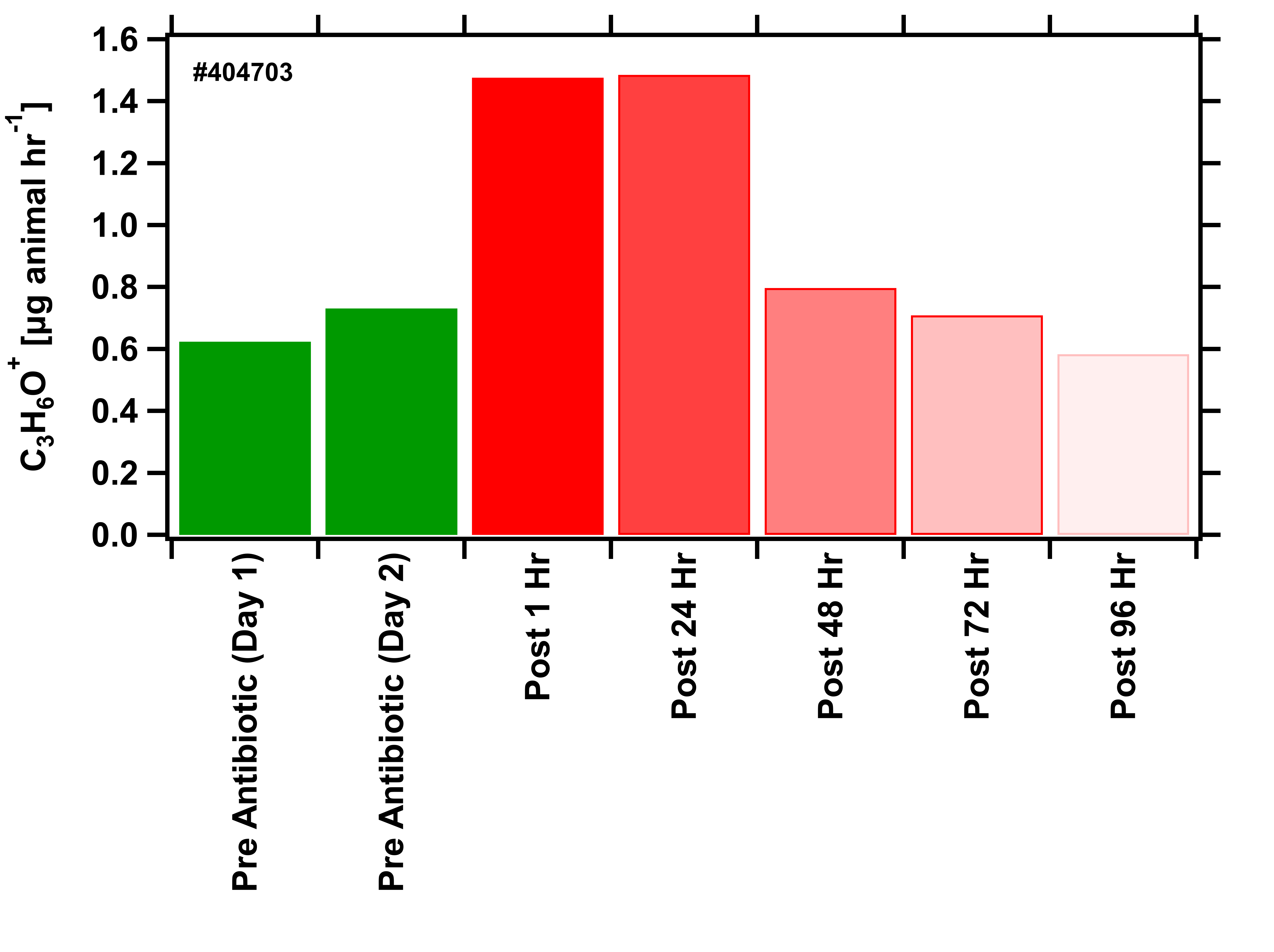
**

**Figure S30. Emission rates of acetone/propanal (C_3_H_6_O^+^) measured from healthy calf #404703 two days prior to antibiotic treatment (green), and at 1, 24, 48, 72, and 96 hours following injection with Alamycin LA 300.**

**
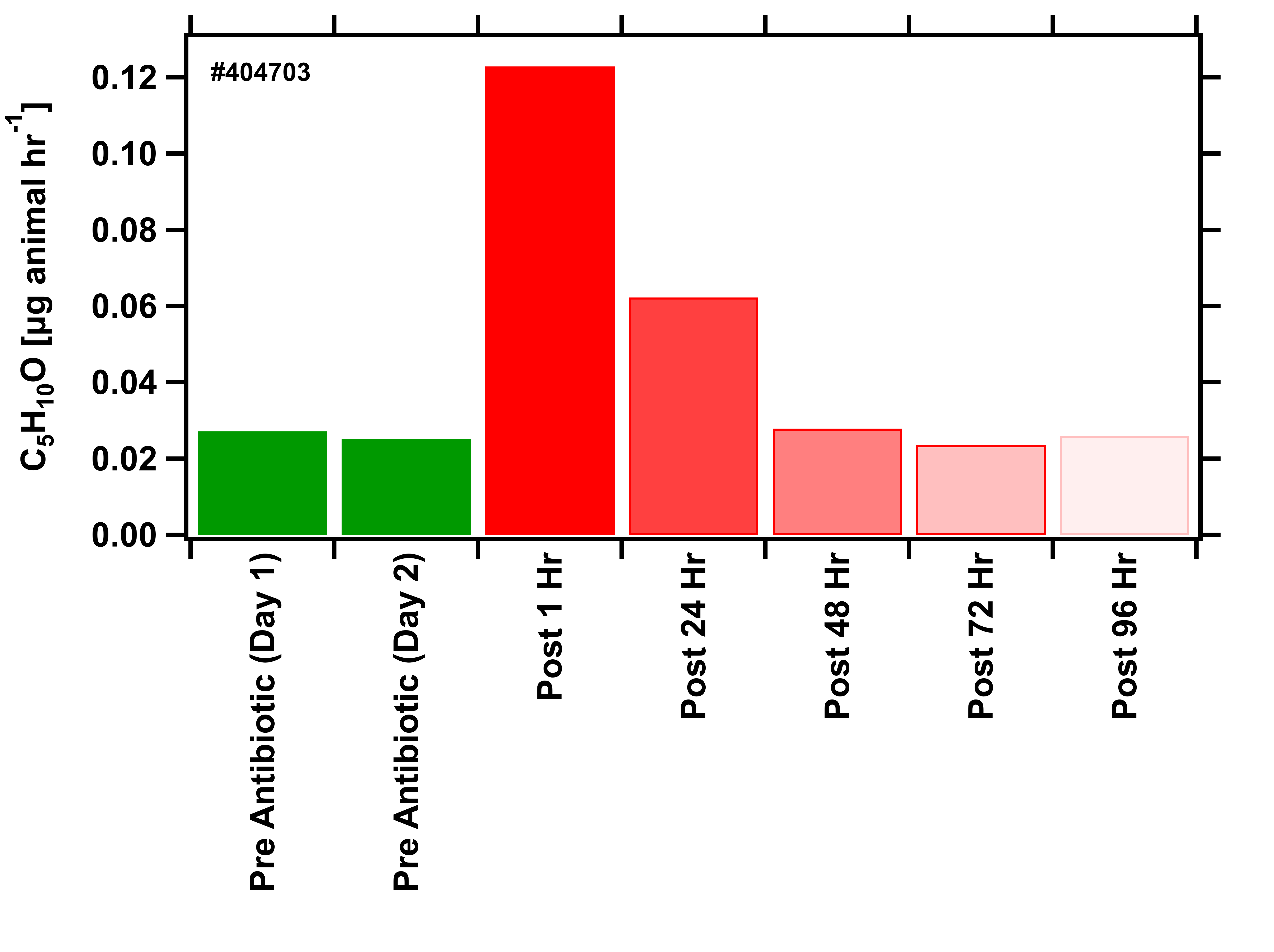
**

**Figure S31. Emission rates of C_5_H_10_O measured from healthy calf #404703 two days prior to antibiotic treatment (green), and at 1, 24, 48, 72, and 96 hours following injection with Alamycin LA 300.**

**
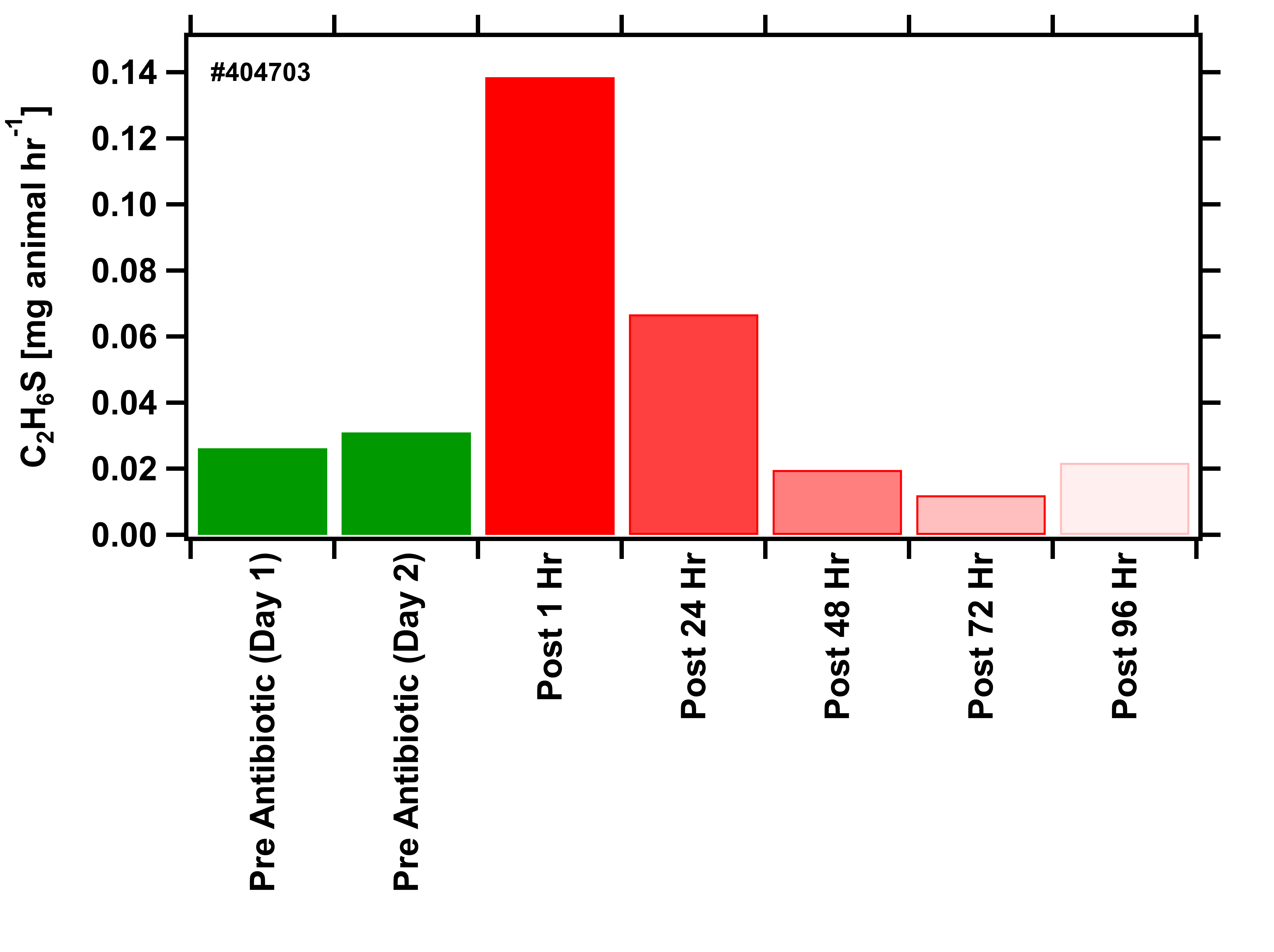
**

**Figure S32. Emission rates of dimethyl sulphide (C_2_H_6_S) measured from healthy calf #404703 two days prior to antibiotic treatment (green), and at 1, 24, 48, 72, and 96 hours following injection with Alamycin LA 300.**
