# Supplementary material for: Antibiotics confound breath-based respiratory disease detection in calves
Source: PLoS One. 2026 Jun 24;21(6):e0351838. doi: 10.1371/journal.pone.0351838 (PMC13293443; doi:10.1371/journal.pone.0351838)
Supplement: Attachment — Submitted filename: Reviewer Comments for PONE-D-25-67033.pdf [file pone.0351838.s002.pdf]

The authors measured several volatile organic compounds (VOCs) in exhaled breath using a PRT-Qi-TOF and volatile gas analyzers. Healthy and suspected ill animals were categorized using a modified Wisconsin scoring system. The VOC results showed the levels in breath depend on calf age, time of collection, and health status. Several of these VOCs appeared to be affected by antibiotic administration. Since antibiotic administration is common when animals are suspected of respiratory disease, this constitutes a confounder for BRD diagnosis using VOCs. This finding is significant as it may hinder the applicability of VOC analysis for BRD diagnosis. In general, the data is of acceptable quality with relevant statistical analyses. Results are organized into understandable figures and tables. Though many control measurements were performed, additional are needed to support the validity of sulfur-containing VOCs as BRD biomarkers. However, the text associated with these results is very difficult to understand. This significantly hinders the readability of the manuscript and needs to be heavily revised. There are grammatical and formatting errors throughout the text that need to be addressed. The manuscript is poorly cited. Relevant research exists in the literature that the authors need to include in the introduction and results sections of the manuscript. Furthermore, the authors need to be judicious when citing to ensure in-text claims are truly supported by literature. Several references that the authors cited do not include the content they claim. The manuscript presents important results that contribute to the field of exhaled breath analysis and BRD diagnosis. However, the manuscript preparation hinders the communication of the findings.

Positives:

- Measuring four replicate blanks followed by four replicate samples over two minutes is a good approach to increase confidence in the results. Similar benefit for Figure 6, where the four volatiles were measured for two days before antibiotic administration.
- The data are well summarized in the figures. The plots are well organized with color coding that helps with visualization.

Criticisms:

- Add a diagram of the experimental procedure, including the online breath sampling setup for PTR-QiTOF, MIRO MGA10-GP, and 720 VOC Analyzer. This helps readers visualize the breath collection process.
- Define the temperature units used (e.g. °C)
- Clarify the methodology throughout section 2. The procedures are difficult to understand. This is especially true for section 2.4, though not exclusively.
- Figure 1 x-axis is confusing. At first glance, it appears to be fractions of a minute until reading the caption. Please clarify this.

- Listing the full calf number designations is confusing and hard to follow. Please simply the assignments for each animal.
- In the text, explain why there are more breath samples in the pre-antibiotic group compared to the seven healthy calves treated with antibiotics in the Figure 5 PCA analysis.
- Clarify the p-values for both DMS and methanethiol. In the text, only one p-value is listed at the two listed time points (i.e., 1 h & 24 h). For two compounds, this should be four total p-values (2 compounds × 2 time points).
- Since DMS is proposed as an important exhaled VOC, the authors need to provide additional evidence that the DMS does not originate from formaldehyde sulfoxylate. At present, the authors only state that it is "plausible" that the sulfur-containing VOCs originate from the antibiotic formulation.
- Though the findings in Section 3.6 and Figure 8 are interesting, they are preliminary. The data expand the scope of the paper beyond what is appropriate. It could be included in a follow-up paper, if desired, with additional subjects.
- Throughout the paper, the wording is confusing and hard to follow. Many paragraphs are wordy and the conclusions are not clear. This needs to be rectified.

Comments on citations:

- There are more recent original research and literature reviews discussing exhaled breath analysis for disease diagnosis in both humans and animals. Such recent works should be included in the first paragraph of the introduction.
- The content in citations 12 and 13 do not match the text with which they are associated. Specifically, there is no discussion of the economic impact of BRD in the listed sources. The authors need to support this claim with other sources. Citations 12 and 13 are important pieces of literature. It would be good to include them elsewhere in the introduction.
- Citation 17 does not discuss how calf pneumonia creates "management burdens". The authors need to (i) reference a more appropriate source and/*or* (ii) to be more specific with the claim in the text.
