# Supplementary material for: Antibiotics confound breath-based respiratory disease detection in calves
Source: PLoS One. 2026 Jun 24;21(6):e0351838. doi: 10.1371/journal.pone.0351838 (PMC13293443; doi:10.1371/journal.pone.0351838)
Supplement: Attachment — Submitted filename: PONE-D-25-67033 - comments.pdf [file pone.0351838.s003.pdf]

PLOS One

Antibiotics Confound Breath-Based Respiratory Disease Detection in Calves

--Manuscript Draft--

**Manuscript Number:**	PONE-D-25-67033
**Article Type:**	Research Article
**Full Title:**	Antibiotics Confound Breath-Based Respiratory Disease Detection in Calves
**Short Title:**	Antibiotics Confound Breath-Based Respiratory Disease Detection in Calves
**Corresponding Author:**	Ben Langford<br>UK Centre for Ecology & Hydrology<br>Penicuik, Midlothian UNITED KINGDOM OF GREAT BRITAIN AND NORTHERN IRELAND
**Keywords:**	Bovine respiratory disease (BRD)<br>Breath analysis<br>Volatile organic compounds (VOCs)<br>Antibiotic treatment<br>Livestock diagnostics<br>Calf health
**Abstract:**	Breath analysis offers a promising, non-invasive approach for early disease detection in livestock, particularly for bovine respiratory disease (BRD). However, the widespread use of antibiotics in veterinary practice raises concerns about their potential to confound breath-based diagnostics. In this study, we analysed breath samples from 65 calves to evaluate the diagnostic performance of volatile organic compounds (VOCs) as biomarkers of BRD. We identified several candidate ions, including formaldehyde and acetone/propanal, that showed significant elevation at the onset of disease and a modest detection capability (AUC = 0.53-0.77). However, the strongest biomarker signals, including (C5H10O)H+ ion and dimethyl sulphide, occurred after antibiotic administration, suggesting a pharmacological influence. To isolate this effect, a secondary trial was conducted in which healthy calves were treated with oxytetracycline (Alamycin LA 300). This revealed rapid and transient changes in the breath volatilome, with significant increases in dimethyl sulphide and ($C_5H_{10}O$)H+ within 1 hour, and other VOCs within 24 hours of treatment. These findings demonstrate that antibiotics can substantially alter breath VOC profiles, potentially mimicking or masking disease signals. We conclude that breath-based diagnosis of BRD holds promise but must account for treatment history to avoid misclassification. Moreover, the reproducible and time-resolved nature of the VOC response suggests that breath analysis could also be developed as a tool for monitoring antimicrobial exposure and optimising therapeutic dosing in livestock.
**Order of Authors:**	Ben Langford<br>Johanna Brans<br>Claire Broadbent<br>Tim Gibson<br>Mark Hanlon<br>Marie Haskell<br>Laura Nicoll<br>Neil J Mullinger<br>Carol-Anne Duthie
**Additional Information:**	
**Question**	**Response**
**Financial Disclosure**	Yes

Powered by Editorial Manager® and ProduXion Manager® from Aries Systems Corporation

Enter a financial disclosure statement that describes the sources of funding for the work included in this submission. Review the submission guidelines for detailed requirements. View published research articles from *PLOS ONE* for specific examples.<br><br>This statement is required for submission and **will appear in the published article** if the submission is accepted. Please make sure it is accurate.<br><br>**Funded studies**<br>Enter a statement with the following details:<br>• Initials of the authors who received each award<br>• Grant numbers awarded to each author<br>• The full name of each funder<br>• URL of each funder website<br>• Did the sponsors or funders play any role in the study design, data collection and analysis, decision to publish, or preparation of the manuscript?<br><br>Did you receive funding for this work?	
Please add funding details.<br>as follow-up to "**Financial Disclosure**<br><br>Enter a financial disclosure statement that describes the sources of funding for the work included in this submission. Review the submission guidelines for detailed requirements. View published research articles from *PLOS ONE* for specific examples.<br><br>This statement is required for submission and **will appear in the published article** if the submission is accepted. Please make sure it is accurate.	This work was funded as part of the Innovate UK grant (Project # 10072590): DETECT.

Powered by Editorial Manager® and ProduXion Manager® from Aries Systems Corporation

**Funded studies**<br>Enter a statement with the following details:<br>• Initials of the authors who received each award<br>• Grant numbers awarded to each author<br>• The full name of each funder<br>• URL of each funder website<br>• Did the sponsors or funders play any role in the study design, data collection and analysis, decision to publish, or preparation of the manuscript?<br><br>Did you receive funding for this work?"	
Please select the country of your main research funder (please select carefully as in some cases this is used in fee calculation).<br>as follow-up to "**Financial Disclosure**<br><br>Enter a financial disclosure statement that describes the sources of funding for the work included in this submission. Review the submission guidelines for detailed requirements. View published research articles from *PLOS ONE* for specific examples.<br><br>This statement is required for submission and **will appear in the published article** if the submission is accepted. Please make sure it is accurate.<br><br>**Funded studies**<br>Enter a statement with the following details:<br>• Initials of the authors who received each award<br>• Grant numbers awarded to each author<br>• The full name of each funder<br>• URL of each funder website<br>• Did the sponsors or funders play any role in the study design, data collection and analysis, decision to publish, or preparation of the manuscript?<br><br>Did you receive funding for this work?"	UNITED KINGDOM - GB
**Competing Interests**	The authors have declared that no competing interests exist.

Use the instructions below to enter a competing interest statement for this submission. On behalf of all authors, disclose any competing interests that could be perceived to bias this work—acknowledging all financial support and any other relevant financial or non-financial competing interests.<br><br>This statement is required for submission and **will appear in the published article** if the submission is accepted. Please make sure it is accurate and that any funding sources listed in your Funding Information later in the submission form are also declared in your Financial Disclosure statement.<br><br>View published research articles from *PLOS ONE* for specific examples.<br><br>**NO authors have competing interests**<br><br>Enter: *The authors have declared that no competing interests exist.*<br><br>**Authors with competing interests**<br><br>Enter competing interest details beginning with this statement:<br><br>*I have read the journal's policy and the authors of this manuscript have the following competing interests: [insert competing interests here]*<br><br>* typeset	
**Ethics Statement**<br><br>Enter an ethics statement for this submission. This statement is required if the study involved:<br><br>• Human participants<br>• Human specimens or tissue<br>• Vertebrate animals or cephalopods<br>• Vertebrate embryos or tissues	The study was conducted under ethical approval from SRUC's Animal Experiments Committee (Approval number: AEX 2024-024 DAI).

• Field research<br><br>Write "N/A" if the submission does not require an ethics statement.<br><br>General guidance is provided below. Consult the submission guidelines for detailed instructions. **Make sure that all information entered here is included in the Methods section of the manuscript.**	

Powered by Editorial Manager® and ProduXion Manager® from Aries Systems Corporation

**Format for specific study types**<br><br>**Human Subject Research (involving human participants and/or tissue)**<br>• Give the name of the institutional review board or ethics committee that approved the study<br>• Include the approval number and/or a statement indicating approval of this research<br>• Indicate the form of consent obtained (written/oral) or the reason that consent was not obtained (e.g. the data were analyzed anonymously)<br><br>**Animal Research (involving vertebrate animals, embryos or tissues)**<br>• Provide the name of the Institutional Animal Care and Use Committee (IACUC) or other relevant ethics board that reviewed the study protocol, and indicate whether they approved this research or granted a formal waiver of ethical approval<br>• Include an approval number if one was obtained<br>• If the study involved *non-human primates*, add *additional details* about animal welfare and steps taken to ameliorate suffering<br>• If anesthesia, euthanasia, or any kind of animal sacrifice is part of the study, include briefly which substances and/or methods were applied<br><br>**Field Research**<br><br>Include the following details if this study involves the collection of plant, animal, or other materials from a natural setting:<br>• Field permit number<br>• Name of the institution or relevant body that granted permission	
**Data Availability**<br><br>Authors are required to make all data underlying the findings described fully available, without restriction, and from the time of publication. PLOS allows rare exceptions to address legal and ethical concerns. See the PLOS Data Policy and FAQ for detailed information.	Yes - all data are fully available without restriction

A Data Availability Statement describing where the data can be found is required at submission. Your answers to this question constitute the Data Availability Statement and **will be published in the article**, if accepted.<br><br>**Important:** Stating 'data available on request from the author' is not sufficient. If your data are only available upon request, select 'No' for the first question and explain your exceptional situation in the text box.<br><br>Do the authors confirm that all data underlying the findings described in their manuscript are fully available without restriction?	
**Describe where the data may be found in full sentences. If you are copying our sample text, replace any instances of XXX with the appropriate details.**<br><br>• If the data are **held or will be held in a public repository**, include URLs, accession numbers or DOIs. If this information will only be available after acceptance, indicate this by ticking the box below. For example: *All XXX files are available from the XXX database (accession number(s) XXX, XXX.*).<br>• If the data are all contained **within the manuscript and/or Supporting Information files**, enter the following: *All relevant data are within the manuscript and its Supporting Information files.*<br>• If neither of these applies but you are able to provide **details of access elsewhere**, with or without limitations, please do so. For example:<br><br>*Data cannot be shared publicly because of [XXX]. Data are available from the XXX Institutional Data Access / Ethics Committee (contact via XXX) for researchers who meet the criteria for access to confidential data.*<br><br>*The data underlying the results presented in the study are available from (include the name of the third party*	All breath measurements are available from the Zenodo database (https://zenodo.org/records/17940806 DOI: 10.5281/zenodo.17940805).

and contact information or URL).<br>• This text is appropriate if the data are owned by a third party and authors do not have permission to share the data.<br><br>* typeset	
Additional data availability information:	Tick here if the URLs/accession numbers/DOIs will be available only after acceptance of the manuscript for publication so that we can ensure their inclusion before publication.

Powered by Editorial Manager® and ProduXion Manager® from Aries Systems Corporation

Manuscript

Click here to access/download;Manuscript;Langford_et_al_PlosOne_2026.d

Antibiotics Confound Breath-Based Respiratory Disease Detection in Calves

Ben Langford[1], Johanna Brans[2], Claire Broadbent[2], Tim Gibson[3], Mark Hanlon[1], Marie Haskell[2], Laura Nicoll[2], Neil J. Mullinger[1] and Carol-Anne Duthie[2]

1. UK Centre for Ecology and Hydrology, Bush Estate, Penicuik, EH26 0QB, United Kingdom
2. SRUC, West Mains Road, Edinburgh EH9 3JG, United Kingdom
3. RoboScientific Ltd, Espace North, 181 Wisbech Road, Littleport, Cambridgeshire, CB6 1RA, United Kingdom

Abstract

Breath analysis offers a promising, non-invasive approach for early disease detection in livestock, particularly for bovine respiratory disease (BRD). However, the widespread use of antibiotics in veterinary practice raises concerns about their potential to confound breath-based diagnostics. In this study, we analysed breath samples from 65 calves to evaluate the diagnostic performance of volatile organic compounds (VOCs) as biomarkers of BRD. We identified several candidate ions, including formaldehyde and acetone/propanal, that showed significant elevation at the onset of disease and a modest detection capability (AUC = 0.53-0.77). However, the strongest biomarker signals, including $(C_5H_{10}O)H^+$ ion and dimethyl sulphide, occurred after antibiotic administration, suggesting a pharmacological influence. To isolate this effect, a secondary trial was conducted in which healthy calves were treated with oxytetracycline (Alamycin LA 300). This revealed rapid and transient changes in the breath volatilome, with significant increases in dimethyl sulphide and $(C_5H_{10}O)H^+$ within 1 hour, and other VOCs within 24 hours of treatment. These findings demonstrate that antibiotics can substantially alter breath VOC profiles, potentially mimicking or masking disease signals. We conclude that breath-based diagnosis of BRD holds promise but must account for treatment history to avoid misclassification. Moreover, the reproducible and time-resolved nature of the VOC response suggests that breath analysis could also be developed as a tool for monitoring antimicrobial exposure and optimising therapeutic dosing in livestock.

Introduction

Breath analysis has emerged as a powerful tool for non-invasive disease detection in human healthcare, offering real-time diagnostic insights through the analysis of volatile organic compounds (VOCs) [1-3]. The technique has been applied to a range of clinical challenges for humans, including the detection of metabolic disorders[4], cancer biomarkers[5], and infectious diseases[3], owing to its ability to reflect systemic physiological changes without the need for invasive sampling [1-3]. More recently, breathomics has begun gaining traction in animal health research, with growing interest in its application to veterinary medicine[6, 7]. Early studies have demonstrated its potential for identifying paratuberculosis [8] , monitoring ketosis in dairy cows [9, 10] , and even detecting pregnancy through VOC profiling[11].

Given its promise, breath-based diagnostics could play a transformative role in the detection of bovine respiratory disease (BRD), one of the most economically significant illnesses affecting cattle worldwide[12, 13]. BRD is a complex syndrome, initiated by viral infections that predispose young calves to secondary bacterial invasion, leading to pneumonia and impaired respiratory function[14]. With calf pneumonia affecting 20–30% of herds in the UK[14], the economic and welfare implications are substantial[15] and include reduced

growth rates, increased veterinary costs[16], and considerable management burdens[17, 18]. Traditional diagnostic approaches rely on visual observation of symptoms, yet these signs are often non-specific, leading to delayed intervention. Breath analysis offers an alternative: a metabolic fingerprint capable of detecting physiological changes linked to disease onset, with some studies showing early promise [7, 19-23]. Moreover, a new generation of low-cost VOC sensors is emerging, offering the potential for on-farm diagnostic tools, that can be incorporated into existing infrastructure such as close to feeders and water troughs that animals visit several times a day. However, it must be ensured that reliable and disease-specific breath signatures can be identified.

A key challenge in breath-based disease detection, however, is the widespread use of antibiotics in livestock management. Antibiotics remain a cornerstone of BRD treatment, often administered at first suspicion of illness and, in some cases, prophylactically in high-risk groups such as recently weaned calves[24]. While effective in controlling bacterial infections, their potential influence on breath biomarkers remains an open question. If VOC shifts caused by antibiotics obscure or mimic disease-related signals, it may complicate diagnostic accuracy.

Recent studies in human breathomics have demonstrated the presence of antibiotic metabolites in exhaled breath, suggesting that pharmaceutical intervention can directly alter VOC profiles[25]. This raises concerns about whether breath-based diagnostics truly capture pathology or if treatment effects inadvertently shape biomarker interpretation. Understanding how antibiotics, or indeed vaccination, modify breath composition is essential for ensuring reliable disease detection.

In this study, we investigate breath-based detection of BRD within a farm setting where disease management involved antibiotic treatment. We analysed breath samples from 65 calves to characterise VOC patterns in diseased and healthy animals, assessing the potential for breath-based disease detection, including piloting the use of low-cost sensors (phase 1). To further probe the influence of antibiotics, we conducted a secondary trial (phase 2) administering treatment to healthy calves, tracking breath composition over multiple time points.

2. Methods

2.1 Online Breath Sampling

Breath samples were collected using a custom-built polycarbonate mask which was 15 cm in diameter and 15 cm long. The open end of the cylinder was covered with a silicone lid (Dunelm) with an aperture of 8 cm, to form an effective seal around the animal's muzzle. At the end of the cylinder, a charcoal respirator filter (3M Gas & Vapour filter, GF22 A2, Arco Limited, Hull, UK) was fitted to help limit the concentration of VOCs in the air entering the mask. Exhaled breath was sampled through a ¼" OD tube (I.D. 1/8") which connected to the base of the mask and passed through a Teflon filter to remove any particulates from the sampled air. Flexible PFA tubing was used for the first metre of sample and then the air going for online analysis was carried along a 6 m, ¼" OD Sulfinert® coated stainless steel tube (I.D. 1/8"). The tubing was inserted into a custom-built heating sleeve (Signal Group Ltd, Camberley, UK) set to 140 °C. This maintained an internal air temperature of 45 °C, which prevented condensation and limited VOC adsorption onto the tube walls. The flow rate was maintained at 7 LPM under a pressure of 800 mbar, both of which were measured, together with air temperature via a thermal mass flowmeter (TSI Model 4040, USA), mounted downstream of the gas analysers.

Air from the sample line was sub-sampled by a proton transfer reaction time of flight mass spectrometer with quadrupole ion guide (PTR-QiTOF, IONICON Analytik GmbH, Austria)[26]. The instrument was operated with a drift tube pressure, voltage and temperature of 3.7 mbar, 830 V and 80 °C, respectively. These settings ensured the ratio between electric field (*E*) and number density of gas molecules within the drift tube (*N*) was maintained at a value of 120 Td. H_3O^+ reagent ions were used to detect VOCs with a proton affinity greater than that of water.

The PTR-Qi-TOF was calibrated at both the beginning and end of each measurement period using a multicomponent gas standard (Apel-Riemer Environmental Ltd). Following standard practice[27, 28], an instrument-specific transmission curve was derived from the known concentrations of the calibrated compounds. This curve was then combined with published reaction rate constants to convert ion signal intensities to concentrations expressed in milligrams per metre cubed (mg m^{-3}) for all detected ions. The transmission-based quantification method carries an associated uncertainty of approximately 25%[27].

Measurements of carbon dioxide (CO_2), were made using a MIRO MGA10-GP gas analyser (MIRO Analytical, Wallisellen, Switzerland). This instrument, which uses direct laser absorption spectroscopy, subsampled downstream of the PTR-QiTOF. The MIRO sampled at a rate of 1 Hz, with data logged directly to the PTR-QiTOF. CO_2 is also detectable by PTR-QiTOF instruments at high concentrations due to endothermic proton transfer reactions occurring at the transition point between drift tube and time-of-flight chamber. This is enhanced in instruments with a quadrupole ion guide due to the higher energies present in this region [20]. The CO_2 signal is typically suppressed by tuning the source valve, but here a higher CO_2 signal was desirable to allow synchronisation between the two instruments by searching for the maximum in a cross-covariance of the two CO_2 signals, with the difference typically 3 seconds. The higher resolution (2 Hz) measurements of CO_2 made by the PTR-QiTOF were used for the analysis following calibration against the MIRO data. Allowing a higher CO_2 signal came at the cost of compromising the acetaldehyde signal at $(C_2H_4O)H^+$, which sits on the shoulder of the much larger CO_2 peak.

2.2 Emission Rates of Breath Volatiles

The emission rate of volatiles exhaled on the breath of calves ($E_{exhaled}$) was calculated in units of mass per animal per hour (e.g. mg animal hr^{-1}), depending on compound abundance, as [29]:

$$E_{exhaled} = (x_{breath} - x_{bg}) \cdot TV \cdot RR$$

Where χ_{breath} is the concentration of the target VOC (e.g. mg m^{-3}) measured from the mask and χ_{bg} is the concentration of target VOC measured from a closed mask immediately prior to fitting to the animal, which forces air through the charcoal filter and acts as a blank. Mask and mask background were both measured for a period of 3 minutes for each animal sample, with the median value used in the calculation. The tidal volume (TV) was calculated for each animal and based on the value of 8×10^{-6} m^3 per kg of body weight from Dißmann et al. [30]. Cattle were weighed at the start and end of the study, and interim values estimated based on a linear interpolation between the two weights. Respiration rates were calculated for each animal by counting the average breaths per minute for each sample which were clearly visible in the CO_2 data (see Fig 1B).

2.3 Low-cost sensors – VOC sensing array

As part of a proof-of-concept trial, a Model 720 VOC Analyser (RoboScientific Ltd, UK) was used to assess the feasibility of breath sampling in calves. The analyser was connected to

the outflow tubing of the breath sampling mask and was trialled on several animals during the study period, rather than being applied to the full experimental cohort. Sampling was conducted in two stages: first, four replicate background air samples were collected over a two-minute period with the mask in blanked configuration. This was followed by breath sampling immediately after fitting the mask to the calf, again comprising four replicates over a two-minute period.

The 720 VOC Analyser comprises an array of 24 individual sensors, each embedded with distinct organic semiconducting materials. During each sampling event, the analyser captures sensor responses characterised by parameters such as peak height, integrated area under the response curve, absorption and desorption rate, as selected by the operator. These multivariate data were analysed both individually, for example, to compare specific sensor responses with known VOCs identified via PTR-QiTOF, and collectively, to assess changes in breath profiles over time. Cosine similarity, expressed in degrees, was used to quantify the divergence between each diseased day and the healthy baseline measurement (see section below for definitions), providing a measure of how VOC signatures evolved throughout the course of disease. Cosine similarity is defined as:

$$\theta = cos^{-1}\left(\frac{\boldsymbol{A}\cdot\boldsymbol{B}}{||\boldsymbol{A}||\,||\boldsymbol{B}||}\right) \times \left(\frac{180}{\pi}\right), \quad (2)$$

where **A** and **B** denote the feature vectors extracted from the VOC profiles of two individual breath samples. The resulting angle θ represents the degree of similarity between the vectors: 0° indicates a perfect match, while 180° indicates complete opposition.

2.5 Animals, Health Assessment and Treatments

A total of 65 calves participated in the trial, with an approximately equal distribution of sexes. Each animal was born to a Holstein dam—a dairy breed—but sired by bulls representing three beef breeds: Hereford, Aberdeen Angus, and British Blue[31].

Feeding consisted of 4 litres of milk replacer (Carr's Billington Vitality calf milk) administered twice daily at 08:00 and 15:00. Barley straw and starter pellets (Davidson's rapid starter pellets, 2359) were available ad libitum. Breath sampling generally occurred four to five hours after the morning feed, although timing varied depending on animal availability and daily sampling requirements.

The health of each animal was assessed daily by trained technicians following a modified Wisconsin scoring system [32]. The scoring system assesses each animal based on scores (0-3) for the following five categories: rectal temperature, ear droop, nasal discharge, eye discharge and cough. We did not include an assessment of faecal consistency as the animals were housed in a group and any faeces could not be reliably attributed to individual animals. A cumulative score of 5 or above classifies the animal as diseased. For this study, we additionally required that the rectal temperature was above 39.5 degrees. Under the terms of the Home Office licence for the trial, a total of 5 healthy samples could be taken from each animal, with an additional 5 consecutive samples permitted for any animal whose overall score indicated that it was diseased, with up to two diseased events permitted for sampling within the trial period.

Scoring was done in the morning, with those animals scheduled for breath analysis, either healthy or identified as diseased, sampled in the afternoon, typically between 1 and 2 pm and always before the afternoon feed. This meant samples were taken at least 4 hours after the last milk feed, limiting the influence of exogenous VOC emissions.

While the modified Wisconsin scoring system has proven an effective tool at identifying BRD, it has limitations. For example, eye infections, allergies and environmental irritants including ammonia and dust can generate symptoms similar to BRD and yield false positives. Here, the inclusion of a temperature threshold helped to limit such instances, but misdiagnosis due to other systemic infections (e.g. gastrointestinal), that might cause fever, ear droop and other symptoms indicative of BRD, cannot be ruled out.

We also recognise that BRD is a catch all term for respiratory illness brought about through bacterial or viral pathogens or a combination of both. In this study we did not acquire any nasal swabs for polymerase chain reaction testing (PCR) and, therefore, it is not possible to determine which active pathogens might be present in any individual.

Animals were not immunised before the trial. Instead, when scored as diseased, an animal would be sampled and then given relevant treatment. This typically involved intramuscular injections of Metacam and Almycin. In two cases, additional doses of Zactran and Synulox were given where the disease didn't respond to the original treatment.

The study was conducted under ethical approval from SRUC's Animal Experiments Committee (Approval number: AEX 2024-024 DAI).

2.5 Assessment of antibiotic breath metabolites

To evaluate the impact of therapeutic antibiotic treatment on breath VOC profiles, a second trial was conducted. A cohort of 10 healthy Aberdeen Angus Cross calves (seven males, three females, aged 28–43 days), all from a dairy dam, was selected. Daily breath samples were collected over two consecutive days to establish a baseline. On the third day, animals received Alamycin LA 300 (Norbrook), a long-acting, broad-spectrum injectable antibiotic containing 300 mg/mL of oxytetracycline dihydrate as the active ingredient. The formulation also includes excipients such as magnesium oxide and sodium formaldehyde sulfoxylate, which contribute to its extended-release profile. A dosage of 1 mL per kg of body weight was administered intramuscularly. Following treatment, breath samples were collected at approximately 1, 24-, 48-, 72-, and 96-hours post-administration to monitor VOC changes over time. This product was also used during the initial on-farm trials, providing a consistent pharmacological reference across study phases. The same health scoring system was adopted (daily scoring) throughout to confirm that calves were healthy throughout this trial period.

3. Results and Discussion

3.1 Breath Sampling

During the main study, measurements were made from 65 cattle, from two batches, between May-July and Sep-Oct 2024. A total of 398 breath samples were taken using the mask setup described in Section 2.1. Two calves were euthanised following illnesses unrelated to BRD and a third was removed from the trial due to bloat.

Figure 1a shows a typical breath sample of CO_2 and acetone/propanal ($(C_3H_6O)H^+$). The first three minutes represent the background period where the mask was sealed with a silicone lid to force air through the charcoal filter and provide a sample blank. The next three minutes show the period where the mask was placed on the calf to obtain the breath sample. The arrows indicate the portion of the data that were averaged (median) to provide values of x_{breath} and x_{bg}. The CO_2 signal shown was measured by the PTR-QiTOF and calibrated against the CO_2 signal obtained from the MIRO.

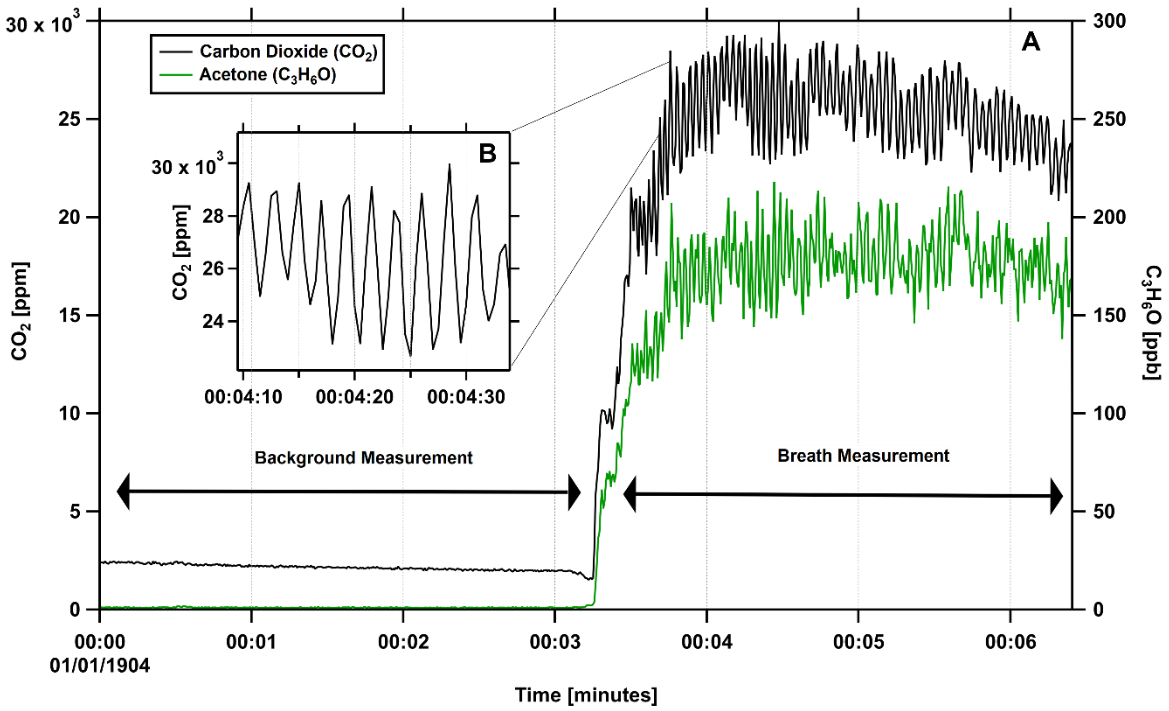

Figure 1. Example breath measurement, showing the background phase (0–3 minutes) and the breath phase (3–6 minutes). Panel B provides a close-up of the CO_2 signal used to calculate the respiration rate for each animal.

In total, 21 disease events were captured from 20 individuals, with 103 breath samples captured during the 5-day diseased period. The remaining healthy breath samples were carefully screened to remove any samples where the animal went on to become diseased in the subsequent 4 days. This left 204 ‘true’ healthy samples, taken from 41 individuals.

The PTR-TOF detected 86 ions that were consistently present in the breath of cattle. Molecular formula could be assigned to 55 of these ions with the remaining 31 reported as their protonated mass-to-charge ratio. The full list of compounds and molecular formula are listed in Table S2 of the Supplementary Information.

3.2 Disentangling Growth Effects from Potential Disease Signals in Calf Breath VOCs

During the early weeks and months of a calf’s life, rapid growth significantly influences the emission of volatile organic compounds (VOCs) in the breath, largely due to developmental changes such as increased lung capacity. These growth-related changes can be substantial [31] and must be accounted for to accurately distinguish disease-induced alterations in the volatilome from those caused by natural growth.

Figure 2A presents the growth curve of CO_2 emission rates in the breath of healthy calves. Green data points represent measurements from “true” healthy animals, those with no disease within ±4 days of sampling. The fitted curve, along with the 95% confidence interval ($1.96 \times \sigma$), shows that CO_2 emission rates nearly double over the monitored age range, emphasising the necessity of age correction when evaluating breath-based disease biomarkers. When breath data obtained from calves during episodes of disease (5 consecutive days post-onset, shown in red) are added, only one sample falls outside the healthy confidence interval. This suggests that CO_2 emissions are not significantly affected by respiratory disease in this cohort.

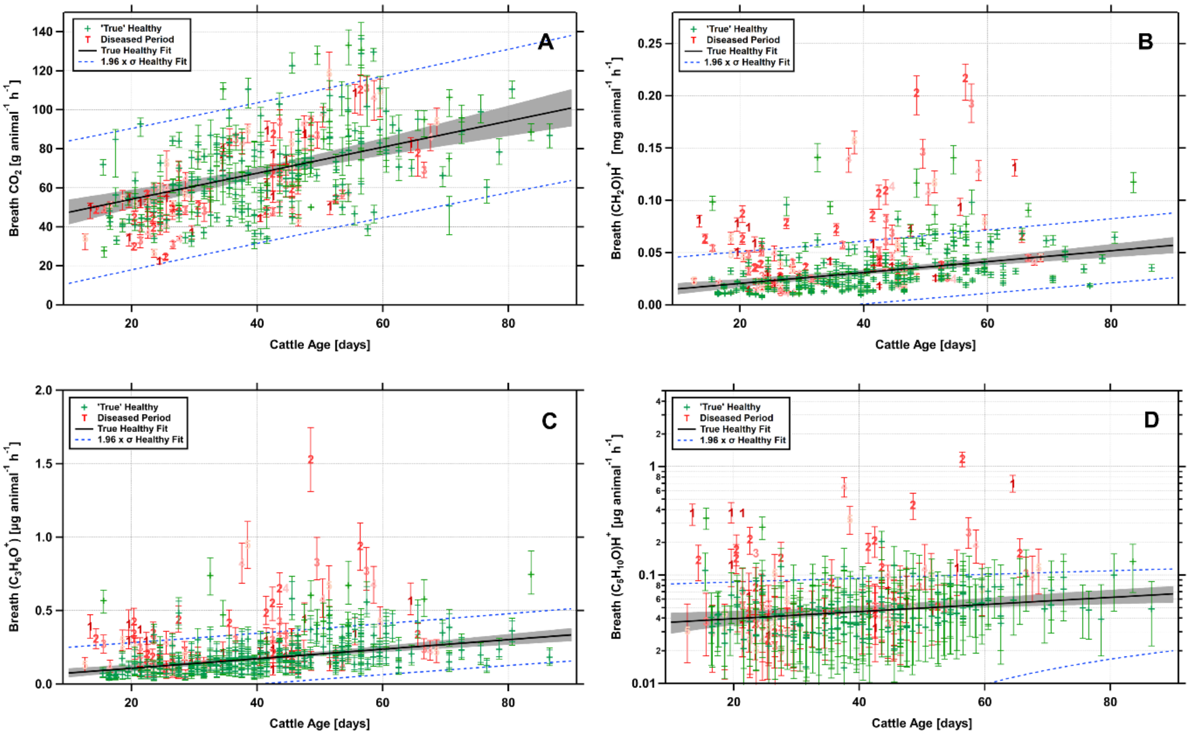

Figure 2. Breath emission rates of CO_2 (panel A), formaldehyde $(CH_2O)H^+$ (panel B), acetone/propanal $(C_3H_6O)^+$ (panel C), and $(C_5H_{10}O)H^+$ (panel D) plotted against calf age. Green data points represent measurements from "true" healthy calves (i.e., no recorded disease within four days before or after sampling). Red data points represent breath samples collected during a five-day window following a respiratory disease event. Red symbols are labelled to indicate the specific day post-onset (days 1–5) on which the sample was taken. Error bars indicate the interquartile range.

Figure 2B illustrates the same analysis for the $(CH_2O)H^+$ ion, attributed to formaldehyde, one of the most abundant compounds emitted from both healthy and diseased animals. Among the diseased samples, 32 exceed the confidence interval, corresponding to 15 individuals, from a possible 21. Additionally, 19 healthy samples from nine individuals also fall above the threshold.

In Figure 2C, the $(C_3H_6O^+)$ ion is shown, which likely reflects acetone/propanal detected directly at its monoisotopic mass (m/z 58.0419) via charge transfer from residual O_2^+ ions present as impurity species in the H_3O^+ drift tube. A total of 31 diseased samples (16 individuals) exceeded the threshold, compared to 18 healthy samples (10 individuals).

The m/z 87.08 ion in Figure 2D relates to a possible mix of 3-methylbutanal, 2-pentanone, and pentanal. This ion showed 27 threshold exceedances over the five-day diseased period relating to 15 diseased animals and 13 exceedances relating to 13 healthy animals.

Table 1 summarises the performance of the top 15 ions detected by PTR-QiTOF, ranked by the number of diseased individuals exhibiting threshold crossings. For example, $C_2H_4O^+$, likely acetaldehyde formed via direct charge transfer from O_2^+, was detected in 13 diseased individuals. This ion proved to be a more reliable marker for acetaldehyde than the protonated form $(C_2H_4O)H^+$, which, as discussed in Section 2.1, was confounded by the dominant $(CO_2)H^+$ peak. In addition, several fragment ions, including $(C_3H_2)H^+$, $(C_3H_4)H^+$, and $(C_4H_6)H^+$, showed consistent levels across samples, suggesting potential as supporting markers, although their parent compounds could not be definitively identified.

Table 1. Monoisotopic mass-to-charge ratios (m/z), molecular formulas, and putative identifications for the top 15 ions elevated above the 95% confidence interval of healthy controls. Ions are ranked by the number of diseased individuals exhibiting threshold exceedances. The table also includes the number of samples exceeding across the whole diseased period and their distribution across Days 1–5 post-diagnosis.

m/z	Formula	Possible ID	#Diseased Samples	#Diseased Individuals	D1	D2	D3	D4	D5
58.041	$C_3H_6O^+$	Acetone/propanal *(via O_2^+ charge transfer)*	31	16	7	11	5	4	4
31.018	$(CH_2O)H^+$	Formaldehyde	32	15	8	11	5	4	4
87.08	$(C_5H_{10}O)H^+$	3-methylbutanal, 2-pentanone, Pentanal	27	15	6	11	2	4	4
44.024	$C_2H_4O^+$	*Acetaldehyde (via O_2^+ charge transfer)*	32	14	7	10	7	4	4
56.04427	56.0443	*unknown*	24	14	6	8	4	4	2
61.05348	61.0535	*unknown*	26	14	6	11	2	4	3
39.023	$(C_3H_2)H^+$	Fragment	26	13	6	10	3	4	3
40.02629	40.0261	*unknown*	27	13	6	10	4	4	3
41.03858	$(C_3H_4)H^+$	Fragment	27	13	6	10	3	4	4
53.00223	53.0022	*unknown*	29	13	7	10	6	3	3
55.054	$(C_4H_6)H^+$	Fragment	23	13	5	7	3	4	4
56.057	56.057	*unknown*	23	13	5	7	3	4	4
59.049	$(C_3H_6O)H^+$	Acetone/propanal	26	13	6	10	3	4	3
60.05248	60.0524	*unknown*	26	13	6	10	3	4	3
48.00337	$(CH_4S)H^+$	Methanethiol	23	12	5	8	4	4	2

3.3 Age-Normalised Emission Profiles Across Disease Days (D1 to D5)

All measured emission rates were normalised using their respective growth response curves. The coefficients and confidence intervals associated with these curves are provided for each of the 86 identified ions in Table S1 of the Supplementary Information.

To investigate how breath volatiles change over the course of the five days following disease onset, the normalised data were aggregated (using the median) for both the healthy control group and each day of the diseased period. These results are presented in Figure 3 for Formaldehyde (Panel A), Acetone/Propanal (Panel B), and $(C_5H_{10}O)H+$ ion (Panel C). A Mann-Whitney U test was applied to assess statistical differences between each diseased day and the healthy control population.

All three markers exhibited broadly similar temporal profiles, beginning with statistically significant elevations in formaldehyde and acetone/propanal on Day 1 ($p < 0.01$), while no significant difference was observed for $C_5H_{10}O^+$ at this stage. On Day 2, all compounds showed their largest deviations from healthy controls, with highly significant increases for all three markers ($p < 0.001$). This elevated response persisted into Day 3, where formaldehyde and acetone/propanal remained strongly significant ($p < 0.001$) and $(C_5H_{10}O)H^+$ retained a more modest but still significant difference ($p < 0.05$). By Day 4, formaldehyde and acetone/propanal remained elevated ($p < 0.04$), whereas $(C_5H_{10}O)H^+$ had returned to baseline levels and was no longer significantly different.

The supplementary Information contains similar visualisations for the remaining compounds listed in Table 1 (figures S1-S3).

The temporal profile of VOC emission rates across the 5-day diseased period mirrors the distribution of threshold exceedances observed for the three key compounds. As illustrated in Figure 2, each breath sample is annotated by disease day (1–5), revealing consistent day-wise variation in marker elevation. Among the top three markers, 22% of all threshold exceedances occurred on Day 1, rising sharply to 42% on Day 2, the peak response, before declining to 11%, 15%, and 10% on Days 3, 4, and 5, respectively. These data suggest that the most pronounced perturbations in breath VOCs occur within the first 48 hours following onset of clinical symptoms, with Day 2 consistently representing the strongest signal across individuals.

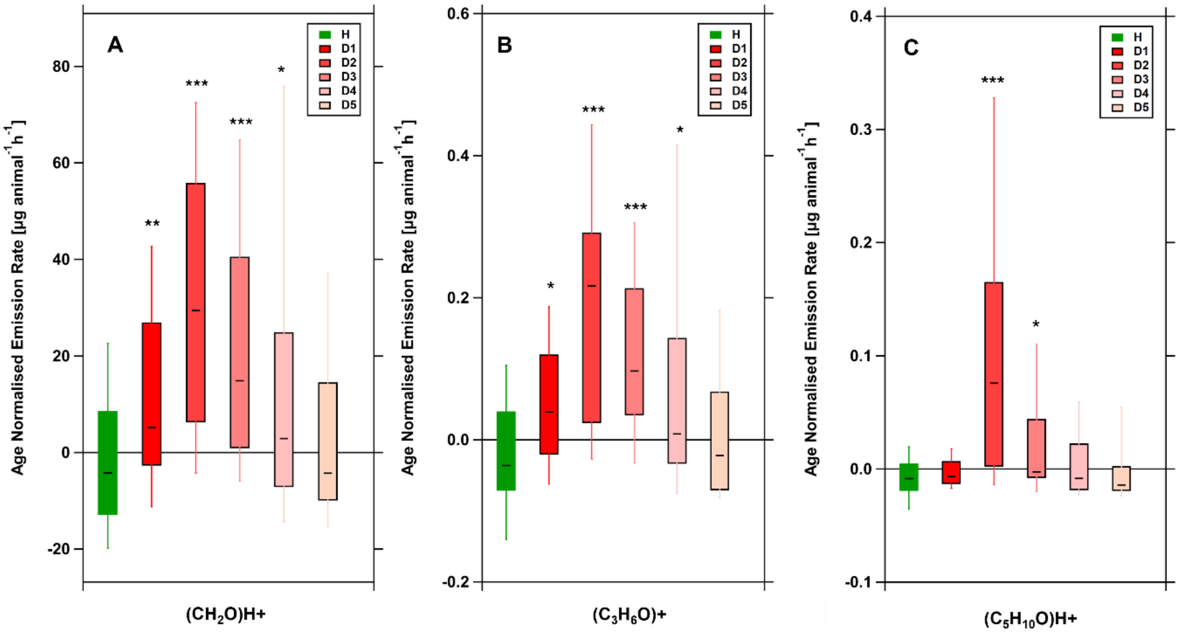

Figure 3. Box-and-whisker plots of age-normalised breath emission rates of formaldehyde (A), acetone/propanal (B), and ($C_5H_{10}O$)H^+ (C) during healthy and diseased periods (progressing from D1 to D5). Asterisks indicate statistical significance: ***p < 0.001, **p < 0.01, *p < 0.05.

3.3.1 Non threshold exceedances

Of the 21 diseased animals, breath marker exceedances were detected in up to 16 individuals. Importantly, these 16 cases represent the complete subset of diseased animals that were detectable using any combination of these or other markers. In other words, there was no additional discriminatory signal in the remaining five BRD-positive individuals that could be recovered through either linear or non-linear combinations of the available markers, when comparing against the healthy population detection thresholds.

Among the five that exhibited no threshold exceedances, further inspection revealed that one animal (#209498) lacked a measurement on Day 2, the time point associated with the highest probability of exceedance events, and thus a key opportunity for detection was missed.

Figure 4 shows the time series data for animal #613315. Grey bars indicate the relative health score, with Day 1 marking the onset of the diseased period. While all three markers peaked on Day 2 and returned to baseline by Day 3, their absolute emission levels remained

below the population-derived threshold (solid line), resulting in no exceedance events. This highlights a key limitation of population-based thresholds and suggests that more sensitive or individualised monitoring strategies may yield higher diagnostic resolution.

In contrast, animal #504941 exhibited no elevation in any of the three key ions during the diseased period and received only a single 1.5 mL dose of Metacam, with no antimicrobial treatment administered. Similarly, animals #109490 and #713323, although clinically diagnosed with respiratory disease, did not meet the criteria for antibiotic treatment. Both animals showed no elevation in $(C_5H_{10}O)H^+$ levels. Modest increases in formaldehyde and acetone/propanal were observed on both Days 1 and 2 for #109490, and on Day 2 for #713323 (see Fig. S4), but in all cases, these remained well below the population threshold. The absence of antimicrobial intervention in these animals suggests that the observed VOC changes reflect a physiological response to disease rather than a pharmacological effect. In contrast, the most pronounced increases in VOC emissions, those exceeding the population threshold, were consistently associated with animals that received antibiotic treatment, raising the possibility that antimicrobial administration may act as a confounding factor in VOC-based disease profiling.

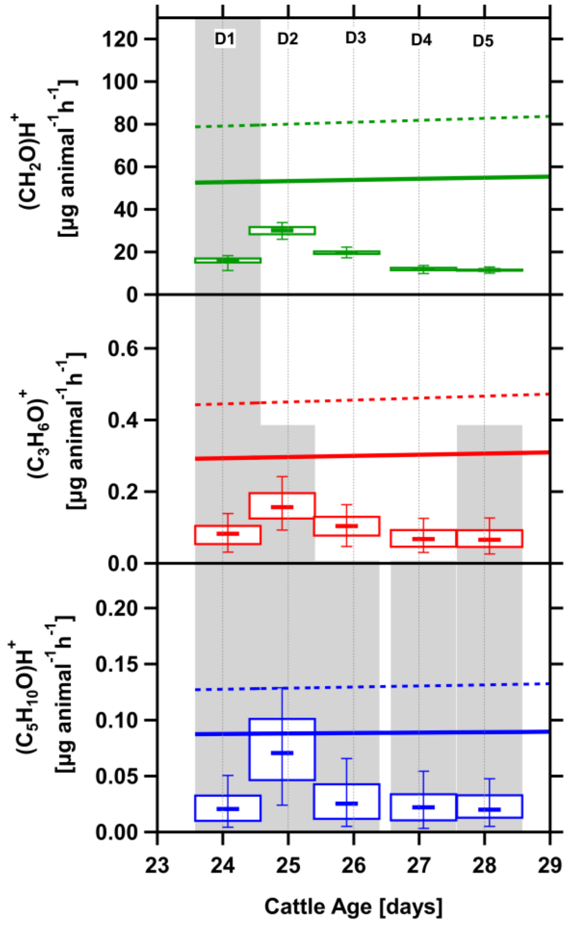

Figure 4. Box-and-whisker plots (10th, 25th, 50th, 75th, and 90th percentiles) of breath emission rates for formaldehyde, acetone/propanal, and the $(C_5H_{10}O)H^+$ ion in calf #613315 across different ages. Grey bars represent the relative health score. D1–D5 denote the five days following disease onset. Solid lines indicate the linear fit of the average breath emission rates for the 'true' healthy cohort, while dashed lines represent the 95% confidence intervals.

3.4 Antibiotic trial

At the onset of the antibiotic trial, three of the 10 calves scored diseased within the first two days and were therefore excluded from the study. Figure 5 presents a principal component analysis (PCA) of breath samples collected from the remaining seven (4 male, 3 female) healthy cattle before and after antimicrobial treatment. Samples obtained on Days 1 and 2, prior to antibiotic administration, cluster together in the lower left quadrant, representing the pre-treatment volatilome profile. Within just one hour of administering LA300 Alamycin, the samples diverge markedly from this baseline, indicating a pronounced shift in breath chemistry. Notably, a gradual return toward the pre-treatment cluster begins around 72 hours post-injection and continues through to 96 hours, suggesting a transient but substantial alteration in the volatilome following treatment.

To further quantify these effects, the three candidate biomarkers identified in the initial trial were re-evaluated in this cohort. After age normalisation, marker emission rates were averaged for each time point to facilitate statistical comparisons between the pre- and post-treatment periods. Figure 6 displays the resulting median normalised emission rates across the group, while individual animal trajectories are provided in the Supplementary Information (Figures S5–S31).

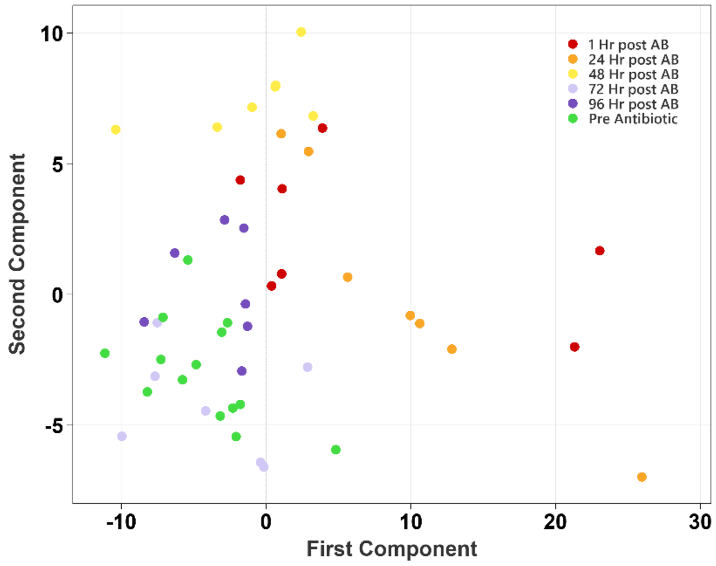

Figure 5. Principal Component Analysis (PCA) of breath samples from seven healthy calves collected prior to treatment (green) and at 1, 24-, 48-, 72-, and 96-hours post-treatment with LA300 Alamycin antibiotic.

Following administration of Alamycin LA 300, a transient increase in sulphur-containing VOCs was observed in the exhaled breath of treated cattle. Notably, dimethyl sulfide (DMS), Figure 6, panel D and methanethiol (not shown), exhibited statistically significant elevations at 1 hour ($p = 0.0159$) and 24 hours ($p = 0.0250$) post-injection, relative to baseline levels established over the two days prior to treatment. These findings were derived using the Mann-Whitney U test across a cohort of seven animals, with emission rates normalised against healthy cohort growth response curves from the initial phase 1 study.

While oxytetracycline, the active compound in Alamycin, is not sulphur-based, the formulation does include sodium formaldehyde sulfoxylate, a sulphur-containing excipient.

Therefore, it is plausible that the observed sulphur-containing VOCs, such as dimethyl sulfide (DMS) and methanethiol, arise from rapid metabolic processing of this compound.

Other VOCs, including $(C_5H_{10}O)H^+$ (Figure 6, Panel C), also showed significant changes at 1 hour (p = 0.0005) and marginal significance at 24 hours (p = 0.0309), but not thereafter. In contrast, compounds such as formaldehyde (Panel A) and acetone/propanal (Panel B) reached significance only at 24 hours, with p-values rising above the 0.05 threshold at later time points.

Crucially, none of the monitored compounds remained significantly elevated beyond 24 hours. This is in marked contrast to findings from the phase 1 study, in which animals were treated while actively diseased. In that context, VOCs such as formaldehyde and acetone/propanal remained significantly elevated for up to 72 hours post-treatment. The absence of sustained VOC elevation in the current study likely reflects the fact that animals were clinically healthy at the time of antibiotic administration. This distinction underscores the influence of underlying disease processes on the breath volatilome and highlights the importance of physiological context when interpreting VOC-based diagnostics.

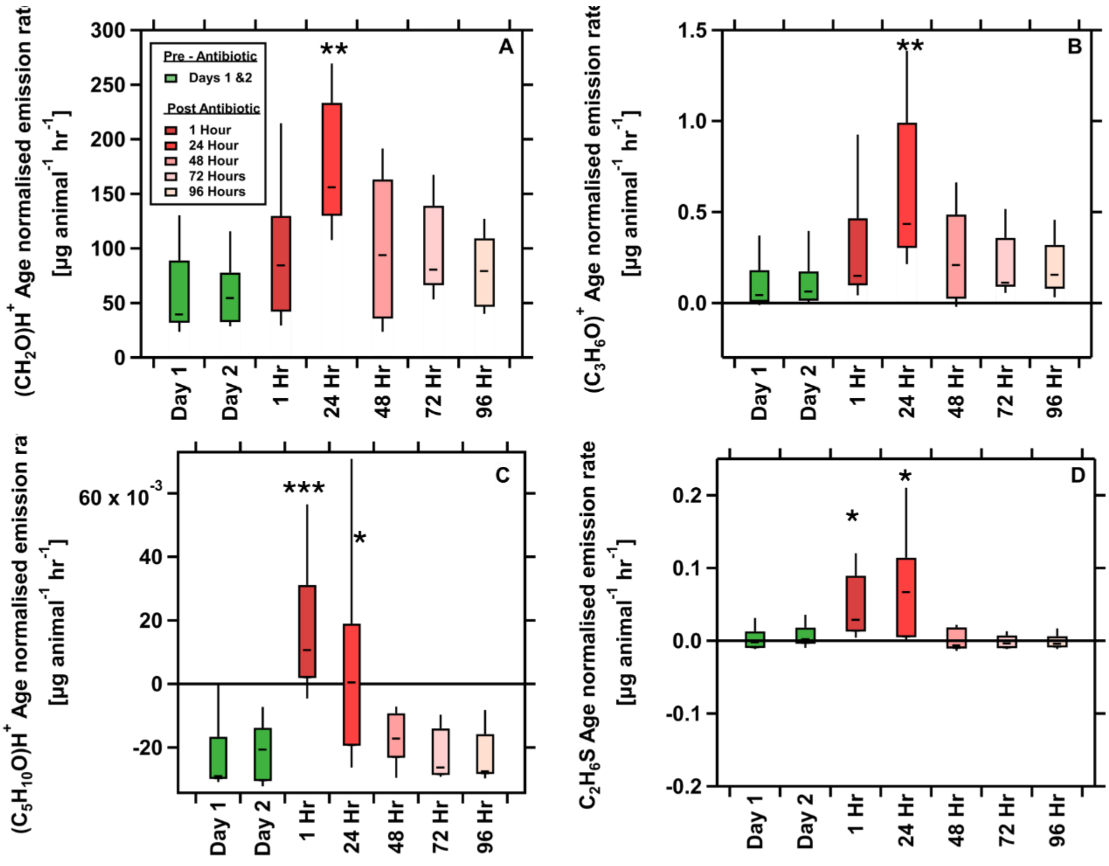

Figure 6 Box and whisker plots showing the emission rates of formaldehyde (A), acetone/propanal (B), $C_5H_{10}O$ ion (C) and dimethyl sulphide (D) measured from the breath of seven healthy cattle before (Days 1 and 2) and after (1, 24, 48, 72, 96 hours) injection with LA300 Alamycin. Emission rates are age normalised by subtracting the growth response curves discussed in Section 3.2. Asterisks indicate statistical significance: ***$p < 0.001$, **$p < 0.01$, *$p < 0.05$.

3.5 Receiver Operating Characteristic Analysis of Key Marker Ions

To evaluate the diagnostic accuracy of the most promising breath biomarkers, Receiver Operating Characteristic (ROC) curves were generated for three ions exhibiting the highest classification potential: $(CH_2O)H^+$ (formaldehyde), $(C_3H_6O)^+$ (acetone/propanal), and $(C_5H_{10}O)H^+$.

ROC curves plot the true positive rate (sensitivity) against the false positive rate (1 – specificity) across a range of detection thresholds, offering a comprehensive view of each biomarker's discriminative ability. Classification performance is quantified using the Area Under the Curve (AUC), where a value of 1.0 indicates perfect discrimination and 0.5 denotes no better than random chance.

To assess variability, bootstrap resampling (n = 1000 iterations) was performed to compute 95% confidence intervals for each ROC curve. These intervals were derived from the 2.5th and 97.5th percentiles of the resampled ROC distributions.

Based on data from the antibiotic trial, ROC analyses shown in figure 7 were conducted separately for breath samples collected on Day 1 (pre-treatment, panels A-C) and Day 2 (post-treatment, Panels D-F) to assess performance changes following antimicrobial exposure.

On Day 1, pre-treatment data showed moderate discriminative power for formaldehyde (CH_2O, m/z 31.018, Panel A) with an AUC of 0.66 (95% CI: 0.53–0.77), and a similar result for acetone/propanal ($C_3H_6O^+$, m/z 58.041, Panel B) with an AUC of 0.65 (0.52–0.77). In contrast, the $(C_5H_{10}O)H^+$ ion (m/z 87.08, Panel C) yielded an AUC of 0.45, indicating no discriminatory ability. This aligns with the absence of statistically significant differences in age-normalised emission rates between healthy calves and those with early-stage disease, confirming that $(C_5H_{10}O)H^+$ is not a biomarker for BRD. It is important to note, however, that animals were sampled at the very first clinical signs of disease, as determined through daily health scoring. This early intervention likely limited the severity and metabolic expression of BRD at the time of sampling. Had the disease been allowed to progress untreated to Day 2, the biomarker signal, particularly for BRD-associated volatiles, may have been further amplified, potentially resulting in higher classification performance. For example, animals #109490 and #713323 both showed larger formaldehyde and acetone/propanal emission values on day 2 of the diseased period, compared to day 1 onset and neither received antibiotic treatment (Figure S4).

By Day 2, after 80% of diseased animals had received antibiotic treatment, the ROC curves (Figure 3, Panels D–F) reflected markedly improved classification for all three ions, with AUCs of 0.82 (0.73–0.94) for formaldehyde, 0.81 (0.73–0.92) for acetone/propanal, and 0.74 (0.60–0.88) for $(C_5H_{10}O)H^+$.

While disease progression could contribute to elevated compound emissions, the data strongly suggest that the enhanced classification performance is a pharmacological effect of antibiotic exposure, rather than a biomarker response to disease status alone.

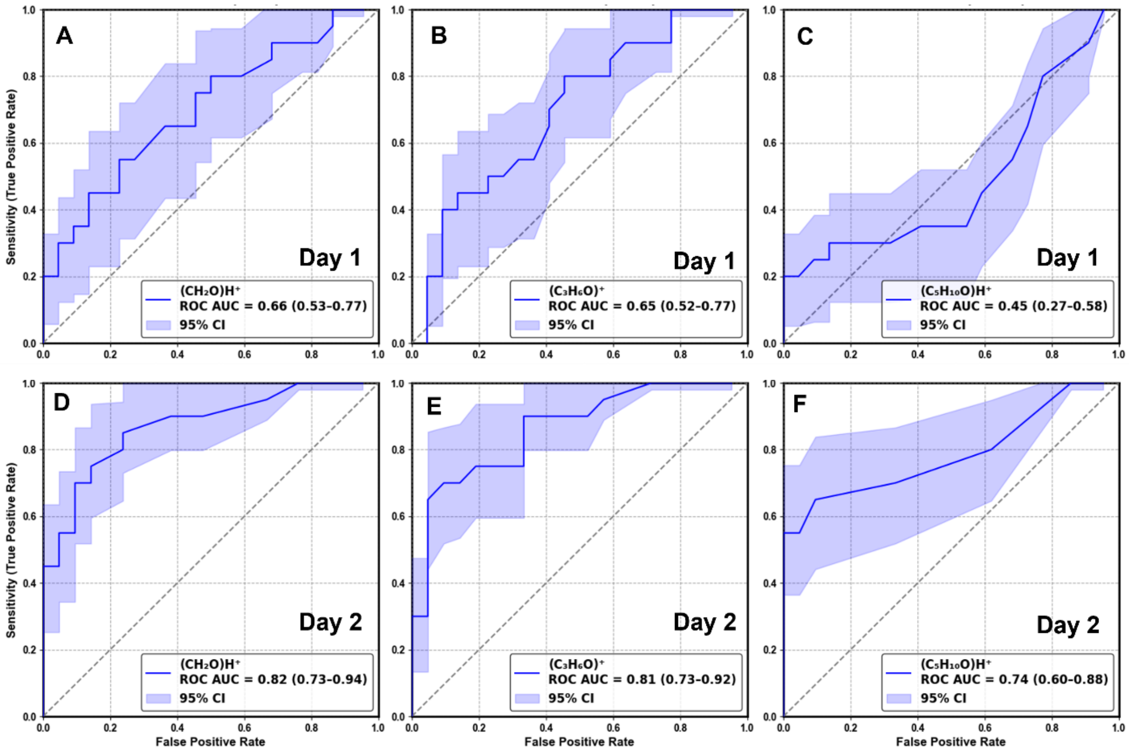

Figure 7. Receiver Operating Characteristic (ROC) curves for the three best-performing ions: formaldehyde ($(CH_2O)H^+$), acetone/propanal ($(C_3H_6O)^+$), and a likely mixture of pentanal, 2-pentanone, and 3-methylbutanal ($(C_5H_{10}O)H^+$). The blue lines represent mean ROC curves across bootstrap iterations, while the shaded bands indicate 95% confidence intervals (CI) derived from 1000 bootstrap resamples. The area under the curve (AUC) is reported for each ion, providing a quantitative measure of its classification performance. Panels A-C show ROC curves for Day 1, pre-treatment and Panels D-F show ROC curves for Day 2, post treatment.

3.6 Low-cost sensors

Two key compounds identified by the PTR-TOF were formaldehyde and acetone/propanal. Each were consistently present in the breath of healthy animals and showed a marked increase in diseased animals at the earliest clinical signs. Importantly, both compounds were among the most abundant VOCs detected, with concentrations ranging from 7 to over 400 ppb. This high abundance is a significant advantage, as it places these biomarkers well within the detection range of lower-cost sensing technologies such as the RoboScientific 720 VOC array. This greatly enhances the feasibility of using such sensors for early, non-invasive on farm disease detection.

Figure 8A illustrates the time series of formaldehyde concentrations measured by the PTR-QiTOF for calf #510923. Alongside this, the peak height from sensor 17 (of 24) on the 720 array is shown. The temporal pattern from the 720 closely aligns with the breath signal detected by the PTR-QiTOF, yielding a correlation coefficient (R^2) of 0.68. An even stronger correlation (R^2 = 0.76) is observed for the desorption slope signal from the same sensor, demonstrating the capability of this specific sensor to broadly capture the dynamics of the formaldehyde signal in breath.

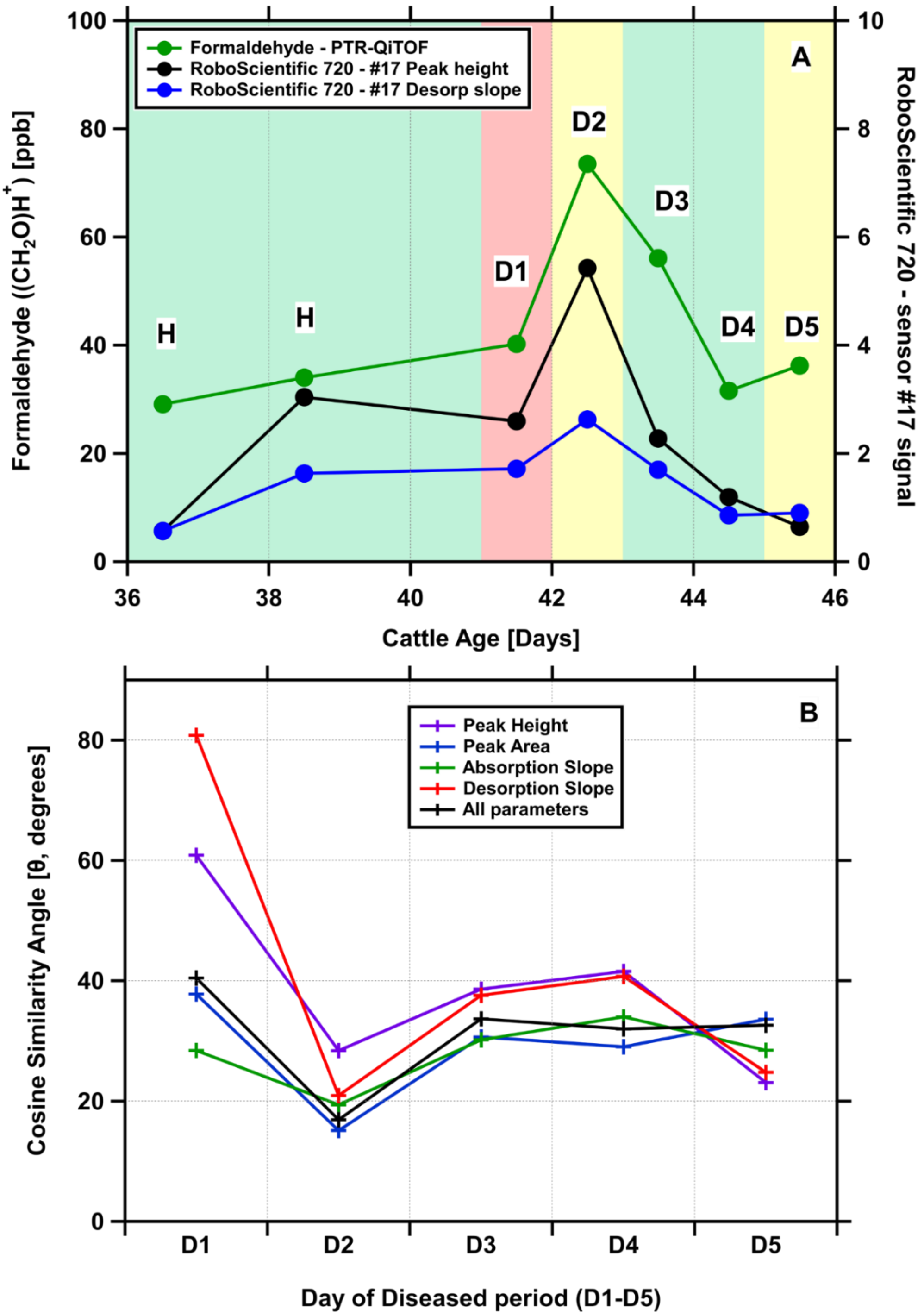

Figure 8A. Formaldehyde concentrations in calf breath measured using PTR-QiTOF, plotted against the animal's age in days. The data span the period before and during the onset of a respiratory disease episode. Also shown are the peak height and desorption slope responses from a single sensor in the RoboScientific 720 sensor array, illustrating sensor behaviour in relation to disease onset. Shaded regions represent the Modified Wisconsin Health Score: red indicates diseased (score ≥ 5), yellow indicates intermediate (score = 4), and green indicates healthy (score ≤ 3). Panel B shows the cosine similarity angle from the 720 on days 1 to 5 of the diseased period compared with the average healthy sample for this calf.

However, this represents the output from just one of 24 distinct sensors contained within the 720, each generating multiple output parameters per breath sample—specifically, peak height, integrated area, absorption slope, and desorption slope. To evaluate how breath profiles diverged from the healthy baseline, cosine similarity analysis was performed multiple times, each using a different subset of these parameters. Similarity was first computed using only peak height values across all sensors, then repeated using only area, absorption slope, and desorption slope, respectively. A final analysis combined all four parameters to generate a composite similarity score. This multi-parameter approach leverages the full dimensionality of the sensor array and may offer enhanced analytical performance by capturing more nuanced changes in breath chemistry than any single parameter or sensor alone.

Figure 8B presents the results of these analyses, showing the angular distance (θ, in degrees) between each diseased-day breath sample and the healthy reference profile for calf #510923. The x-axis represents Days 1 to 5 of the diseased period. A pronounced deviation from the healthy profile is observed on Day 1 across all parameter sets, coinciding with the onset of clinical signs. Following antibiotic administration after the Day 1 sample, a sharp reduction in angular distance is seen on Day 2, suggesting a rapid shift in the VOC profile towards the healthy baseline. This is followed by a modest increase and stabilisation from Days 3 to 5, potentially indicating a new post-treatment baseline. Although some caution is required when analysing results from a single animal.

While each cosine similarity analysis revealed a broadly consistent temporal trend, the greatest magnitude of change was observed in the analysis based solely on peak height data. This suggests that peak height may be particularly sensitive to disease-related shifts in VOC emissions and could serve as a key parameter for early detection.

Interestingly, while the PTR-QiTOF revealed distinct shifts in the breath volatilome following antibiotic treatment, the 720 sensor array showed a marked reduction in angular distance from the healthy baseline. This suggests that antibiotic-induced changes may either be less detectable by the sensor array or may alter the VOC profile in a way that resembles recovery, even if the underlying physiological state has not fully returned to healthy. This highlights the importance of distinguishing between genuine biological recovery and treatment-induced shifts in VOC emissions. Notably, individual sensors within the array, such as sensor #17, were able to reproduce temporal patterns closely aligned with PTR-QiTOF measurements for specific compounds like formaldehyde. However, this reflects just one of 24 sensors, and the overall array response may average out or obscure compound-specific dynamics, especially when using composite similarity metrics.

These findings, though based on a single animal, demonstrate the value of multi-parameter sensor data and suggest that low-cost sensor arrays like the 720 may offer strong potential for early, non-invasive disease detection in field settings.

4. Conclusions

This study demonstrates that antibiotics can significantly confound breath-based disease detection in cattle by rapidly (<1 hour) altering the volatilome in ways that mimic or obscure true disease signals. While moderate diagnostic potential was observed on the first day of clinical signs, prior to antimicrobial administration, the most pronounced elevations in breath markers occurred post-treatment, particularly on Day 2, coinciding with peak pharmacological exposure. ROC analysis confirmed strong classification performance following antibiotic administration; however, data from an independent trial revealed that these discriminative patterns were largely attributable to the drugs themselves rather than underlying pathology.

Among the most responsive compounds prior to treatment
were formaldehyde and acetone/propanal. These VOCs are among the most abundant constituents of exhaled breath, and their elevation during respiratory illness likely reflects increased oxidative stress and lipid peroxidation, processes commonly associated with inflammation and cellular damage in the lungs. Their ubiquity and biological relevance suggest that they may serve as accessible markers of host metabolic response to infection. Importantly, their high baseline abundance also offers promise for the development of low-cost, on-farm diagnostic tools, as their detection may not require the ultra-low limits of detection provided by high-end instruments such as PTR-QiTOF.

Moreover, because these compounds are consistently present in healthy animals, they provide a stable reference point for individualised monitoring, where each animal's breath profile is compared to its own healthy baseline rather than a population average. This approach may improve diagnostic sensitivity by capturing subtle, within-animal changes that would otherwise fall below population-level thresholds. For example, in this study, animals #109490 and #713323 exhibited clear increases in formaldehyde and acetone/propanal concentrations during the early stages of disease, yet these values remained below the population threshold. Such cases underscore the potential of personalised baselines to detect early physiological perturbations that might be missed by population-based screening alone.

However, it is important to acknowledge that these VOCs identified are not unique to BRD. Acetone, for instance, is a well-established biomarker of ketosis, and both acetone and formaldehyde are broadly associated with oxidative stress and systemic inflammation. Their elevation in breath may therefore reflect a general host metabolic response rather than a disease-specific signature. As such, while these markers show promise for detecting physiological perturbations associated with respiratory illness, their diagnostic specificity for BRD remains uncertain.

In contrast, compounds such as $(C_5H_{10}O)H^+$ and dimethyl sulphide (DMS) showed their strongest elevations following antibiotic administration, suggesting a pharmacological rather than pathological origin. The presence of sodium formaldehyde sulphoxylate, a sulphur-containing excipient in the Alamycin formulation, further supports the hypothesis that some sulphur-containing VOCs, particularly DMS, may arise from metabolic processing of the excipient itself, rather than from the active antibiotic or disease-related pathways.

Temporal patterns of marker expression and individual time-series data underscore the risk of misattribution when interpreting breath signatures without accounting for treatment-related effects. While this analysis focused on antibiotic administration, similar confounding could arise from other interventions, including vaccinations or anti-inflammatory treatments. These findings highlight the need for caution when deploying untargeted volatilome analyses for disease diagnostics in medicated animals and suggest that future breath-based diagnostic platforms must explicitly account for pharmacological and immunological influences to avoid spurious classifications.

Importantly, the robust and consistent breath responses to antimicrobial administration observed here also point to a potential secondary application: using breath analysis to monitor treatment administration, compliance, or pharmacodynamic response in livestock. The rapid onset and reproducibility of VOC changes following oxytetracycline injection suggest that breath profiling could offer a non-invasive means of tracking drug exposure in real time. With further development, this approach may support precision dosing strategies, reduce unnecessary antibiotic use, and improve treatment outcomes.

Acknowledgements

This work was funded as part of the Innovate UK grant (Project # 10072590): DETECT.

Data Availability

The dataset supporting this study is freely available from the Zenodo data repository at 10.5281/zenodo.17940805

Author Contributions

Ben Langford: Conceptualisation, Funding Acquisition, Methodology, Investigation, Formal Analysis, Visualisation, Writing – original draft, Writing – review & editing

Johanna Brans: Investigation, Writing – review & editing

Claire Broadbent: Investigation, Writing – review & editing

Tim Gibson: Investigation, Writing -review & editing

Mark Hanlon: Methodology, Writing -review & editing

Marie Haskell: Conceptualisation, Funding Acquisition, Methodology, Writing – original draft, Writing – review & editing

Laura Nicoll: Investigation, Writing – review & editing

Neil Mullinger: Methodology, Writing – review & editing

Carol-Anne Duthie: Conceptualisation, Funding Acquisition, Methodology, Writing – original draft, Writing – review & editing

References

1. Ibrahim, W., et al., *Breathomics for the clinician: the use of volatile organic compounds in respiratory diseases.* Thorax, 2021. **76**(5): p. 514-521.
2. van der Schee, M.P., et al., *Breathomics in Lung Disease.* Chest, 2015. **147**(1): p. 224-231.
3. Ghosh, C., et al., *Breath-Based Diagnosis of Infectious Diseases: A Review of the Current Landscape.* Clin Lab Med, 2021. **41**(2): p. 185-202.
4. Li, W., et al., *A cross-sectional study of breath acetone based on diabetic metabolic disorders.* Journal of Breath Research, 2015. **9**(1): p. 016005.
5. Krilaviciute, A., et al., *Detection of cancer through exhaled breath: a systematic review.* Oncotarget, 2015. **6**(36): p. 38643-57.
6. Wyse, C.A., et al., *Current and future uses of breath analysis as a diagnostic tool.* Veterinary Record, 2004. **154**(12): p. 353-360.
7. Haddadi, S., J.A. Koziel, and T.J. Engelken, *Analytical approaches for detection of breath VOC biomarkers of cattle diseases -A review.* Analytica Chimica Acta, 2022. **1206**: p. 339565.
8. Weber, M., et al., *Detection of Paratuberculosis in Dairy Herds by Analyzing the Scent of Feces, Alveolar Gas, and Stable Air.* Molecules, 2021. **26**(10): p. 2854.
9. Dobbelaar, P., et al., *Detection of ketosis in dairy cows by analysis of exhaled breath.* Veterinary Quarterly, 1996. **18**(4): p. 151-152.
10. Mottram, T.T., et al., *An experiment to determine the feasibility of automatically detecting hyperketonaemia in dairy cows.* Livestock Production Science, 1999. **61**(1): p. 7-11.
11. Darlington, B., *Agscent Cow Breath Sampling: pregnancy diagnosis proof of concept*. 2021, Meat & Livestock Australia.

12. Gaudino, M., et al., *Understanding the mechanisms of viral and bacterial coinfections in bovine respiratory disease: a comprehensive literature review of experimental evidence.* Vet Res, 2022. **53**(1): p. 70.
13. Dubrovsky, S.A., et al., *Bovine respiratory disease (BRD) cause-specific and overall mortality in preweaned calves on California dairies: The BRD 10K study.* Journal of Dairy Science, 2019. **102**(8): p. 7320-7328.
14. Lindley, G., et al., *Development and Progression of Bovine Respiratory Disease Measured Using Clinical Respiratory Scoring and Thoracic Ultrasonography in Preweaned Calves on Dairy Farms in the United Kingdom: A Prospective Cohort Study.* Animals (Basel), 2025. **15**(3).
15. Delabouglise, A., et al., *Linking disease epidemiology and livestock productivity: The case of bovine respiratory disease in France.* PLOS ONE, 2017. **12**(12): p. e0189090.
16. Dubrovsky, S.A., et al., *Preweaning cost of bovine respiratory disease (BRD) and cost-benefit of implementation of preventative measures in calves on California dairies: The BRD 10K study.* Journal of Dairy Science, 2020. **103**(2): p. 1583-1597.
17. Bushby, E.V., et al., *Early detection of bovine respiratory disease in pre-weaned dairy calves using sensor based feeding, movement, and social behavioural data.* Scientific Reports, 2024. **14**(1): p. 9737.
18. Buczinski, S., D. Achard, and E. Timsit, *Effects of calfhood respiratory disease on health and performance of dairy cattle: A systematic review and meta-analysis.* Journal of Dairy Science, 2021. **104**(7): p. 8214-8227.
19. Peled, N., et al., *Detection of volatile organic compounds in cattle naturally infected with Mycobacterium bovis.* Sensors and Actuators B: Chemical, 2012. **171-172**: p. 588-594.
20. Langford, B., et al., *Passive breath monitoring of livestock: using factor analysis to deconvolve the cattle shed.* Journal of Breath Research, 2022. **16**(2): p. 026005.
21. Juge, A.E., et al., *Using Canine Olfaction to Detect Bovine Respiratory Disease: A Pilot Study.* Frontiers in Veterinary Science, 2022. **Volume 9 - 2022**.
22. Kuchmenko, T., et al., *Portable Electronic Nose for Analyzing the Smell of Nasal Secretions in Calves: Toward Noninvasive Diagnosis of Infectious Bronchopneumonia.* Vet Sci, 2021. **8**(5).
23. Schelkopf, C.S., et al., *Cyranose® 320 eNose Effectively Differentiates Pre- and Post-Challenge Respiratory Samples in an Induced Bovine Respiratory Disease Model.* Veterinary Sciences, 2025. **12**(6): p. 578.
24. Masebo, N.T., et al., *Evaluation of antimicrobial and non-steroidal anti-inflammatory treatments for BRD on health and welfare in fattening bulls: a cross-sectional study.* Veterinary Quarterly, 2024. **44**(1): p. 1-11.
25. Escalona, J., et al., *Beta-Lactam Antibiotics Can Be Measured in the Exhaled Breath Condensate in Mechanically Ventilated Patients: A Pilot Study.* J Pers Med, 2023. **13**(7).
26. Sulzer, P., et al., *A Proton Transfer Reaction-Quadrupole interface Time-Of-Flight Mass Spectrometer (PTR-QiTOF): High speed due to extreme sensitivity.* International Journal of Mass Spectrometry, 2014. **368**: p. 1-5.
27. Steinbacher, M., et al., *Performance characteristics of a proton-transfer-reaction mass spectrometer (PTR-MS) derived from laboratory and field measurements.* International Journal of Mass Spectrometry, 2004. **239**(2-3): p. 117-128.
28. Taipale, R., et al., *Technical Note: Quantitative long-term measurements of VOC concentrations by PTR-MS - measurement, calibration, and volume mixing ratio calculation methods.* Atmospheric Chemistry and Physics, 2008. **8**(22): p. 6681-6698.
29. Li, M., et al., *Human metabolic emissions of carbon dioxide and methane and their implications for carbon emissions.* Science of The Total Environment, 2022. **833**: p. 155241.
30. Dißmann, L., et al., *Evaluation of a Respiration Rate Sensor for Recording Tidal Volume in Calves under Field Conditions.* Sensors, 2023. **23**(10): p. 4683.
31. Langford, B., et al., *Early-Life Respiratory Emissions of CO_2, CH_4, and N_2O in pre-weaned Dairy-bred Calves.* Journal of Sustainable Agriculture and Environment, 2026.

32. McGuirk, S.M. and S.F. Peek, *Timely diagnosis of dairy calf respiratory disease using a standardized scoring system.* Anim Health Res Rev, 2014. **15**(2): p. 145-7.

Click here to access/download
Supporting Information
Langford_et_al_PlosOne_2026_SI.docx
