# Supplementary material for: Antibiotics confound breath-based respiratory disease detection in calves
Source: PLoS One. 2026 Jun 24;21(6):e0351838. doi: 10.1371/journal.pone.0351838 (PMC13293443; doi:10.1371/journal.pone.0351838)
Supplement: Attachment — Submitted filename: Response.docx [file pone.0351838.s005.docx]

**We thank the reviewers for the time taken to assess our manuscript and for the constructive suggestions offered. Below we provide a detailed, point‑by‑point response. Reviewer comments are shown in *italics* and our responses follow in bold.**

*Reviewer #1: The authors measured several volatile organic compounds (VOCs) in exhaled breath using a PRT-Qi-TOF and volatile gas analyzers. Healthy and suspected ill animals were categorized using a modified Wisconsin scoring system. The VOC results showed the levels in breath depend on calf age, time of collection, and health status. Several of these VOCs appeared to be affected by antibiotic administration. Since antibiotic administration is common when animals are suspected of respiratory disease, this constitutes a confounder for BRD diagnosis using VOCs. This finding is significant as it may hinder the applicability of VOC analysis for BRD diagnosis. In general, the data is of acceptable quality with relevant statistical analyses. Results are organized into understandable figures and tables. Though many control measurements were performed, additional are needed to support the validity of sulfur-containing VOCs as BRD biomarkers.*

**AR: We thank the reviewer for this comment and for recognising the importance of our findings. We would like to clarify a key point: we do not present sulphur‑containing VOCs (e.g., dimethyl sulphide) as BRD biomarkers. Our disease‑associated VOCs are formaldehyde and acetone/propanal, neither contain sulphur. These compounds were both significantly elevated on day 1 of the disease period, prior to antibiotics being administered (figure 3). Sulphur‑containing VOCs are discussed specifically in Section 3.4 (antibiotic trial), where we state that they arise only after antibiotic administration and are plausibly derived from sodium formaldehyde sulfoxylate, a sulphur‑containing excipient in Alamycin LA 300.**

**For clarity, we have reworded the abstract to remove any ambiguity in our reporting: *“****We identified several candidate ions, including formaldehyde and acetone/propanal, that showed significant elevation at the onset of disease and a modest detection capability (AUC = 0.53-0.77). However, the strongest discrimination between diseased and controls occurred after antibiotic administration, suggesting a confounding pharmacological influence. To isolate this effect, a secondary trial was conducted in which healthy calves were treated with oxytetracycline dihydrate (Alamycin® LA 300). This revealed rapid and transient changes in the breath volatilome, with significant increases in dimethyl sulphide and (C₅H₁₀O)H^+^ within 1 hour, and other VOCs, including formaldehyde and acetone/propanal within 24 hours of treatment. These findings demonstrate that antibiotics can substantially alter breath VOC profiles, potentially mimicking or masking disease signals.”*

*Reviewer #1: However, the text associated with these results is very difficult to understand. This significantly hinders the readability of the manuscript and needs to be heavily revised. There are grammatical and formatting errors throughout the text that need to be addressed. The manuscript is poorly cited. Relevant research exists in the literature that the authors need to include in the introduction and results sections of the manuscript. Furthermore, the authors need to be judicious when citing to ensure in-text claims are truly supported by literature. Several references that the authors cited do not include the content they claim. The manuscript presents important results that contribute to the field of exhaled breath analysis and BRD diagnosis. However, the manuscript preparation hinders the communication of the findings.*

**AR: We appreciate the reviewer’s concern. We have carefully re‑read the entire manuscript and implemented targeted improvements to clarity and flow (particularly within the Method and the Conclusions – specifics are outlined in later responses). We have also revised citations so each claim is supported by the most appropriate literature; where prior references did not align exactly (e.g., economic impact / management burden), we replaced them with more appropriate citations.**

*Reviewer #1: Positives:*

*• Measuring four replicate blanks followed by four replicate samples over two minutes is a good approach to increase confidence in the results. Similar benefit for Figure 6, where the four volatiles were measured for two days before antibiotic administration.
• The data are well summarized in the figures. The plots are well organized with color coding that helps with visualization.*

**AR: We thank the reviewer for these positive comments.**

*Reviewer #1: Criticisms:*

*• Add a diagram of the experimental procedure, including the online breath sampling setup for PTR-QiTOF, MIRO MGA10-GP, and 720 VOC Analyzer. This helps readers visualize the breath collection process.*

**AR: This is an excellent suggestion (also raised by Reviewer #2). We have now added a schematic diagram showing the mask, sampling lines, PTR‑QiTOF / MIRO / Model 720. We placed this in the Supplementary Information and refer to it in Methods.**

**“***Figure 1. Experimental setup comprising a mobile laboratory housing the gas analysers and a 6 m heated sample line connected to a custom‑made breath‑sampling mask. Data acquisition was operated by trained farm technicians, who initiated sampling from the side of the cattle pen.***”**

*Reviewer #1: Define the temperature units used (e.g. °C)*

**AR: All temperatures are now standardised to °C throughout.**

*Reviewer #1: Clarify the methodology throughout section 2. The procedures are difficult to understand. This is especially true for section 2.4, though not exclusively.*

**AR: Without specific feedback on what sections required further clarity it is difficult to offer targeted improvements. As Section 2.4 was mentioned we have revised this whole section in an attempt to improve clarity on what was done, also incorporating suggestions from reviewer 2 regarding the placement of the ethical statement, cattle ages and administered medications.**

***“2.4 Animals, Health Assessment and Treatments***

*The study was conducted under ethical approval from SRUC’s Animal Experiments Committee (Approval number: AEX 2024-024 DAI).*

*A total of 65 calves completed the trial, aged between 12 and 86 days, comprising 30 heifers and 35 steers. Each animal was born to a Holstein dam—a dairy breed—but sired by bulls representing three beef breeds: Hereford, Aberdeen Angus, and British Blue[36].*

*During the trial animals were fed 4 litres of milk replacer (Carr’s Billington Vitality calf milk) twice daily at 08:00 and 15:00 and were given ad libitum access to barley straw and starter pellets (Davidson’s rapid starter pellets, 2359). Health status was assessed each morning using the modified Wisconsin scoring system[37], which grades five clinical indicators—rectal temperature, nasal discharge, eye discharge, ear droop, and cough—on a scale of 0–3. For the purposes of this study, a calf was classified as diseased only when both the cumulative score reached five or more and rectal temperature exceeded 39.5 °C. This combined threshold was used to reduce misclassification arising from transient environmental irritants or mild non-respiratory conditions that can elevate individual components of the clinical score. Because the calves were group‑housed, faecal consistency was not included, as faeces could not reliably be attributed to specific individuals. Animals identified as either healthy controls or meeting the criteria for respiratory disease during the morning assessment were sampled later the same day, following the breath‑sampling protocol outlined below.*

*The number of breath samples that could be collected from each animal was determined by the conditions of the Home Office licence governing the study. Each calf could contribute up to five healthy breath samples throughout the trial period. If an animal met the disease criteria, up to five further breath samples could be taken on consecutive days following disease onset, with a maximum of two such disease episodes sampled per calf. These regulatory constraints dictated the feasible sampling frequency for each individual and explain why the number of available breath measurements varied across animals.*

*Immediately after breath sampling, calves diagnosed with respiratory disease were treated according to standard farm practice and all treatment histories are reported in the associated data publication.* *Treatment typically consisted of an intramuscular injection of meloxicam (Metacam®, Boehringer Ingelheim) alongside oxytetracycline dihydrate (Alamycin® LA 300, Norbrook Laboratories), administered into the gluteal muscles of the hindquarters. In a small number of cases where clinical signs did not improve sufficiently, further treatment with gamithromycin (Zactran®, Boehringer Ingelheim) or amoxicillin trihydrate–clavulanic acid (Synulox®, Zoetis) was provided. Calves were not vaccinated prior to the study, and polymerase chain reaction (PCR) testing was not performed, meaning that the specific pathogens responsible for each disease event could not be identified. Consequently, some respiratory episodes may have involved viral, bacterial, or mixed infections, or systemic conditions that mimicked BRD. This reflects the reality of on‑farm diagnosis and mirrors the context in which breath‑based disease detection technologies would ultimately be deployed.****”***

*Reviewer #1: Figure 1 x-axis is confusing. At first glance, it appears to be fractions of a minute until reading the caption. Please clarify this.*

**AR: We have reformatted the x‑axis to “Time (minutes)” with ticks from 0–6.**

*Reviewer #1: Listing the full calf number designations is confusing and hard to follow. Please simply the assignments for each animal.*

**AR: We appreciate the reviewers concern, but in our view reporting full animal IDs is essential to support traceability to the already openly archived dataset, allowing readers to cross‑reference figures with data files that use the same IDs.**

*Reviewer #1: In the text, explain why there are more breath samples in the pre-antibiotic group compared to the seven healthy calves treated with antibiotics in the Figure 5 PCA analysis.*

**AR: We have adjusted the figure 5 caption to explain the larger number of pre-antibiotic samples:**

**“***Figure 5. Principal Component Analysis (PCA) of breath samples from seven healthy calves collected prior to treatment (green) and at 1, 24-, 48-, 72-, and 96-hours post-treatment with LA300 Alamycin antibiotic. Pre‑treatment samples were collected across two days, resulting in 14 data points for that group, whereas all post‑treatment time points contain seven samples each, representing single sampling events.”*

*Reviewer #1: Clarify the p-values for both DMS and methanethiol. In the text, only one p-value is listed at the two listed time points (i.e., 1 h & 24 h). For two compounds, this should be four total p-values (2 compounds × 2 time points).*

**AR: We have removed references to methanethiol to focus only on those compounds shown in the figures (e.g. DMS). The p-values therefore remain unchanged.**

*Reviewer #1: Since DMS is proposed as an important exhaled VOC, the authors need to provide additional evidence that the DMS does not originate from formaldehyde sulfoxylate. At present, the authors only state that it is “plausible” that the sulfur-containing VOCs originate from the antibiotic formulation*.

**AR: We believe there is a misunderstanding. DMS is not proposed as a BRD biomarker in our manuscript. DMS is discussed only in the context of the antibiotic trial, where the data show that DMS increases rapidly and significantly within hours of Alamycin administration and is not elevated in diseased calves that did not receive antibiotics. Given that the Alamycin formulation contains sodium formaldehyde sulfoxylate, a sulphur‑donor excipient, the observed timing and treatment‑dependence together support that the DMS signal originates from the formaldehyde‑sulfoxylate excipient, not from disease. This interpretation is already explicit in the Results and Conclusions.**

*Reviewer #1: Though the findings in Section 3.6 and Figure 8 are interesting, they are preliminary. The data expand the scope of the paper beyond what is appropriate. It could be included in a follow-up paper, if desired, with additional subjects.*

**AR: We appreciate the perspective but respectively disagree. We retain this section because the Introduction explicitly motivates the relevance of low‑cost sensors for on‑farm diagnostics; demonstrating, even preliminarily, that sensor outputs track key VOC dynamics (including antibiotic‑related artefacts) is directly aligned with the paper’s aims.**

**We have added the following to the introduction to further strengthen the connection: *“****However, it must be ensured that reliable and disease-specific breath signatures can be identified* ***and that these low-cost instruments can also detect them.”***

***And added the following to the last paragraph:* “***In this study, we investigate breath-based detection of BRD within a farm setting where disease management involved antibiotic treatment.* ***Using online mass spectrometry, we*** *analysed breath samples from 65 calves to characterise VOC patterns in diseased and healthy animals, assessing the potential for breath-based disease detection, including piloting the use of low-cost sensors* ***to determine whether they are sufficiently sensitive to any identified markers of disease (phase 1)****. To further probe the influence of antibiotics, we conducted a secondary trial (phase 2) administering treatment to healthy calves, tracking breath composition over multiple time points.”*

*Reviewer #1: Throughout the paper, the wording is confusing and hard to follow. Many paragraphs are wordy and the conclusions are not clear. This needs to be rectified.*

**AR: We note that Reviewer #2 explicitly answered *“Yes”* when asked whether the submitted article was clear, correct, and unambiguous, indicating that concerns about clarity are not shared across reviewers. We have nonetheless carefully re‑read the full manuscript and corrected several minor typographical issues. As mentioned above, we have also revised the section referred to as “2.4” to improve logical ordering and flow.**

**As the conclusions are mentioned we have reviewed and revised this section to improve clarity, with major changes highlighted as bold text below.**

**We would be happy to address any additional issues if the reviewer can provide more specific examples or indicate particular areas of concern.**

***“******4. Conclusions***

*This study demonstrates that antibiotic treatment can significantly confound breath-based disease detection in cattle by rapidly (<1 hour) altering the volatilome in ways that mimic or obscure true disease signals****. Although several VOCs increased in the days following the onset of clinical signs, only formaldehyde and acetone/propanal (and several fragment ions) were statistically elevated at the onset of clinical symptoms, prior to antibiotic administration. The strongest increases in breath markers occurred after treatment, particularly on day 2, coinciding with peak drug exposure. ROC analysis confirmed high classification performance at this time point. However, an independent trial in healthy calves showed that these discriminative patterns were largely driven by the antibiotic itself rather than the underlying pathology.***

*The most responsive pre-treatment compounds in diseased cattle were formaldehyde and acetone/propanal. These VOCs are among the most abundant constituents of exhaled breath, and their elevation during respiratory illness likely reflects increased oxidative stress and lipid peroxidation, processes commonly associated with inflammation and cellular damage in the lungs. Their ubiquity and biological relevance suggest that they may serve as accessible markers of host metabolic response to infection. Importantly, their high baseline abundance also offers promise for the development of low-cost, on-farm diagnostic tools, as their detection may not require the ultra-low limits of detection provided by high-end instruments such as PTR-QiTOF.*

*Moreover, because these compounds are consistently present in healthy animals, they provide a stable reference point for personalised monitoring, where each animal’s breath profile is compared to its own healthy baseline rather than a population average. This approach may improve diagnostic sensitivity by capturing subtle, within-animal changes that would otherwise fall below population-level thresholds. For example, in this study, animals #109490 and #713323 exhibited clear increases in formaldehyde and acetone/propanal concentrations during the early stages of disease, yet these values remained below the population threshold. Such cases underscore the potential of personalised baselines to detect early physiological perturbations that might be missed by population-based screening alone.*

*However, it is important to acknowledge that these VOCs are not specific to BRD.* ***Acetone, for instance, is a well-established biomarker of ketosis, and short‑term reductions in feed intake during illness can induce negative energy balance and raise breath acetone independently of respiratory pathology. More broadly, both acetone and formaldehyde are associated with oxidative stress and systemic inflammation.*** *Their elevation in breath may therefore reflect a general host metabolic response rather than a disease-specific signature. As such, while these markers show promise for detecting physiological perturbations associated with respiratory illness, their diagnostic specificity for BRD remains uncertain.*

*In contrast, compounds such as (C₅H₁₀O)H⁺ and DMS showed their strongest elevations following antibiotic administration, suggesting a pharmacological rather than pathological origin. The presence of sodium formaldehyde sulphoxylate, a sulphur-containing excipient in the Alamycin formulation, further supports the hypothesis that some sulphur-containing VOCs, particularly DMS, may arise from metabolic processing of the excipient itself, rather than from the active antibiotic or disease-related pathways.* ***Importantly, the antibiotics also amplified the very markers that initially appeared disease‑associated: both formaldehyde and acetone/propanal reached their highest concentrations after treatment in both trials. This demonstrates a dual confounding effect, whereby medication elevates additional VOCs while simultaneously inflating genuine disease‑linked markers.***

***Therefore, deviations from an animal’s baseline volatilome should not be assumed to indicate disease unless potential confounders, such as recent medication or other interventions, have been ruled out, as these can generate similar shifts in VOCs.*** *Although this analysis focused on antibiotic administration, similar confounding could occur following other interventions, including vaccinations or anti-inflammatory treatments. These findings highlight the need for caution when deploying untargeted volatilome analyses for disease diagnostics in medicated animals and suggest that future breath-based diagnostic platforms must explicitly account for pharmacological and immunological influences to avoid spurious classifications.*

*Importantly, the robust and consistent breath responses to oxytetracycline dihydrate (Alamycin® LA 300) observed here also point to a potential secondary application: using breath analysis to monitor treatment administration, compliance, or pharmacodynamic response in livestock. The rapid onset and reproducibility of VOC changes following injection suggest that breath profiling could offer a non-invasive means of tracking drug exposure in real time. With further development, this approach may support precision dosing strategies, reduce unnecessary antibiotic use, and improve treatment outcomes.”*

*Reviewer #1: Comments on citations:
• There are more recent original research and literature reviews discussing exhaled breath analysis for disease diagnosis in both humans and animals. Such recent works should be included in the first paragraph of the introduction.*

**AR: Thank you -we have expanded this section to discuss several more recent reviews in both human and animal breathomics – if there are further specific references the reviewer has in mind we would be happy to accommodate.**

*“Recent reviews continue to highlight the rapid progress in breathomics and its expanding diagnostic potential in both human and veterinary medicine, including advances in GC‑MS and PTR‑MS breath profiling, electronic‑nose technologies, nanosensors, and wearable VOC detection systems [Mortazavi et al., 2025; Wang & Davis, 2024; Bajo‑Fernández et al., 2024; IEEE Xplore, 2024; Taha et al., 2025]. These developments underline the growing feasibility of using exhaled VOCs for early disease detection and real‑time monitoring across a wide range of pathological conditions.”*

**Mortazavi, S., et al., *Exhaled Breath Analysis (EBA): A Comprehensive Review of Non-Invasive Diagnostic Techniques for Disease Detection.* Photonics, 2025. 12(9): p. 848.**

**Wang, R. and M.D. Davis, *A Concise Review of Exhaled Breath Testing for Respiratory Clinicians and Researchers.* Respiratory Care, 2024. 69(5): p. 613-620.**

**Bajo-Fernández, M., et al., *GC-MS-based metabolomics of volatile organic compounds in exhaled breath: applications in health and disease. A review.* Frontiers in Molecular Biosciences, 2024. Volume 10 - 2023.**

**Taha, B.A., et al., *Nanosensors Based on Breathomics for Human Disease Diagnosis: a New Frontier in Personalized Healthcare.* BioNanoScience, 2025. 15(2): p. 268.**

*Reviewer #1: The content in citations 12 and 13 do not match the text with which they are associated. Specifically, there is no discussion of the economic impact of BRD in the listed sources. The authors need to support this claim with other sources. Citations 12 and 13 are important pieces of literature. It would be good to include them elsewhere in the introduction.*

**AR: Thank you for spotting this oversight. We have replaced refs 12 (Gaudino et al) and 13 (Dubrovsky et al) as follows:**

*“Given its promise, breath-based diagnostics could play a transformative role in the detection of bovine respiratory disease (BRD), one of the most economically significant illnesses affecting cattle worldwide[16]”*

[16] O’Donoghue, S., et al., *A Comprehensive Review: Bovine Respiratory Disease, Current Insights into Epidemiology, Diagnostic Challenges, and Vaccination.* Veterinary Sciences, 2025. **12**(8): p. 778.

**As the reviewer points out, Guadino et al and Dubrovsky et al are important pieces of work and we retain Gaudino et al (18) here:**

“*BRD is a complex syndrome, initiated by viral infections that predispose young calves to secondary bacterial invasion, leading to pneumonia and impaired respiratory function[17] [18]*”

**And Dubrovsky (20), here:** *“With calf pneumonia affecting 20–30% of herds in the UK[17], the economic and welfare implications are substantial[19] and include reduced growth rates, increased veterinary costs [20], and elevated mortality risk in pre‑weaned calves.[20-23]”*

*Reviewer #1: Citation 17 does not discuss how calf pneumonia creates “management burdens”. The authors need to (i) reference a more appropriate source and/or (ii) to be more specific with the claim in the text.*

**AR: We have removed references to management burdens, replacing with elevated mortality risk:**

**“***With calf pneumonia affecting 20–30% of herds in the UK[17], the economic and welfare implications are substantial[19] and include reduced growth rates, increased veterinary costs [20], and elevated mortality risk in pre‑weaned calves.”*

*Reviewer #2: This manuscript is an interesting analysis of the effects of antimicrobial therapy on breath VOCs in calves affected with BRD. Please see the attached PDF of your manuscript with my comments.*

***AR: Thank you for the comments – we have extracted the points from the PDF and listed individually below.***

*Reviewer #2: Some additional comments: it may be useful to include a visual diagram of the experiment, showing sampling times.*

***AR: Thank you for the suggestion. We have added this as Figure 1 in the revised manuscript.***

*Reviewer #2: Comment on supplemental figures: animals are referred to as cows in all figure captions, please change this to "calf."*

**AR: This has been changed**

*Reviewer #2: How old are the calves? / State the numbers of males and females*

**AR: The text has been changed accordingly: “***A total of 65 calves completed the trial, aged between 12 and 86 days, comprising 30 heifers and 35 steers. Each animal was born to a Holstein dam—a dairy breed—but sired by bulls representing three beef breeds: Hereford, Aberdeen Angus, and British Blue.”*

*Reviewer #2: use Recommended International Non-Proprietary Name (generic) drug name, dose, and frequency give with trade name and manufacturer in parentheses behind.*

**AR: This has been changed.**

*Reviewer #2: This (ethics statement) should be moved to the top of section 2.5*

**AR: Thank you – this has been moved accordingly.**

*Reviewer #2: where was it given IM? and was it given only once?*

**AR: We revise this section as follows: “***Treatment typically consisted of an intramuscular injection of meloxicam (Metacam®, Boehringer Ingelheim) alongside oxytetracycline dihydrate (Alamycin® LA 300, Norbrook Laboratories), administered into the gluteal muscles of the hindquarters.****”***

*Reviewer #2: how many were treated for BRD?*

**AR: All treatment records, including medications and doses are captured in the associated data publication. A statement to that effect has been added to the revised manuscript.**

*Reviewer #2: Should have a label above for day. unit labels are needed*

**AR: We now make it clear that these are # of exceedances per day of the disease period.**

*Reviewer #2: What is the explanation for this? What are these unknown compounds - I feel like this doesn't give me a lot of information*

**AR: PTR‑QiTOF instruments detect compounds as protonated monoisotopic masses (m/z values). From these exact masses, we attempt to derive a molecular formula by matching combinations of elements (C, H, O, N, S, Cl, etc.) within the instrument’s mass‑accuracy tolerance. For many peaks, a unique molecular formula can be assigned, and for some we can also propose a likely compound identity.**

**However, it is typical in PTR‑TOF datasets that a proportion of ions cannot be assigned a definitive formula. This occurs for several reasons:**

- **the ion may be a fragment rather than a parent molecule;**
- **the ion may contain elements not included in the formula‑matching constraints;**
- **multiple formulas may fall within the mass‑accuracy window with no unique solution;**
- **the compound may represent a low‑abundance or poorly characterised VOC not present in reference libraries.**

**For transparency, and in line with standard PTR‑MS reporting practice, we therefore list these peaks as “unknown” while still including their accurate monoisotopic masses so that they can be interpreted consistently across studies.**

**We have also updated the table caption to make this clear.**

**“***Table 1. Summary of the top 15 PTR‑QiTOF ions elevated above the healthy 95% confidence interval, listed by decreasing prevalence across diseased individuals. For each ion, we report the monoisotopic m/z, the molecular formula (when a unique formula could be assigned), a putative identification (where possible), the total number of diseased samples in which an exceedance occurred, the number of diseased individuals contributing those exceedances, and the counts by disease day (D1–D5) post‑diagnosis. Ions labelled “unknown” are peaks for which no unambiguous formula match could be made (e.g. fragment ions or compositions outside the element set used for matching), which is typical for high‑resolution PTR‑MS datasets.”*

*Reviewer #2: use generic name*

**AR: This has been changed**

*Reviewer #2: Could it be that ketones are elevated because sick calves often go off feed?*

**AR: Yes – the reviewer makes a good point, which we have worked into the revised manuscript:**

*“However, it is important to acknowledge that these VOCs are not specific to BRD. Acetone, for instance, is a well-established biomarker of ketosis,* ***and short‑term reductions in feed intake during illness can induce negative energy balance and raise breath acetone independently of respiratory pathology.*** *More broadly, both acetone and formaldehyde are associated with oxidative stress and systemic inflammation.”*
