# Supplementary material for: Antibiotics confound breath-based respiratory disease detection in calves
Source: PLoS One. 2026 Jun 24;21(6):e0351838. doi: 10.1371/journal.pone.0351838 (PMC13293443; doi:10.1371/journal.pone.0351838)
Supplement: Peer review history

# Peer review: Decision Letter 0

**DOI:** 10.1371/journal.pone.0351838.r001

**Authors:** Angel Abuelo

5 Mar 2026

PONE-D-25-67033

Antibiotics Confound Breath-Based Respiratory Disease Detection in Calves

PLOS One

Dear Dr. Langford,

Thank you for submitting your manuscript to PLOS ONE. After careful consideration, we feel that it has merit but does not fully meet PLOS ONE’s publication criteria as it currently stands. Therefore, we invite you to submit a revised version of the manuscript that addresses the points raised during the review process.

Major criticisms were raised about the study and the manuscript presentation. Please revise the manuscript in light of both reviewers' comments to see if the issues identified can be corrected at this stage.

Please submit your revised manuscript by Apr 19 2026 11:59PM. If you will need more time than this to complete your revisions, please reply to this message or contact the journal office at plosone@plos.org. When you're ready to submit your revision, log on to https://www.editorialmanager.com/pone/ and select the 'Submissions Needing Revision' folder to locate your manuscript file.

Please include the following items when submitting your revised manuscript:

- A letter that responds to each point raised by the academic editor and reviewer(s). You should upload this letter as a separate file labeled 'Response to Reviewers'.
- A marked-up copy of your manuscript that highlights changes made to the original version. You should upload this as a separate file labeled 'Revised Manuscript with Track Changes'.
- An unmarked version of your revised paper without tracked changes. You should upload this as a separate file labeled 'Manuscript'.

If you would like to make changes to your financial disclosure, please include your updated statement in your cover letter. Guidelines for resubmitting your figure files are available below the reviewer comments at the end of this letter.

If applicable, we recommend that you deposit your laboratory protocols in protocols.io to enhance the reproducibility of your results. Protocols.io assigns your protocol its own identifier (DOI) so that it can be cited independently in the future. For instructions see: https://journals.plos.org/plosone/s/submission-guidelines#loc-laboratory-protocols. Additionally, PLOS ONE offers an option for publishing peer-reviewed Lab Protocol articles, which describe protocols hosted on protocols.io. Read more information on sharing protocols at https://plos.org/protocols?utm_medium=editorial-email&utm_source=authorletters&utm_campaign=protocols.

We look forward to receiving your revised manuscript.

Kind regards,

Angel Abuelo, DVM, MRes, MSc, PhD, DABVP (Dairy), DECBHM

Academic Editor

PLOS One

Journal Requirements:

When submitting your revision, we need you to address these additional requirements.

1. Please ensure that your manuscript meets PLOS ONE's style requirements, including those for file naming. The PLOS ONE style templates can be found at

https://journals.plos.org/plosone/s/file?id=wjVg/PLOSOne_formatting_sample_main_body.pdf and

https://journals.plos.org/plosone/s/file?id=ba62/PLOSOne_formatting_sample_title_authors_affiliations.pdf.

2. To comply with PLOS One submissions requirements, in your Methods section, please provide additional information regarding the experiments involving animals and ensure you have included details on (1) methods of sacrifice, (2) methods of anesthesia and/or analgesia, and (3) efforts to alleviate suffering.

3. Thank you for stating the following financial disclosure:

“This work was funded as part of the Innovate UK grant (Project # 10072590): DETECT.”

Please state what role the funders took in the study.  If the funders had no role, please state: "The funders had no role in study design, data collection and analysis, decision to publish, or preparation of the manuscript."

If this statement is not correct you must amend it as needed.

Please include this amended Role of Funder statement in your cover letter; we will change the online submission form on your behalf.

4. Thank you for stating the following in the Acknowledgments Section of your manuscript:

“This work was funded as part of the Innovate UK grant (Project # 10072590): DETECT.”

We note that you have provided additional information within the Acknowledgements Section that is not currently declared in your Funding Statement. Please note that funding information should not appear in the Acknowledgments section or other areas of your manuscript. We will only publish funding information present in the Funding Statement section of the online submission form.

Please remove any funding-related text from the manuscript and let us know how you would like to update your Funding Statement. Currently, your Funding Statement reads as follows:

“This work was funded as part of the Innovate UK grant (Project # 10072590): DETECT.”

Please include your amended statements within your cover letter; we will change the online submission form on your behalf.

5. Please include captions for your Supporting Information files at the end of your manuscript, and update any in-text citations to match accordingly. Please see our Supporting Information guidelines for more information: http://journals.plos.org/plosone/s/supporting-information.

6. If the reviewer comments include a recommendation to cite specific previously published works, please review and evaluate these publications to determine whether they are relevant and should be cited. There is no requirement to cite these works unless the editor has indicated otherwise.

[Note: HTML markup is below. Please do not edit.]

Reviewers' comments:

Reviewer's Responses to Questions

**Comments to the Author**

1. Is the manuscript technically sound, and do the data support the conclusions?

The manuscript must describe a technically sound piece of scientific research with data that supports the conclusions. Experiments must have been conducted rigorously, with appropriate controls, replication, and sample sizes. The conclusions must be drawn appropriately based on the data presented.

Reviewer #1: Partly

Reviewer #2: Yes

**********

2. Has the statistical analysis been performed appropriately and rigorously?

Reviewer #1: Yes

Reviewer #2: Yes

**********

3. Have the authors made all data underlying the findings in their manuscript fully available?

The PLOS Data policy requires authors to make all data underlying the findings described in their manuscript fully available without restriction, with rare exception (please refer to the Data Availability Statement in the manuscript PDF file). The data should be provided as part of the manuscript or its supporting information, or deposited to a public repository. For example, in addition to summary statistics, the data points behind means, medians and variance measures should be available. If there are restrictions on publicly sharing data—e.g. participant privacy or use of data from a third party—those must be specified.

Reviewer #1: Yes

Reviewer #2: Yes

**********

4. Is the manuscript presented in an intelligible fashion and written in standard English?

PLOS ONE does not copyedit accepted manuscripts, so the language in submitted articles must be clear, correct, and unambiguous. Any typographical or grammatical errors should be corrected at revision, so please note any specific errors here.

Reviewer #1: No

Reviewer #2: Yes

**********

5. Review Comments to the Author

Please use the space provided to explain your answers to the questions above. You may also include additional comments for the author, including concerns about dual publication, research ethics, or publication ethics. (Please upload your review as an attachment if it exceeds 20,000 characters)

Reviewer #1: The authors measured several volatile organic compounds (VOCs) in exhaled breath using a PRT-Qi-TOF and volatile gas analyzers. Healthy and suspected ill animals were categorized using a modified Wisconsin scoring system. The VOC results showed the levels in breath depend on calf age, time of collection, and health status. Several of these VOCs appeared to be affected by antibiotic administration. Since antibiotic administration is common when animals are suspected of respiratory disease, this constitutes a confounder for BRD diagnosis using VOCs. This finding is significant as it may hinder the applicability of VOC analysis for BRD diagnosis. In general, the data is of acceptable quality with relevant statistical analyses. Results are organized into understandable figures and tables. Though many control measurements were performed, additional are needed to support the validity of sulfur-containing VOCs as BRD biomarkers. However, the text associated with these results is very difficult to understand. This significantly hinders the readability of the manuscript and needs to be heavily revised. There are grammatical and formatting errors throughout the text that need to be addressed. The manuscript is poorly cited. Relevant research exists in the literature that the authors need to include in the introduction and results sections of the manuscript. Furthermore, the authors need to be judicious when citing to ensure in-text claims are truly supported by literature. Several references that the authors cited do not include the content they claim. The manuscript presents important results that contribute to the field of exhaled breath analysis and BRD diagnosis. However, the manuscript preparation hinders the communication of the findings.

Positives:

• Measuring four replicate blanks followed by four replicate samples over two minutes is a good approach to increase confidence in the results. Similar benefit for Figure 6, where the four volatiles were measured for two days before antibiotic administration.

• The data are well summarized in the figures. The plots are well organized with color coding that helps with visualization.

Criticisms:

• Add a diagram of the experimental procedure, including the online breath sampling setup for PTR-QiTOF, MIRO MGA10-GP, and 720 VOC Analyzer. This helps readers visualize the breath collection process.

• Define the temperature units used (e.g. °C)

• Clarify the methodology throughout section 2. The procedures are difficult to understand. This is especially true for section 2.4, though not exclusively.

• Figure 1 x-axis is confusing. At first glance, it appears to be fractions of a minute until reading the caption. Please clarify this.

• Listing the full calf number designations is confusing and hard to follow. Please simply the assignments for each animal.

• In the text, explain why there are more breath samples in the pre-antibiotic group compared to the seven healthy calves treated with antibiotics in the Figure 5 PCA analysis.

• Clarify the p-values for both DMS and methanethiol. In the text, only one p-value is listed at the two listed time points (i.e., 1 h & 24 h). For two compounds, this should be four total p-values (2 compounds × 2 time points).

• Since DMS is proposed as an important exhaled VOC, the authors need to provide additional evidence that the DMS does not originate from formaldehyde sulfoxylate. At present, the authors only state that it is “plausible” that the sulfur-containing VOCs originate from the antibiotic formulation.

• Though the findings in Section 3.6 and Figure 8 are interesting, they are preliminary. The data expand the scope of the paper beyond what is appropriate. It could be included in a follow-up paper, if desired, with additional subjects.

• Throughout the paper, the wording is confusing and hard to follow. Many paragraphs are wordy and the conclusions are not clear. This needs to be rectified.

Comments on citations:

• There are more recent original research and literature reviews discussing exhaled breath analysis for disease diagnosis in both humans and animals. Such recent works should be included in the first paragraph of the introduction.

• The content in citations 12 and 13 do not match the text with which they are associated. Specifically, there is no discussion of the economic impact of BRD in the listed sources. The authors need to support this claim with other sources. Citations 12 and 13 are important pieces of literature. It would be good to include them elsewhere in the introduction.

• Citation 17 does not discuss how calf pneumonia creates “management burdens”. The authors need to (i) reference a more appropriate source and/or (ii) to be more specific with the claim in the text.

**These comments are also included in the uploaded document**

Reviewer #2: This manuscript is an interesting analysis of the effects of antimicrobial therapy on breath VOCs in calves affected with BRD. Please see the attached PDF of your manuscript with my comments.

Some additional comments: it may be useful to include a visual diagram of the experiment, showing sampling times.

Comment on supplemental figures: animals are referred to as cows in all figure captions, please change this to "calf."

**********

6. PLOS authors have the option to publish the peer review history of their article (what does this mean?). If published, this will include your full peer review and any attached files.

If you choose “no”, your identity will remain anonymous but your review may still be made public.

**Do you want your identity to be public for this peer review?**  For information about this choice, including consent withdrawal, please see our Privacy Policy.

Reviewer #1: No

Reviewer #2: No

**********

[NOTE: If reviewer comments were submitted as an attachment file, they will be attached to this email and accessible via the submission site. Please log into your account, locate the manuscript record, and check for the action link "View Attachments". If this link does not appear, there are no attachment files.]

To ensure your figures meet our technical requirements, please review our figure guidelines: https://journals.plos.org/plosone/s/figures

You may also use PLOS’s free figure tool, NAAS, to help you prepare publication quality figures: https://journals.plos.org/plosone/s/figures#loc-tools-for-figure-preparation.

NAAS will assess whether your figures meet our technical requirements by comparing each figure against our figure specifications.

Attachment Submitted filename: Reviewer Comments for PONE-D-25-67033.pdf

Attachment Submitted filename: PONE-D-25-67033 - comments.pdf

# Author comment: Author response to Decision Letter 1

**DOI:** 10.1371/journal.pone.0351838.r002

23 Apr 2026

Please see the uploaded response document which gives a line-by-line response to each of the reviewer's concerns

Attachment Submitted filename: Response.docx

# Peer review: Decision Letter 1

**DOI:** 10.1371/journal.pone.0351838.r003

**Authors:** Angel Abuelo

2 Jun 2026

Antibiotics Confound Breath-Based Respiratory Disease Detection in Calves

PONE-D-25-67033R1

Dear Dr. Langford,

We’re pleased to inform you that your manuscript has been judged scientifically suitable for publication and will be formally accepted for publication once it meets all outstanding technical requirements.

Within one week, you’ll receive an e-mail detailing the required amendments. When these have been addressed, you’ll receive a formal acceptance letter and your manuscript will be scheduled for publication.

An invoice will be generated when your article is formally accepted. Please note, if your institution has a publishing partnership with PLOS and your article meets the relevant criteria, all or part of your publication costs will be covered. Please make sure your user information is up-to-date by logging into Editorial Manager at Editorial Manager® and clicking the ‘Update My Information' link at the top of the page. For questions related to billing, please contact billing support.

If your institution or institutions have a press office, please notify them about your upcoming paper to help maximize its impact. If they’ll be preparing press materials, please inform our press team as soon as possible -- no later than 48 hours after receiving the formal acceptance. Your manuscript will remain under strict press embargo until 2 pm Eastern Time on the date of publication. For more information, please contact onepress@plos.org.

Kind regards,

Angel Abuelo, DVM, MRes, MSc, PhD, DABVP (Dairy), DECBHM

Academic Editor

PLOS One

Additional Editor Comments (optional):

Reviewers' comments:

Reviewer's Responses to Questions

**Comments to the Author**

1. If the authors have adequately addressed your comments raised in a previous round of review and you feel that this manuscript is now acceptable for publication, you may indicate that here to bypass the “Comments to the Author” section, enter your conflict of interest statement in the “Confidential to Editor” section, and submit your "Accept" recommendation.

Reviewer #2: All comments have been addressed

**********

2. Is the manuscript technically sound, and do the data support the conclusions?

The manuscript must describe a technically sound piece of scientific research with data that supports the conclusions. Experiments must have been conducted rigorously, with appropriate controls, replication, and sample sizes. The conclusions must be drawn appropriately based on the data presented.

Reviewer #2: Yes

**********

3. Has the statistical analysis been performed appropriately and rigorously?

Reviewer #2: Yes

**********

4. Have the authors made all data underlying the findings in their manuscript fully available?

The PLOS Data policy requires authors to make all data underlying the findings described in their manuscript fully available without restriction, with rare exception (please refer to the Data Availability Statement in the manuscript PDF file). The data should be provided as part of the manuscript or its supporting information, or deposited to a public repository. For example, in addition to summary statistics, the data points behind means, medians and variance measures should be available. If there are restrictions on publicly sharing data—e.g. participant privacy or use of data from a third party—those must be specified.

Reviewer #2: Yes

**********

5. Is the manuscript presented in an intelligible fashion and written in standard English?

PLOS ONE does not copyedit accepted manuscripts, so the language in submitted articles must be clear, correct, and unambiguous. Any typographical or grammatical errors should be corrected at revision, so please note any specific errors here.

Reviewer #2: Yes

**********

6. Review Comments to the Author

Please use the space provided to explain your answers to the questions above. You may also include additional comments for the author, including concerns about dual publication, research ethics, or publication ethics. (Please upload your review as an attachment if it exceeds 20,000 characters)

Reviewer #2: (No Response)

**********

7. PLOS authors have the option to publish the peer review history of their article (what does this mean?). If published, this will include your full peer review and any attached files.

If you choose “no”, your identity will remain anonymous but your review may still be made public.

**Do you want your identity to be public for this peer review?**  For information about this choice, including consent withdrawal, please see our Privacy Policy.

Reviewer #2: No

**********

# Editor report: Acceptance letter

**DOI:** 10.1371/journal.pone.0351838.r004

**Authors:** Angel Abuelo

PONE-D-25-67033R1

PLOS One

Dear Dr. Langford,

I'm pleased to inform you that your manuscript has been deemed suitable for publication in PLOS One. Congratulations! Your manuscript is now being handed over to our production team.

At this stage, our production department will prepare your paper for publication. This includes ensuring the following:

* All references, tables, and figures are properly cited

* All relevant supporting information is included in the manuscript submission,

* There are no issues that prevent the paper from being properly typeset

You will receive further instructions from the production team, including instructions on how to review your proof when it is ready. Please keep in mind that we are working through a large volume of accepted articles, so please give us a few days to review your paper and let you know the next and final steps.

Lastly, if your institution or institutions have a press office, please let them know about your upcoming paper now to help maximize its impact. If they'll be preparing press materials, please inform our press team within the next 48 hours. Your manuscript will remain under strict press embargo until 2 pm Eastern Time on the date of publication. For more information, please contact onepress@plos.org.

You will receive an invoice from PLOS for your publication fee after your manuscript has reached the completed accept phase. If you receive an email requesting payment before acceptance or for any other service, this may be a phishing scheme. Learn how to identify phishing emails and protect your accounts at https://explore.plos.org/phishing.

If we can help with anything else, please email us at customercare@plos.org.

Thank you for submitting your work to PLOS ONE and supporting open access.

Kind regards,

PLOS ONE Editorial Office Staff

on behalf of

Dr. Angel Abuelo

Academic Editor

PLOS One
